# Bone-derived Osterix+ osteolineage cells are a source of tumor-promoting myofibroblastic cancer-associated fibroblasts in breast cancer

**DOI:** 10.1038/s41467-026-73980-7

**Published:** 2026-06-11

**Authors:** Giulia Furesi, Carisa Zeng, Emily M. Eul, Jennifer Zarrer, Deborah J. Veis, Jiayu Ye, Vasilios A. Morikis, Taylor Malachowski, Darya Khantakova, Alina Ulezko Antonova, Anupama Melam, Marco Colonna, Eric Hesse, Hanna Taipaleenmäki, Gregory D. Longmore, Sheila A. Stewart, Maxim N. Artyomov, Roberta Faccio

**Affiliations:** 1 Department of Orthopaedic Surgery, Washington University School of Medicine, St. Louis, Missouri USA; 2 Department of Pathology and Immunology, Washington University School of Medicine, St. Louis, Missouri USA; 3 Department of Developmental Biology, Washington University School of Medicine, St. Louis, Missouri USA; 4 Institute of Musculoskeletal Medicine, Musculoskeletal University Center Munich, University Hospital, LMU Munich, Munich Germany; 5 Department of Medicine, Washington University School of Medicine, St. Louis, Missouri USA; 6 Siteman Cancer Center, Washington University School of Medicine in St. Louis, St. Louis, Missouri USA; 7 Shriners Hospitals for Children, St. Louis, Missouri USA; 8 Department of Cell Biology and Physiology, Washington University School of Medicine, St. Louis, Missouri USA; 9 Bursky Center for Human Immunology and Immunotherapy Programs, School of Medicine, Washington University in St. Louis, St Louis, MO USA

**Keywords:** Cancer microenvironment, Breast cancer, Bone cancer

## Abstract

Cancer-associated fibroblasts (CAFs) are major regulators of breast cancer (BC) progression and therapeutic resistance, yet the extent to which CAF heterogeneity is dictated by distinct cellular origins remains unresolved. Here, we identify bone-derived Osterix^+^ (Osx) osteolineage cells as a source of CAFs in BC. Using BC models in female mice and biopsies from women with BC, we show that bone-resident Osx^+^ cells are recruited to primary tumors. These cells preferentially differentiate into a myofibroblastic CAF subset with unique osteolineage identity (OsteoLin-myCAFs). OsteoLin-myCAFs are transcriptionally and functionally distinct from other subsets, exhibit enhanced extracellular matrix remodeling and pronounced pro-tumorigenic activity. Mechanistically, Osx drives expression of matrix-remodeling programs, including MMP13, which supports tumor growth. Cross-species analyses show a conserved 54-gene osteolineage signature in myCAFs from human BC samples, strongly associated with poor survival. Together, these findings identify a distinct bone-derived osteolineage cell that gives rise to OsteoLin-myCAFs and is linked to adverse clinical outcomes.

## Abstract (web summary)

Cancer associated fibroblasts (CAFs) are important regulators of breast cancer (BC) progression. Here the authors identify a distinct pro tumorigenic myofibroblastic CAF population that derives from bone-resident Osterix+ osteolineage cells and retains osteolineage features in the tumor microenviroment.

## Introduction

Breast cancer (BC) remains the most frequently diagnosed cancer in women, accounting for ~30% of new cases each year in the United States. While genetic and molecular alterations within cancer cells are key drivers of tumorigenesis^1,2^, the tumor microenvironment (TME) plays an equally crucial role in shaping tumor progression and response to therapy^3,4^. Among stromal populations, cancer-associated fibroblasts (CAFs) are major regulators of extracellular matrix (ECM) remodeling, immune modulation, and resistance to therapy^5–9^. Increased ECM deposition and stiffness support tumor cell proliferation and invasion^10^, while CAF-derived cytokines and growth factors further restrict anti-tumor immune responses and promote tumor cell survival^11,12^. Consistent with these functions, CAF abundance is associated with poor clinical outcomes in BC and other tumor types^13,14^.

Despite their relevance to tumor progression, the development of effective CAF-targeted therapies has been limited due to marked CAF heterogeneity. Transcriptional profiling and scRNA-seq studies have classified CAFs into distinct subsets: myofibroblastic (myCAFs) and matrix-producing CAFs (mCAFs), involved in ECM production and remodeling^15,16^; inflammatory (iCAFs), antigen-presenting (apCAFs), and senescent CAFs (senCAFs), linked to immune responses^17–20^; vascular CAFs (vCAFs), which support vascular growth, and additional specialized CAF subsets based on their specific functionality and/or tissue of origin^16,21,22^. Yet it is unclear whether the CAF heterogeneity reflects the functional plasticity of a common resident fibroblast progenitor or arises from distinct cellular origins. Distinguishing between these possibilities is critical for the design of strategies that selectively target tumor-promoting CAF subsets.

Evidence supporting both a single universal CAF progenitor and multiple cellular origins exists. Tissue-resident fibroblasts can act as conserved progenitors capable of adopting multiple CAF subtypes, underscoring their phenotypic adaptability^23^. Conversely, CAFs have been proposed to originate from different sources, including bone marrow mesenchymal stromal cells (BMSC), adipocytes, pericytes, epithelial and/or endothelial cells, and even cancer stem cells^24–29^. This variability might be cancer-type and/or cancer-stage specific^14^. Whether the origin of CAFs dictates their phenotype/functionality in the TME remains largely unexplored.

Osteoblasts are bone-resident populations derived from BMSC, specialized in bone formation^30^. Given the functional similarities between osteoblasts and CAFs, particularly related to ECM synthesis and remodeling, we hypothesized that committed osteolineage cells could contribute to generating CAF populations in the TME. Osterix (Osx) is a transcription factor expressed by osteolineage cells, required for osteoblast differentiation and bone deposition, and its deletion results in perinatal lethality due to failed skeletal ossification^31,32^. By using lineage tracing mouse models, the presence of a few Osx^+^ cells and/or their progeny has been detected outside of the bone, including in solid tumors^33,34^. However, the potential contribution of Osx-derived stromal cells to tumor progression hasn’t been reported. In this work, we identify a distinct myCAF population that expresses Osx, retains an osteolineage transcriptional identity, exhibits potent pro-tumorigenic effects, and is linked to adverse clinical outcomes in BC patients.

## Results

### Osteolineage-derived OsxTdT^+^ cells migrate from the bone marrow to the TME to enhance tumor growth

**Fig. 1:**
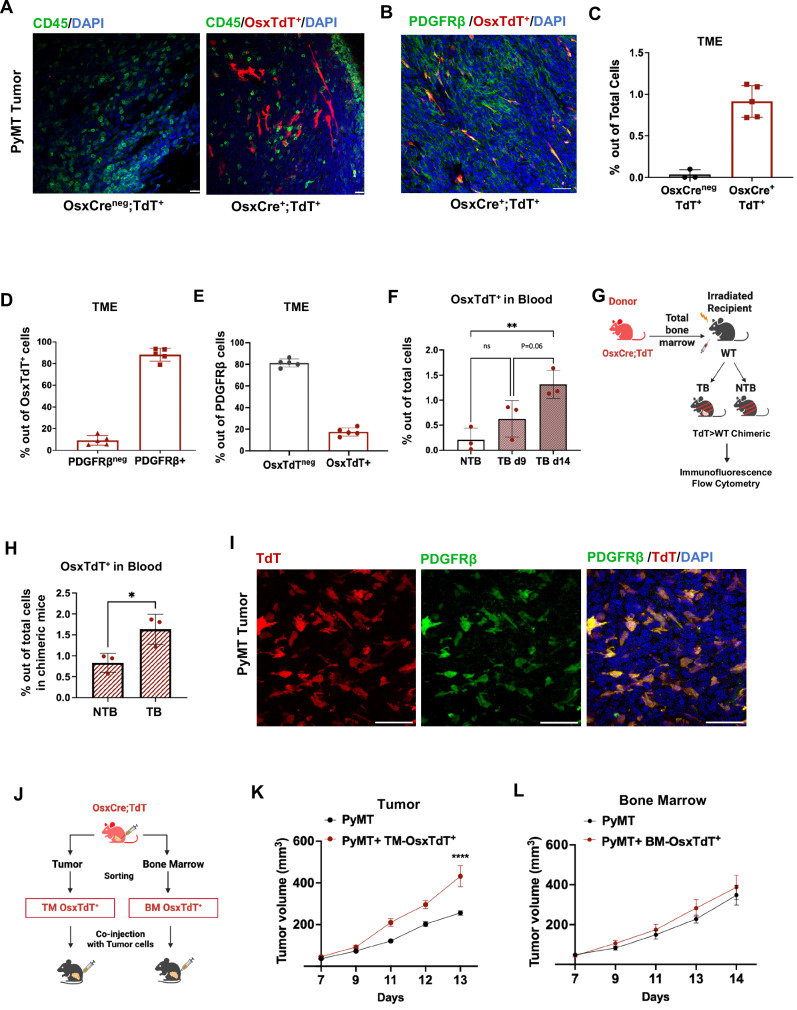
Osteolineage-derived OsxTdT^+^ cells migrate from the bone marrow to the TME to enhance tumor growth. **A**,** B** Representative z-stack immunofluorescence (IF) images of PyMT tumors from OsxCre^neg^;TdT⁺ and OsxCre⁺;TdT⁺ mice. (A) OsxTdT⁺ cells (red), CD45⁺ cells (green), and DAPI (blue). **B** OsxTdT⁺ cells (red), PDGFRβ⁺ cells (green), and DAPI (blue). Scale bars, 20 µm (**A**) and 50 µm (**B**). *n* = 3. **C**–**E** Flow cytometric analysis showing (**C**) the percentage of OsxTdT⁺ cells in PyMT-BO1-GFP^+^ orthotopic tumors from OsxCre^neg^;TdT^+^ (*n* = 3) and OsxCre^+^;TdT^+^ mice (*n* = 5), (**D**) the percentage of PDGFRβ⁺ cells among OsxTdT⁺ cells and (**E**) the percentage of OsxTdT⁺ cells among PDGFRβ⁺ cells in PyMT-BO1-GFP^+^ orthotopic tumors from OsxCre^+^;TdT^+^ (*n* = 5). **F** Frequency of circulating OsxTdT⁺ cells in non-tumor-bearing (NTB) and tumor-bearing (TB) OsxCre⁺;TdT⁺ mice detected 9 and 14 days following orthotopic PyMT tumor inoculation (*n* = 3). **G** Schematic of bone marrow transplantation. OsxCre⁺;TdT⁺ donor bone marrow was transferred into irradiated CD45.1WT mice. After 6 weeks, TdT>WT chimeric mice were injected orthotopically with PyMT cells and analyzed by IF and flow cytometry to track donor-derived OsxTdT⁺ cells. **H** Flow cytometric analysis showing the percentage of CD45^neg^OsxTdT^+^ in blood from NTB (*n* = 3) or TB (*n* = 3) TdT>WT chimeric mice. **I** Representative z-stack IF images of PyMT tumors (*n* = 3) from TdT>WT mice stained for PDGFRβ (green) and DAPI (blue) with OsxTdT^+^ cells in red. Scale bar =50μM. **J** Experimental scheme for isolation of CD45^neg^OsxTdT⁺ stromal cells from tumor-derived (TM) CAF-enriched cultures or bone marrow (BM) and used in co-injection studies. **K**,**L** Tumor growth determined by caliper measurements in WT mice inoculated with PyMT tumor cells alone or co-injected with TM-derived (**K**, *n* = 3/group) or BM-derived (**L**, *n* = 5/group) CD45^neg^OsxTdT^+^ cells at 1:1 stroma:tumor ratio. Results are shown as mean ± SD (**C**–**F**, **H**) and ± SEM (**K**, **L**). Statistical significance was determined by one-way ANOVA with Tukey’s test (**F**), unpaired two-tailed Student’s t-test (**H**), or two-way ANOVA with Sidak’s multiple comparisons test (**K**–**L**). Significance is denoted as *P ≤ 0.05, ***P ≤ 0.001, ****P ≤ 0.0001. Source data and exact *P*-values are provided as a Source Data file. Figures 1G and 1J were created in BioRender. Furesi, G. (https://BioRender.com/rpuownu).

To investigate whether bone resident osteolineage populations contribute to the CAF pool in breast tumors, we used the doxycycline (doxy)-repressible OsxCre;TdT lineage tracing mouse model^35^. Mice were fed a doxy diet until weaning to activate Cre in committed osteoblast progenitors in bone marrow (BM), mature osteoblasts, and osteocytes. 8-week-old OsxCre;TdT⁺ female mice were then orthotopically inoculated with the luminal B, ER⁺/PR⁺, hormone-resistant PyMT cell line. Two weeks after tumor implantation, immunofluorescence (IF) staining of the isolated tumor mass revealed presence of CD45^neg^OsxCre;TdT⁺ cells (hereafter referred to as OsxTdT^+^) infiltrating the TME, with an elongated, spindle-shaped morphology (Fig. 1A). Phenotypic characterization showed that these cells colocalized with PDGFRβ, a pan-mesenchymal and CAF marker (Fig. 1B, unstained controls in Supplementary Fig. 1A). Flow cytometric analysis 10 days post PyMT-BO1-GFP^+^ tumor inoculation revealed that OsxTdT⁺ cells comprised ~0.8–1.2% of the total tumor (Fig. 1C; and Supplementary Fig. 1B). Notably, over 80% of OsxTdT⁺ cells expressed the stromal marker PDGFRβ (Fig. 1D). In contrast, PDGFRβ⁺OsxTdT⁺ cells accounted for only ~20% of the total PDGFRβ⁺ stromal populations within the TME (Fig. 1D, and Supplementary Fig. 1B), suggesting that OsxTdT⁺ cells represent a distinct subset of tumor-infiltrating mesenchymal cells.

To determine whether OsxTdT⁺ cells in the TME derive from locally resident Osx^+^ cells, we first performed IF staining of the healthy mammary gland from 6-week-old OsxCre⁺;TdT⁺ mice, following Cre activation at weaning as described above. Only rare OsxTdT⁺ cells were detected before tumor implantation (Supplementary Fig. 1C).

Next, to assess the possibility that OsxTdT⁺ cells are recruited to the TME from an osteolineage population residing in the bone, we analyzed their presence in circulation over the course of tumor progression. A very small percentage of OsxTdT⁺ cells ( ~ 0.2% of total blood cells) was present in no tumor-bearing (NTB) mice. This population expanded markedly following tumor inoculation, showing approximately a three-fold increase by day 9, and a seven-fold increase by day 14 relative to NTB controls (Fig. 1F, and Supplementary Fig. 1D).

To further determine that the tumor-infiltrating OsxTdT⁺ cells are mobilized from the BM, we transplanted BM from OsxCre⁺;TdT⁺ mice (CD45.2) into naïve, age and sex-matched lethally irradiated wild-type (WT) recipients (CD45.1) (model Fig. 1G). In contrast to classical bone marrow transplantation (BMT) assays used for hematopoietic purposes, we transplanted five times the standard amount of BM cells to ensure the transfer of mesenchymal populations. Chimerism was confirmed by the presence of over 80% CD45.2^+^ immune cells in circulation at three weeks post-transplant (Supplementary Figs. 1E, 1F). The chimeric mice were orthotopically injected with PyMT tumor cells at six weeks post-BMT, and the presence of donor-derived OsxTdT⁺ cells was assessed two weeks later in the blood and tumor mass. We observed a 1.9-fold increase in the number of OsxTdT⁺ cells in the blood of chimeric tumor-bearing mice versus chimeric NTB controls (Fig. 1H). Importantly, we detected CD45^neg^OsxTdT⁺ cells in the tumor mass that colocalized with PDGFRβ^+^ populations (Fig. 1I, and Supplementary Fig. 1G). These results indicate that tumor-infiltrating mesenchymal OsxTdT⁺ cells originate from an Osx^+^ osteolineage cell in the BM.

Next, to assess the functional contribution of OsxTdT⁺ cells to tumor growth, we isolated CD45^neg^;OsxTdT⁺ cells from orthotopic PyMT tumors in OsxCre⁺;TdT⁺ mice. Tumors were enzymatically dissociated and plated on tissue culture dishes for 30 min to enrich for adherent stromal cells, after which non-adherent tumor and immune populations were removed^11^. Adherent cells were cultured for an additional 16 h and subsequently sorted based on TdT expression and exclusion of CD45. Isolated OsxTdT⁺ cells were co-injected with PyMT tumor cells at a 1:1 stroma-to-tumor ratio into age-matched WT recipient female mice (Fig. 1J). In parallel, OsxTdT⁺ cells were also isolated from the BM of tumor-bearing OsxCre⁺;TdT⁺ mice following CD45⁺ cell depletion to remove hematopoietic populations (Fig. 1J). Notably, cancer cells co-injected with tumor-derived OsxTdT⁺ cells grew faster than PyMT cells alone, while co-injection with BM-derived OsxTdT⁺ cells did not give any growth advantage (Figs. 1K, 1L). These findings indicate that tumor-infiltrating OsxTdT⁺ cells support BC growth.

### Tumor-infiltrating OsxTdT⁺ cells comprise a distinct osteolineage myCAF population

**Fig. 2:**
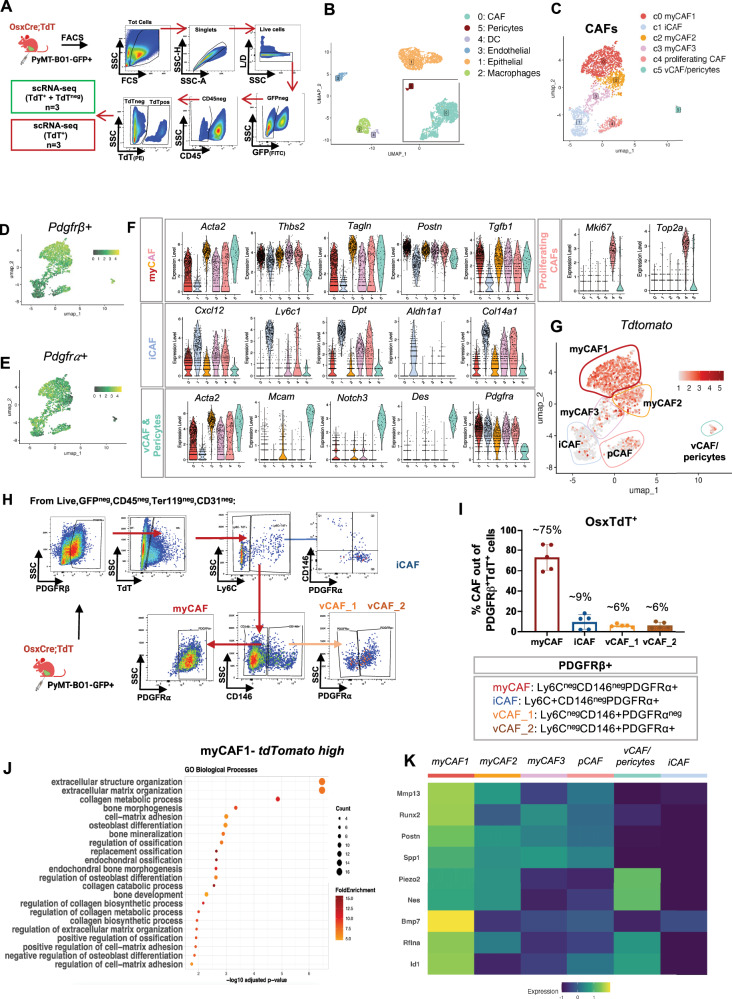
Tumor infiltrating OsxTdT⁺ cells comprise a distinct osteolineage myCAF population. **A** Gating strategy used to isolate GFP^neg^CD45^neg^OsxTdT⁺ stromal cells (*n* = 3) or GFP^neg^ CD45^neg^ stromal populations (*n* = 3) from PyMT-BO1-GFP⁺ orthotopic tumors in OsxCre⁺TdT⁺ mice. **B** UMAP of the combined murine scRNA-seq dataset from sorted populations in A showing cluster identity. **C** UMAP showing subclustering of CAF subpopulations. **D**,**E** Feature plots showing the expression of *Pdgfrβ* and *Pdgfrα* in CAF subclusters. **F** Violin plots of CAF subset markers. **G** Feature plot showing *TdTomato* expression (red) in the murine CAF subsets.** H**,**I** Gating strategy (**H**) and characterization by flow cytometry (**I**) of OsxTdT⁺ CAF subpopulations isolated from PyMT-BO1-GFP^+^ tumors in OsxCre^+^;TdT^+^ mice (*n* = 5). CAFs were identified as GFP^neg^, CD45^neg^, Ter119^neg^, CD31^neg^ and PDGFRβ⁺ cells. CAF cluster subtypes were defined by markers listed in the box. Source data are provided as a Source Data file. **J** Gene ontology (GO) Pathway Analysis showing enrichment of osteogenic pathways in the myCAF1 subcluster compared to other CAF subclusters. Dot size indicates the number of genes in each pathway, while dot color represents the fold enrichment of each pathway among the top 200 differentially expressed genes (DEGs). **K** Heatmap illustrating the expression of indicated osteogenic markers among indicated CAF subclusters. Figures 2A, H were created in BioRender. Furesi, G. (https://BioRender.com/rpuownu).

To define the identity of tumor-infiltrating OsxTdT⁺ cells, we first isolated the GFP^neg^CD45^neg^ stromal fraction from orthotopic PyMT-BO1-GFP⁺ tumors in OsxCre⁺;TdT⁺ mice (Fig. 2A; *n* = 3). This sorted population contained both OsxTdT⁺ and OsxTdT^neg^ mesenchymal cells, which were subjected to single-cell RNA sequencing (scRNA-seq). Because OsxTdT⁺ cells represent a relatively small fraction compared to OsxTdT^neg^ cells, to ensure adequate representation in the dataset, we also separately sorted GFP^neg^CD45^neg^OsxTdT^+^ stromal cells from pooled orthotopic tumors (*n* = 3) and performed 10x Genomics 3′ scRNA-seq (Fig. 2A).

Both datasets (total stromal populations and OsxTdT⁺ cells) were merged before analysis, subjected to batch effect corrections, and visualized using the Uniform Manifold Approximation and Projection (UMAP) algorithm. Cluster identities were manually annotated based on established expression marker profiles (Supplementary Fig. 2). We detected three major clusters of CD45^neg^ stromal cells (Fig. 2B, Supplementary Fig. 2): CAFs (cluster 0: *Pdgfrβ, Pdgfrα, Fap, Acta2)*, Pericyte/vCAF (cluster 5: *Myh11, Vtn, Mcam)*, and endothelial cells (cluster 3: *Tie1, Icam2, Flt4, Pcam1*). Despite sorting for GFP^neg^ and CD45^neg^ populations, we also identified small clusters of epithelial cells (cluster 1: *Bnc1, Krt8, Krt18, Msln*), macrophages (cluster 2: *Ptprc, Adgre1, Fcgr1, C1qb*), and dendritic cells (DC) (cluster 4: *Ptprc, Flt3, Zbtb46, Batf3*).

Using UMAP dimensionality reduction and graph-based clustering, we identified six distinct subpopulations of CAFs (Fig. 2C, Supplementary Fig. 3, and Supplementary Data 1), all of which were positive for *Pdgfrβ* and *Pdgfrα*, albeit to a different degree (Figs. 2D, E). By using additional established CAF markers detected in BC patients and the spontaneous MMTV-PyMT BC model^19,36^, we annotated these six clusters as three major sub-clusters of myCAFs (cluster 0: myCAF1, cluster 2: myCAF2, cluster 3: myCAF3), one cluster of iCAFs (cluster 1), one cluster of proliferating CAFs (cluster 4), and one of vCAFs/Pericyte (cluster 5). All myCAF clusters were marked by the expression of *Acta2*, *Thbs2, Tagln, Postpn*, and *Tgfβ1;* iCAFs by the expression of *Cxcl12, Ly6C1, Aldh1a, Dept, and Col14a;* proliferating CAFs by the expression of *Miki67* and *Top2A;* vCAFs/Pericyte by the expression of *Acta2, Mcam, Notch3, Des*, and low expression of *Pdgfrα,* (Fig. 2F). Strikingly, *tdTomato* was highly expressed in the myCAF1 subset, slightly less in myCAF2 and pericytes/vCAFs, and in very few cells across other CAF clusters (Fig. 2G).

Next, to validate the scRNA-seq findings, we performed flow cytometry of the stromal compartment isolated from orthotopic PyMT-BO1-GFP⁺ tumors in OsxCre⁺;TdT⁺ mice. Because CAFs uniformly expressed PDGFRβ (Fig. 2D), we first selected PDGFRβ⁺ cells after exclusion of tumor cells (GFP⁺), red blood cells (Ter119⁺), immune cells (CD45⁺), and endothelial cells (CD31⁺). Next, we gated on OsxTdT⁺ cells and evaluated surface markers associated with myCAFs (PDGFRβ⁺ Ly6C^neg^ CD146^neg^ PDGFRα⁺), iCAFs (PDGFRβ⁺ Ly6C⁺ CD146^neg^ PDGFRα⁺), and vCAFs (PDGFRβ⁺ Ly6C^neg^ CD146⁺ and/or PDGFRα⁺) (Fig. 2H). Confirming the scRNAseq findings, the majority of OsxTdT⁺ cells ( ~ 75%) expressed myCAF markers, whereas only a small fraction expressed iCAF ( < 10%) or vCAF ( ~ 6%) markers (Fig. 2I).

To further define the molecular identity of myCAF1 (the CAF subset with the highest *tdTomato* expression), we compared its transcriptomic profile to that of all other CAF clusters (Supplementary Fig. 3 and Supplementary Data 1). myCAF1 cells were strongly enriched for osteolineage-associated processes, including bone morphogenesis, bone development, bone mineralization, osteoblast differentiation, and ossification, in addition to pathways related to ECM organization, collagen biosynthesis, and regulation of cell-matrix adhesion (Fig. 2J).

Comparative analyses showed that osteolineage programs were largely restricted to the myCAF1 cluster relative to other CAF subsets, while myCAF2 was enriched in contractile/myofibroblastic pathways and vCAFs in vascular structure and vessel regulation programs (Supplementary Fig. 4, Supplementary Data 2-3). Consistent with these observations, myCAF1 expressed the highest levels of genes associated with osteoblast development, differentiation, and function, including *Runx2, Postn, Spp1* (OPN)*, Piezo2, Nes, Mmp13, Bmp7, Rflna, and Id1* (Fig. 2K) compared to all the other CAF clusters. As expected, given the limited sensitivity of scRNA-seq for transcription factors, we were unable to detect Osx*/Sp7* in our data set. Based on their lineage-tracing profile and osteolineage gene expression, the myCAF1 cluster was designated as osteolineage myCAFs (OsteoLin-myCAFs). Collectively, these findings identify a subset of bone-derived CAFs marked by Osx lineage tracing that retains a transcriptional signature characteristic of bone-resident Osx^+^ osteolineage cells.

### OsteoLin-myCAFs express Osx and exhibit enhanced pro-tumorigenic abilities

**Fig. 3:**
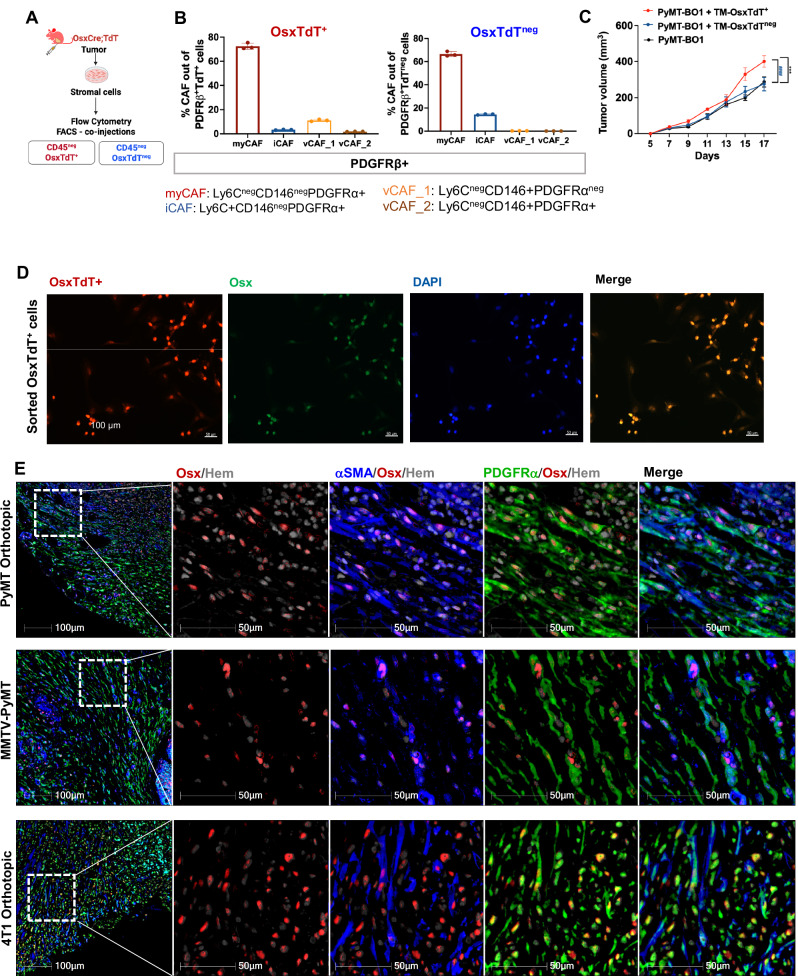
OsteoLin-myCAFs express Osx and exhibit enhanced pro-tumorigenic abilities. **A** Experimental strategy used to isolate and define OsteoLin-myCAF populations from orthotopic PyMT-BO1-GFP^+^ tumors in OsxCre^+^;TdT^+^ mice. Tumors were enzymatically dissociated and plated for 30 minutes to enrich for adherent stromal cells, followed by 16 h of culture before sorting GFP^neg^CD45^neg^OsxTdT^+^ and GFP^neg^CD45^neg^OsxTdT^neg^ cells. **B** Flow cytometric analysis for characterization of OsxTdT^+^ and OsxTdT^neg^ cells. CAF subsets were identified as GFP^neg^, CD45^neg^, Ter119^neg^, CD31^neg^ and PDGFRβ⁺ cells using the indicated markers for each subset (*n* = 3). **C** Tumor growth determined by caliper measurements in WT mice inoculated with PyMT-BO1- GFP^+^ tumor cells alone (*n* = 4) or co-injected with OsxTdT ^+^ (*n* = 5) and OsxTdT^neg^ (*n* = 5) cells from (**A**) at 1:2 stroma:tumor ratio. **D** Representative images of OsteoLin-myCAFs sorted from PyMT-BO1-GFP^+^ tumors in OsxCre;TdT^+^ mice and plated onto coverslips for IF analysis. OsxTdT^+^ cells are shown in red, Osx staining in green, DAPI in blue (*n* = 3 pooled mice). **E**, Representative deconvoluted multiplex Immunohistochemistry (mIHC) images of orthotopic PyMT, spontaneous MMTV-PyMT, and 4T1 breast tumors in WT mice (*n* = 7) stained for Osx (red), Hematoxylin (gray), αSMA (blue), PDGFRα (green). Results are shown as mean ± SD (**B**) and ± SEM (**C**). Two-way ANOVA with Tukey’s multiple-comparison test was used to determine significance in C. Significance is denoted as ***P ≤ 0.001; ^###^*P* < 0.001. Source data and exact *p* values are provided as a Source Data file. Figure 3A was created in BioRender. Furesi, G. (https://BioRender.com/rpuownu).

To assess the functional properties of OsteoLin-myCAFs relative to the other CAF populations, we used the OsxCre⁺;TdT⁺ lineage tracing mice and isolated OsxTdT^+^ and OsxTdT^neg^ CAFs from orthotopic PyMT-BO1-GFP^+^ tumors. Tumors were enzymatically dissociated, briefly plated to enrich for adherent stromal cells as described in Fig. 1J, and CAFs were isolated by sorting for CD45^neg^GFP^neg^TdT^+^ and TdT^neg^ markers (Fig. 3A, and Supplementary Fig. 5A). Consistent with the expected enrichment of myofibroblastic CAFs among adherent cells, flow cytometry analysis confirmed that 70-75% of the OsxTdT^+^ and OsxTdT^neg^ cells expressed myCAF surface markers (Ly6C^neg^CD146^neg^PDGFRα^+^). Further CAF subset analysis showed less than 20% of OsxTdT^+^ cells expressed vCAF markers (Ly6C^neg^CD146^+^PDGFRα^neg/+^) and less than 20% of OsxTdT^neg^ cells expressed iCAF markers (Ly6C^+^CD146^neg^PDGFRα^+^), consistent with the *tdTomato* distribution observed in the scRNAseq dataset (Fig. 3B, and Supplementary Fig. 5B-D). We did not observe CD31^+^ cell contamination (Supplementary Fig. 5B). Based on scRNAseq analyses, *tdTomato* expression, and enrichment of myCAF markers, we referred to the CD45^neg^GFP^neg^OsxTdT^+^ cells as OsteoLin-myCAFs.

Next, PyMT-BO1 tumor cells were co-injected with the sorted OsteoLin-myCAFs (CD45^neg^GFP^neg^OsxTdT^+^) or OsxTdT^neg^ fraction from CAF-enriched cultures into WT recipient mice at a 1:2 stroma-to-tumor ratio. Mice injected with tumor cells alone served as a control. Strikingly, OsteoLin-myCAFs exhibited markedly enhanced pro-tumorigenic activity compared with the OsxTdT^neg^ CAF fraction and tumor cells alone (Fig. 3C). To further determine whether OsteoLin-myCAFs retained Osx expression, we performed immunofluorescence analysis, and in line with Osx role as an active transcription factor, we detected Osx staining in the nuclei (Fig. 3D).

To further assess Osx expression in vivo, we performed multiplex immunohistochemistry (mIHC) using tissue sections from orthotopic PyMT, triple-negative 4T1, and spontaneous MMTV-PyMT primary breast tumors. In addition to Osx, tissues were stained for PDGFRα and αSMA as general CAF markers and cytokeratin (EPCAM) or GFP to identify tumor cells. Antibody signals were deconvolved and pseudo-colored using HALO software under the supervision of a trained pathologist (unstained and non-pseudo-colored controls shown in Supplementary Fig. 6A). Osx nuclear staining was observed in subsets of PDGFRα⁺ and αSMA⁺ stromal cells across all models (Fig. 3E). In the 4T1 and MMTV-PyMT tumors, Osx was also detected in a minority of GFP⁺ and EPCAM⁺ epithelial cells (Supplementary Fig. 6B). Because the PyMT tumor line lacks fluorescent-tagged labeling and EPCAM marker expression, we were unable to assess Osx expression in the epithelial compartment in orthotopic PyMT tumors. Altogether, these data show OsteoLin-myCAFs exhibit enhanced tumor-promoting activity and retain Osx expression in vitro and in vivo, across multiple murine BC models.

### Osx supports tumor growth and collagen remodeling in the TME

**Fig. 4:**
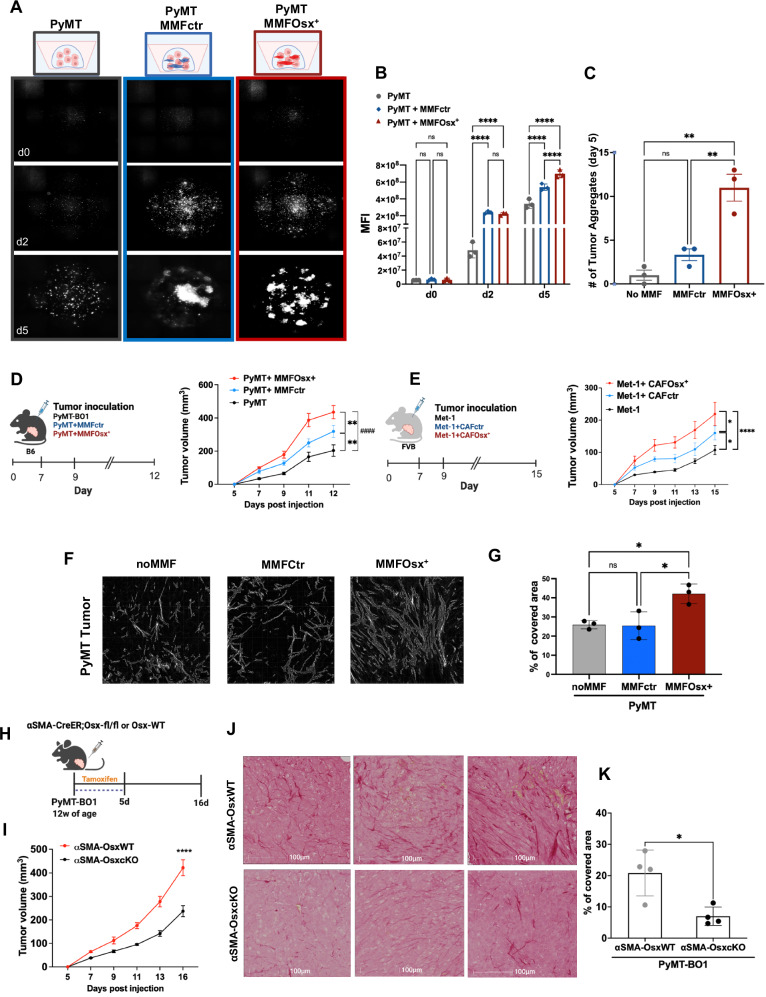
Osx supports tumor growth and collagen remodeling in the TME. **A** Schematic of PyMT-mCherry^+^ spheroids cultured alone or with MMFctr or MMFOsx^+^ cells. Representative fluorescent images of spheroids acquired on days 0, 2, and 5 after plating (*n* = 3/group). **B** Quantification of (**A**) measuring spheroid growth as maximum fluorescence intensity (MFI) over time. **C** Quantification of tumor aggregates formed at day 5 from spheroids in (**A**). **D** Schematic of orthotopic implantation of PyMT tumor cells injected alone (control, *n* = 5) or co-injected with MMFctr (*n* = 5) or MMFOsx^+^ (*n* = 6) cells at a 1:1 stroma:tumor ratio into C57BL/6 WT female mice (left). Tumor growth was monitored over time by caliper measurements (right). **E** Schematic of orthotopic implantation of Met-1 tumor cells injected alone (control, *n* = 4) or co-injected with CAFctr (*n* = 5) or CAFOsx^+^ (*n* = 4) at a 1:1 stroma:tumor ratio into FVB WT female mice (left). Tumor growth was monitored over time by caliper measurements (right). **F**,** G** Representative second harmonic generation (SHG) images (*n* = 3/group) showing collagen deposition (**F**) and quantification of collagen-covered area (**G**) in PyMT tumors or PyMT tumors co-injected with MMFOsx⁺ and MMFctr cells from (**D**). **H**,** I** Schematic of inducible Osx deletion in stromal cells using αSMA-Cre^ERT2^ mice crossed with Osx^fl/fl^ to generate αSMA;OsxcKO and αSMA;OsxWT controls. Tamoxifen was administered to 12-week-old female mice for five consecutive days, beginning at the time of orthotopic PyMT-BO1 tumor implantation (**H**). Tumor growth was monitored by caliper measurements over time (**I**) (αSMA;OsxcKO, *n *= 7; αSMA;OsxWT, *n* = 5). **J**,** K** Representative picrosirius red staining (**J**) and quantification of collagen-covered area (**K**) in tumors from αSMA;OsxcKO (*n* = 4) and αSMA;OsxWT (*n* = 4) mice. Results are shown as mean ± SD (**B**, **C**, **G**, **K**) and ± SEM (**D**, **E**, **I**). Experiments in **B** and **C** were performed in triplicate. Two-way ANOVA with Tukey’s multiple-comparison test (**B**, **D**, **E**, **I**), one-way ANOVA with Tukey’s multiple-comparison test (**C**, **G**), and an unpaired two-tailed Student T-test (**K**) were performed to determine the significance (*P ≤ 0.05, **P ≤ 0.01,***P ≤ 0.001, ****P ≤ 0.0001). Source data and exact *p*-values are provided as a Source Data file. Figures 4A, D, E, and H were created in BioRender. Furesi, G. (https://BioRender.com/rpuownu).

To understand if Osx is merely a marker of OsteoLin-myCAFs or directly contributes to their pro-tumorigenic function, we ectopically expressed Osx in murine mammary fibroblasts (MMFOsx^+^) or in an immortalized CAF cell line derived from MMTV-PyMT spontaneous tumors (FVB background; CAFOsx^+^) using pLenti-GFP-puro reporter vector (Supplementary Fig. 7A). Cells expressing empty vectors were used as controls (MMFctr and CAFctr). Infection efficiency was confirmed by detection of the GFP signal via flow cytometry and *Osx/Sp7* transcripts by quantitative real-time PCR (qRT-PCR, Supplementary Fig. 7B–D). Next, we generated 3D spheroids using mCherry^+^PyMT tumor cells alone or cultured together with MMFctr or MMFOsx^+^ (Fig. 4A). Tumor growth was quantified in real-time using live-cell fluorescence microscopy with a red channel for mCherry tumor cell detection. Similarly to mice co-injected with OsxTdT⁺ cells (Fig. 3C), PyMT spheroids grew better in the presence of MMFOsx^+^ compared to MMFctr or tumor cells alone (Fig. 4A, B). Notably, PyMT formed aggregates when cultured with MMFs, a phenomenon known to facilitate tumor progression^37^, and the MMFOsx^+^ group displayed significantly more tumor aggregates compared to controls (Fig. 4C). Next, we employed an in vivo model by co-injecting PyMT tumor cells either alone or with MMFOsx^+^ or MMFctr into eight-week-old WT female mice. MMFOsx^+^ significantly enhanced tumor growth compared to MMFctr (Fig. 4D). Similar results were obtained using the Met-1 BC cell line co-injected with CAFOsx^+^ into FVB/N female mice (Fig. 4E).

Given that Osx is a transcription factor required for bone formation and the expression of matrix proteins, we sought to understand how Osx increases CAF pro-tumorigenic properties by comparing collagen- and ECM-related genes across the CAF subsets identified in our murine scRNAseq dataset. As expected, OsteoLin-myCAFs were enriched for collagen-associated and matrix-remodeling genes (Supplementary Fig. 8A). In line with this transcriptional profile, Second Harmonic Generation (SHG) microscopy of tumors co-injected with MMFOsx^+^ cells showed a marked increase in collagen deposition compared to MMFctr or tumor cells alone (Figs. 4F, G), thus suggesting that Osx expression in OsteoLin-myCAFs contributes to ECM remodeling.

Because Osx expression is enriched in αSMA⁺ cells in the TME, we directly tested the functional contribution of Osx in the stromal compartment by generating inducible conditional knockout mice by crossing αSMA;Cre^ERT2^;tdTomato reporter mice with *Osx/Sp7*^fl/fl^ animals to generate αSMA;OsxcKOTdT⁺ and αSMA;OsxWTTdT⁺ mice, serving as controls. Tamoxifen was administered to 11-12-week-old female mice starting at the time of orthotopic PyMT-BO1 tumor implantation and continued for five consecutive days (Fig. 4H). Validation of the αSMA;OsxcKO model confirmed efficient deletion of Osx in tumor-infiltrating αSMA⁺ stromal cells by mIHC and no changes in αSMA;TdT⁺ cell frequencies in the TME by flow cytometry compared with WT littermate controls (Supplementary Fig. 8B, C).

Strikingly, deletion of Osx in αSMA⁺ stromal cells significantly reduced PyMT orthotopic tumor growth compared with controls (Fig. 4I). Consistent with a role for Osx in ECM remodeling, picrosirius red staining demonstrated decreased collagen deposition and altered collagen fiber organization in Osx-deficient tumors (Figs. 4J, 4K). These results indicate that Osx expression in OsteoLin-myCAFs modulates tumor growth possibly by altering the ECM.

### MMP13 is required for OsteoLin-myCAF pro-tumorigenic effects

**Fig. 5:**
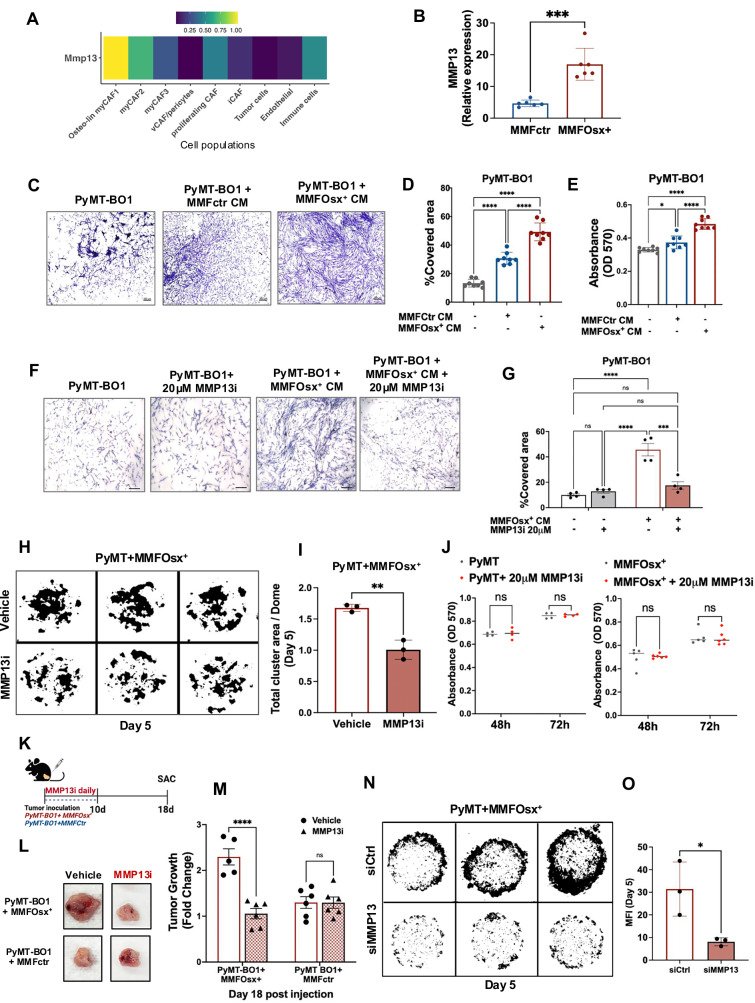
MMP13 is required for OsteoLin-myCAF pro-tumorigenic effects. **A** Heatmap illustrating *Mmp13* expression across indicated stromal populations in orthotopic PyMT-BO1-GFP^+^ tumors from OsxCre^+^;TdT^+^ mice (*n* = 6), based on murine scRNAseq data. **B** qRT-PCR analysis showing *Mmp13* transcripts in MMFOsx^+^ and MMFctr (*n* = 6/group). **C**–**E** (**C**) Representative images of PyMT-BO1-GFP^+^ tumor cells stained with crystal violet after 48 h in culture with 1% FBS control media or conditioned media (CM) collected from MMFctr and MMFOsx^+^ (Scale bar=200μM). **D** Quantification of the area occupied by tumor cells from (**C**). **E** Number of viable tumor cells cultured in control media or in CM from MMFctr or MMFOsx^+^, determined by MTT colorimetric assay. **C**–**E** *n* = 8/group. **F**, **G** Representative images of crystal violet staining of PyMT-BO1-GFP^+^ tumor cells treated for 72 h with control media or CM from MMFOsx^+^ cells, in the presence or absence of MMP13i (20 μM, Scale bar=200μM). **G** Quantification of the area occupied by the tumor cells in (**F**). **F**, **G** *n* = 4/group. **H**–**I** Representative images (**H**) and quantification (**I**) of PyMT spheroids cultured with MMFOsx⁺ cells and treated with vehicle or MMP13 inhibitor (20 µM) for 5 days (*n* = 3/group). **J**, Number of viable tumor cells (*n* = 4/group) and MMFOsx^+^ treated with vehicle (*n* = 5/group) or 20 µM MMP13i (*n* = 6/group) for 48 and 72 h determined by MTT colorimetric assay. **K** Schematic of treatment with MMP13i (0.5 mg/mouse) or vehicle in mice co-injected with MMFctr or MMFOsx^+^ cells. **L** Representative images of harvested tumors from (**M**). **M** Tumor volume was measured by caliper, with data represented as a fold change. *n* = 5 for PyMT-BO1+MMFOsx^+^ vehicle and *n* = 6 for all other groups. **N** Representative z‑stack images of PyMTmCherry^+^ spheroids co‑cultured for 5 days with MMFOsx^+^ cells transfected with *Mmp13* siRNA (si*Mmp13*) or non‑targeting control siRNA (siCtrl). **O** Quantification of spheroids is represented as mean fluorescence intensity (MFI). *n* = 3/group. Results are shown as mean ± SD. Experiments in (**B**, **D**, **E**, **I**, **J**, and **O**) were performed in triplicate. An unpaired two-tailed Student T-test (**B**, **I**, **O**), two-way ANOVA with Tukey’s multiple-comparison test (**D**, **E**, **J**, **M**), or Sidak’s multiple-comparison test (**G**) were performed to determine the significance (*P ≤ 0.05, **P ≤ 0.01,***P ≤ 0.001, ****P ≤ 0.0001). Source data and exact *p* values are provided as a Source Data file. Figure 5K was created in BioRender. Furesi, G. (https://BioRender.com/rpuownu).

Given the increased matrix deposition associated with Osx expression, we next focused on regulators of ECM remodeling rather than structural collagens (Supplementary Fig. 8A). Among matrix-modifying genes, MMP13 was selected because it is an Osx direct target and is essential for ECM remodeling in osteoblasts^38^. Furthermore, *Mmp13* was enriched in OsteoLin-myCAFs versus other subsets in our scRNA-seq dataset (Fig. 5A) and upregulated in MMFOsx⁺ compared with MMFctr by qRT-PCR (Fig. 5B).

To determine whether secreted MMP13 modulates the pro-tumorigenic effects of OsteoLin-myCAFs, PyMT-BO1 tumor cells were cultured with conditioned media (CM) collected from either MMFctr or MMFOsx⁺. Crystal violet staining and MTT assay showed increased numbers of tumor cells when cultured in MMFOsx⁺ CM, accompanied by the formation of larger and more elongated tumor cell clusters compared to cells in MMFctr CM or standard cell culture media (Fig. 5C–E). Notably, pharmacological inhibition of MMP13 using CL-82198 (20 µM), which specifically binds the S1 pocket of the enzyme and impairs its function^39^, inhibited both tumor cell growth and the morphological alterations induced by MMFOsx⁺ CM (Figs. 5F, G). In contrast, MMP13 inhibition had no effects on tumor cell numbers or morphology in standard culture conditions (Fig. 5F, G). Similarly, mCherry^+^PyMT cells cultured with MMFOsx⁺ in a 3D spheroid model showed a marked reduction in tumor cluster size when treated with the MMP13i (20 µM) (Fig. 5H, I). As a control, we confirmed that MMP13i did not alter cell numbers or morphology when added directly to either tumor cells or MMFs (Fig. 5J).

Finally, to assess the effects of MMP13i in vivo, PyMT-BO1 tumor cells were injected with MMFOsx⁺ or MMFctr into the MFP of 8-week-old WT female mice. Mice were treated with MMP13i (0.5 mg/mouse) or vehicle for 10 consecutive days, starting on the day of tumor injection, and were sacrificed 18 days post-tumor inoculation (Fig. 5K), as previously reported^40^. A significant reduction in tumor burden was detected in mice co-injected with MMFOsx⁺ treated with MMP13i relative to vehicle controls, while no differences were noted in the MMFctr cohort (Fig. 5L, M). These findings indicate that Osx expression in OsteoLin-myCAFs promotes tumor growth, at least in part, by releasing MMP13. Further supporting the importance of MMP13 expression in OsteoLin-myCAFs, genetic silencing of MMP13 in MMFOsx⁺ significantly reduced their ability to support tumor growth in 3D models compared with MMFOsx⁺ expressing scramble controls (Fig. 5N–O, and Supplementary Fig. 7E).

Together, these results demonstrate that Osx expression in CAFs promotes tumor growth, at least in part, by regulating ECM remodeling and MMP13 expression, extending its role beyond serving as a marker of osteolineage-derived cells.

### OsteoLin-myCAFs are detected in human breast cancer tissues

**Fig. 6:**
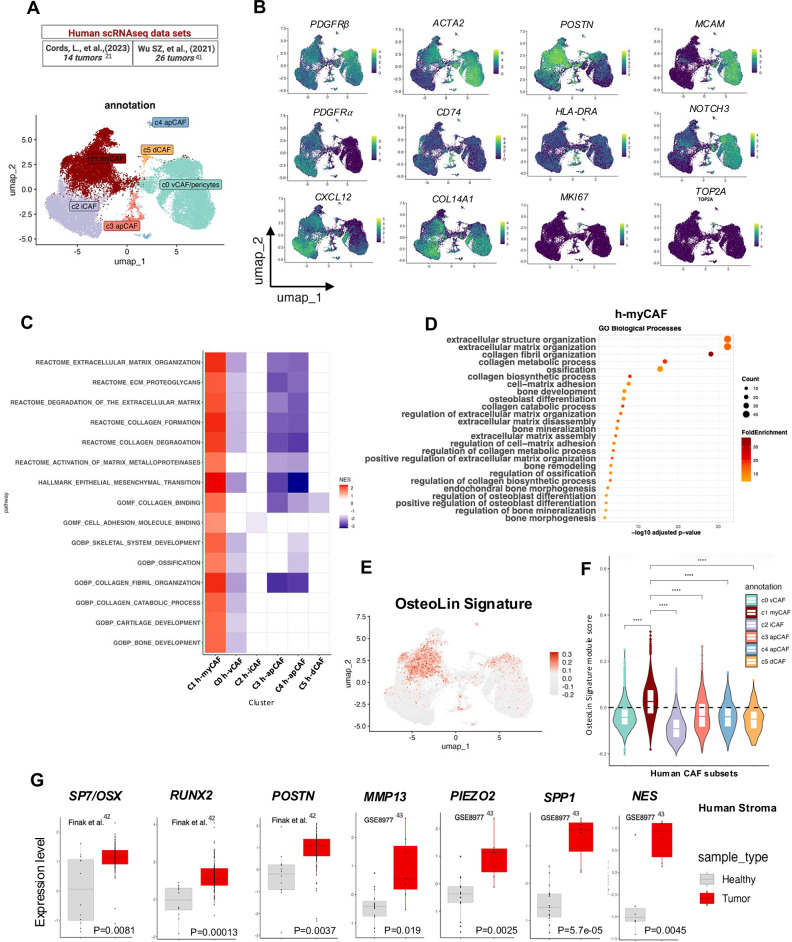
OsteoLin-myCAFs are detected in human breast cancer tissues. **A** Schematic of the two human breast cancer scRNA-seq data sets (top) used to identify h-CAF clusters by UMAP. **B** Feature plots of marker genes used to identify distinct human breast cancer CAF subsets from (**A**). **C** Heatmap of normalized enrichment scores (NES) showing enrichment of extracellular matrix-related pathways across CAF subclusters, with the highest enrichment scores in h-myCAFs. **D** Gene Ontology (GO) pathway analyses showing enrichment of pathways involved in ossification, bone development, ECM remodeling, and collagen organization in h-myCAFs. *P*-values are calculated by enrichGO using one-sided Fisher’s exact test and multiple testing correction was performed using the Benjamini-Hochberg method. **E** Feature plot showing enrichment of the murine OsteoLin-myCAF gene signature (red) across human CAF clusters, with predominant expression in the h-myCAF subcluster and minimal expression in the h-vCAF subcluster. **F** Violin plot showing expression of the murine OsteoLin-myCAF gene signature across human CAF clusters, with the highest enrichment in h-myCAFs. The lower, center, and upper bounds of each box denote the first, second, and third quartiles, respectively. Outliers are shown as colored dots. Pairwise two-sided Wilcoxon tests were performed with Bonferroni multiple testing correction to determine the statistical significance in module score differences (****P ≤ 0.0001). The numbers of cells in each annotated cluster are c0 vCAF = 10769, c1 myCAF = 8656, c2 iCAF = 6866, c3 apCAF = 686, c4 apCAF = 288, c5 dCAF = 213. **G** Human microarray dataset analyses (Finak et al. and GSE8977) showing expression levels of osteoblast-related markers in healthy versus breast tumor stroma (Finak et al: healthy *n* = 12 and tumor n = 111; GSE8977: healthy *n* = 15 and tumor *n* = 7). Unpaired two-sided Student’s T-tests were performed to determine the significance. The lower, center, and upper boundary of each box denote the first, second, and third quartile, respectively. The lower and upper whiskers extend to values no further than 1.5 * IQR from the hinges.

To test whether osteolineage-associated transcriptional programs are expressed in human BC CAFs, we analyzed scRNA-seq datasets from two independent studies. The datasets were integrated and comprised 40 TNBC, ER⁺, and HER2⁺ BC samples (Fig. 6A^21,41^). UMAP clustering identified six CAF subsets previously described in BC: vasculature-associated CAFs (C0 vCAFs; *MCAM*, *NOTCH3*), myofibroblastic CAFs (C1 myCAFs; *POSTN*, *TAGLN*), inflammatory CAFs (C2 iCAFs; *CXCL12*, *COL14A1*), antigen-presenting CAFs (C3 apCAF1 and C4 apCAF2; *CD74*, *HLA-DRA*), and dividing CAFs (C5 dCAFs; *MKI67*, *TOP2A*) (Fig. 6B; and Supplementary Data 4). Gene set enrichment and gene ontology analyses showed that h-myCAFs were preferentially enriched for pathways associated with ECM remodeling, matrix proteinases, collagen deposition, and degradation. Similar to the murine OsteoLin-myCAFs, h-myCAFs were also enriched for programs related to osteoblast differentiation, bone development, and ossification (Fig. 6C-D; Supplementary Data 4). To assess the overlap with murine OsteoLin-myCAFs, we generated an osteolineage gene signature based on the top 200 highly and uniquely expressed genes in the murine myCAF1 cluster compared with the other CAF clusters. This analysis yielded a 54-gene signature enriched for bone and ECM associated pathways, comprising the top 48 Differentially Expressed Genes (DEGs) and 6 osteoblast-related genes  (Supplementary Fig. 9A; and Supplementary Data 5). Use of this osteolineage signature in the human scRNA-seq dataset demonstrated enrichment primarily within the h-myCAF cluster, with only limited expression in a small subset of h-vCAFs (Fig. 6E). Consistent with this observation, analysis of two independent microarray datasets (Finak Dataset^42^, and GSE8977^43^) demonstrated increased expression of osteoblast-associated genes included in the osteolineage signature within tumor stroma compared with normal breast tissue. Specifically, *SP7*, *RUNX2*, *POSTN*, *SPP1*, *PIEZO2*, *NES*, and *MMP13* were significantly upregulated in the BC stroma (Fig. 6F).

Together, these results indicate that osteolineage-associated transcriptional programs identified in murine OsteoLin-myCAFs are conserved in h-myCAFs.

### Osterix expression in human myCAFs is associated with poor prognosis

**Fig. 7:**
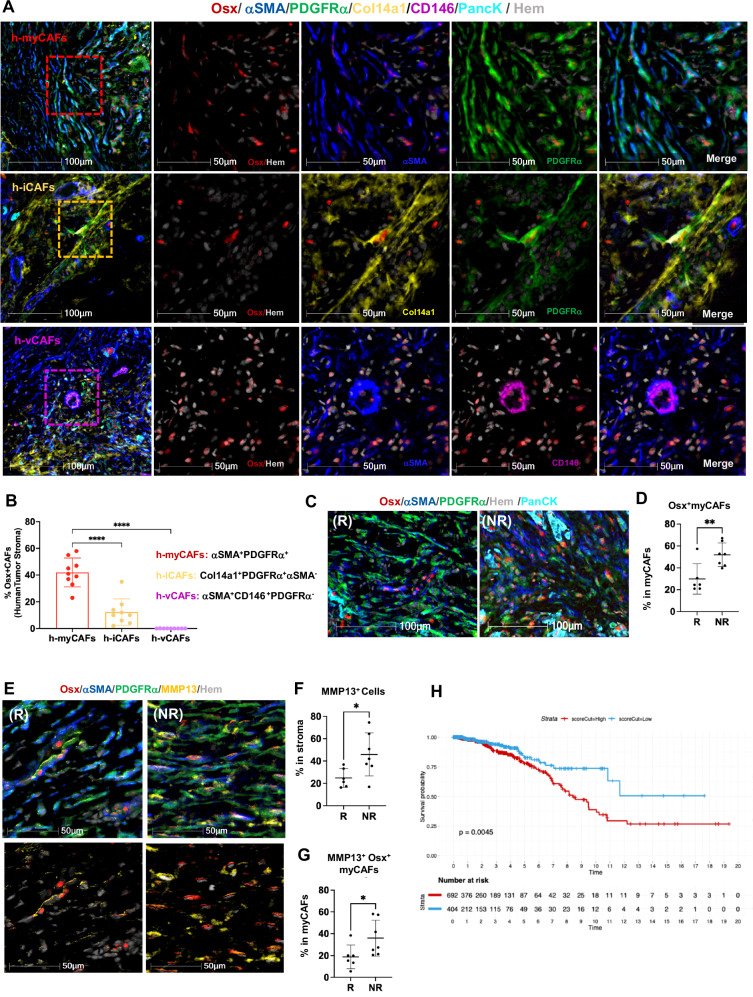
Osx is expressed in human breast cancer stroma and is associated with poor prognosis. **A** Representative mIHC images of human TNBC tissues stained for Osx (red), Hematoxylin (gray), αSMA (blue), COL14a1 (yellow), PDGFRα (green), CD146 (magenta), and panCK (cyan) (*n* = 9). **B** Quantification of Osx⁺ CAF subpopulations from tissues in (**A**), identified as h-myCAFs (αSMA^+^ PDGFRα^+^), h-iCAFs (COL14a1^+^ PDGFRα^+^ αSMA^neg^), and h-vCAFs (αSMA^+^ CD146^+^ PDGFRα^neg^) (*n* = 9). **C**–**G** Representative mIHC images of TNBC patient biopsies at diagnosis before receiving neoadjuvant chemotherapy + anti-PD1. Patients were classified as responders (R = 6) or non-responders (NR = 7), based on clinical annotations. Tissues were stained for Osx (red), Hematoxylin (gray), αSMA (blue), PDGFRα (green), MMP13 (yellow), and panCK (cyan). **D** Quantification of Osx^+^ h-myCAFs (αSMA^+^ PDGFRα^+^ cells) within the stroma (PanCK^neg^ cells) in R vs. NR. **E** Representative mIHC as in (**C**) with the addition of MMP13 antibody (yellow). **F** Quantification of MMP13^+^ cells within the stroma (PanCK^neg^ cells) in R vs. NR. **G** Quantification of MMP13^+^Osx^+^ h-myCAFs (αSMA^+^ PDGFRα^+^ cells) out of total h-myCAFs in R vs. NR. **H** Kaplan-Meier plots of overall survival for breast cancer patients segmented by high (red line) or low (blue line) abundance of orthologous OsteoLin-myCAF signature in the TCGA-BRAC database. Results are shown as mean ± SD. One-way ANOVA with Tukey’s multiple-comparison test (**B**), an unpaired two-tailed Student T-test (**D**, **F**, **H**), and an unpaired one-tailed Student T-test (**G**) were performed to determine the significance (*P ≤ 0.05, **P ≤ 0.01,***P ≤ 0.001, ****P ≤ 0.0001). Source data and exact *p* values are provided as a Source Data file.

To evaluate Osx protein expression in CAF subsets from human BC tissues, we performed mIHC using nine TNBC patient biopsies collected at the time of diagnosis, prior to any treatment. Tumor cells were identified by staining for cytokeratin (PanCK). CAF subsets were identified as previously described^19^, with h-myCAFs as αSMA⁺PDGFRα⁺, h-iCAFs as COL14A1⁺PDGFRα⁺, and h-vCAFs as αSMA⁺CD146⁺ (Fig. 7A). Quantitative analysis across samples demonstrated that Osx positivity was mainly detected in h-myCAFs, with limited expression in h-iCAFs and h-vCAFs (Fig. 7B, and Supplementary Fig. 9B).

To assess the clinical relevance of these findings, we next analyzed 13 clinically annotated TNBC biopsies collected at diagnosis, prior to starting neoadjuvant chemotherapy plus anti-PD-1, according to the Keynote522^44^. After completion of neoadjuvant therapy, patients were classified according to the Residual Cancer Burden (RCB) index. Seven patients were classified as RBC II and III with moderate to large amounts of residual tumor cells (non-responders; NR), while six were classified as RCB 0 because they achieved a pathological complete response (responders; R). Tissues were subjected to mIHC for expression of Osx and MMP13 (osteolineage markers), αSMA and PDGFRα (myCAF markers), panCK (epithelial marker), and hematoxylin (nuclear staining), followed by quantification using the Halo software (Fig. 7C; representative images). Strikingly, the proportion of h-Osx^+^myCAFs was significantly higher in the non-responders (Fig. 7D). Notably, the increase in h-Osx⁺myCAFs was also accompanied by elevated MMP13 expression (Fig.7E–G).

Finally, to evaluate whether the 54-gene OsteoLin-myCAF signature was associated with patient outcomes, we analyzed the bulk RNA-seq data from the TCGA-BRCA dataset^45^. Single-sample GSEA (ssGSEA) was used to evaluate the signature score in each tumor sample. Tumor samples were stratified into high- and low-score groups based on the higher OsteoLin-myCAFs signature score. Kaplan-Meier survival analysis revealed that tumors with higher OsteoLin-myCAF signature scores were significantly associated with reduced 10-year survival in BC patients (*p* = 0.0045; Fig. 7H). These results indicate that the presence of OsteoLin-myCAFs in patient tissues is associated with poor prognosis.

## Discussion

In this study, we identify a distinct bone-derived stromal population contributing to BC progression. We show that BM-derived Osx⁺ osteolineage cells are recruited to primary breast tumors and differentiate into a distinct subset of tumor-supportive myCAFs (OsteoLin-myCAFs) that retain an osteolineage-associated transcriptional program in both mouse models and BC patients. OsteoLin-myCAFs exhibit enhanced matrix remodeling activity, promote tumor progression, and are clinically associated with poor therapeutic response and reduced overall survival.

Osx has traditionally been viewed as a bone-restricted transcription factor^31,32^; however, Osx-expressing cells have been reported outside the skeleton. Previous lineage-tracing studies detected Osx⁺ cells infiltrating tumors. Still, these cells largely corresponded to CD45⁺ immune populations derived from rare Osx⁺ hematopoietic stem cells and lacked tumor-supportive functions^34^. Osx expression has also been observed in extraskeletal soft-tissue sarcomas and, in some cases, in tumor cells themselves^46–48^. Our work extends these observations by demonstrating that Osx^+^ BM-derived cells generate a stromal, tumor-promoting myCAF population, while barely contributing to inflammatory or vascular CAF subsets. Confirming their BM origin, we detected circulating osteolineage cells in tumor-bearing mice, and their numbers increased as tumors progressed. Similar findings have also been reported in BC patients, although expression of Osx in osteocalcin+ osteolineage cells in circulation was not evaluated^49^. While our data focuses on BC, these observations may have broader implications. Runx2, an osteogenic transcription factor highly expressed in Osx⁺ cells in bone, drives pathological fibroblast activation and ECM deposition in pulmonary fibrosis^50^. Whether such fibroblasts originate from Osx⁺ bone-derived osteolineage cells remains an open question.

Our results identify a specialized subset of myCAFs. Although OsteoLin-myCAFs represent a small fraction of the total tumor mass, they constitute a substantial proportion of the stromal compartment, accounting for ~20% of PDGFRβ⁺ cells in murine breast tumors and up to 40% of stromal populations in patient biopsies. Importantly, OsteoLin-myCAFs are molecularly distinct from previously described BM-derived CAF populations, which lack PDGFRα expression and display predominantly inflammatory features^24^. In contrast, OsteoLin-myCAFs express PDGFRα and are enriched for transcriptional programs related to ECM organization, matrix remodeling, and osteoblast differentiation in both murine and human tumors, which distinguish them from all other CAF subsets. The differences from previous BM-derived CAF subsets likely reflect heterogeneity among bone-resident progenitors and/or functional adaptation following tumor infiltration. Indeed, we find that bone-derived osteolineage cells acquire tumor-promoting properties in a context-dependent manner. OsteoLin-myCAFs from the TME markedly enhanced tumor growth in co-injection assays, while BM-derived Osx^+^ osteolineage precursors did not. These findings suggest that Osx^+^ cells in bone lack intrinsic pro-tumorigenic potential and must undergo TME-induced reprogramming to acquire CAF-like tumor-supportive properties. Another possibility, not mutually exclusive, is that a small population of OsteoLin-myCAFs already exists in the bone marrow but is too rare to matter until the tumor expands it or reprograms more cells. Consistent with tumor-driven reprogramming, bone-residing Osx⁺ cells adjacent to breast cancer bone metastases have been observed to undergo molecular and morphological changes associated with the acquisition of a CAF-like phenotype and pro-tumorigenic features (Zarrer et al., personal communication). Together, these findings support a model in which bone-derived Osx⁺ osteolineage cells retain plasticity and can undergo fibroblastic reprogramming in response to tumor-derived cues at distinct disease sites.

Our data establishes that Osx is a functional regulator of OsteoLin-myCAF’s tumor-promoting activity. Osx is well known for its role in osteoblast differentiation and has been implicated in tumor cell invasion when aberrantly expressed in cancer cells^31,47^. Still, its function within CAFs has not been previously described. In bone, Osx regulates ECM components, including collagens and matrix metalloproteinases^32,51^. Consistent with this role, OsteoLin-myCAFs were enriched in osteoblast- and skeletal-related genes, and ECM remodeling proteins. Furthermore, genetic deletion of Osx in αSMA⁺ stromal cells delayed tumor growth and reduced collagen deposition, establishing a direct functional role for Osx in regulating CAF behavior. Importantly, consistent with a functionally relevant role for OsteoLin-myCAFs in disease progression, we identified a conserved 54-gene osteolineage signature uniquely enriched in murine and human OsteoLin-myCAFs that was associated with poor survival in BC patients, underscoring the clinical relevance of this stromal population.

MMP13 emerged as a key downstream effector of Osx-driven OsteoLin-myCAF activity. In breast cancer, MMP13 has been linked to aggressive disease by promoting collagen degradation, the release of matrix-sequestered bioactive factors, and the modulation of tumor cell behavior^52,53^. Using 2D and 3D in vitro culture assays, MMP13 knockdown, and an MMP13 pharmacological inhibitor in vitro and in vivo, we demonstrate that OsteoLin-myCAFs drive tumor progression by releasing MMP13. These results are consistent with prior studies implicating stromal MMP13 in tumor invasion and progression across multiple cancer models^54–56^. Importantly, analysis of clinically annotated TNBC patient biopsies indicated that h-myCAFs expressing Osx and MMP13 were enriched in patients who failed chemotherapy plus immunotherapy.

Together, our study identifies BM-derived OsteoLin-myCAFs as a previously unrecognized source of tumor-supportive CAFs in BC. By retaining osteolineage-associated transcriptional programs and activating Osx-MMP13-dependent matrix-remodeling axis, OsteoLin-myCAFs promote tumor progression and are associated with poor clinical outcomes. These results reveal a bone-derived stromal pathway contributing to BC progression and suggest that targeting osteolineage-associated CAF programs may represent a therapeutic opportunity in patients with Osx-enriched tumor stroma.

## Methods

This study complies with all relevant ethical regulations including protocols approved by the Institutional Animal Care and Use Committee and guidelines set by the Institutional Review Board of Washington University (Protocol ID: 2025-0181) along with federal and state guidelines.

### Animals

Because the BC cell lines used in this study were obtained from female mice, we have restricted our analyses to females. Wild-type (WT) C57BL/6, FVB/N, BALB/c, B6.SJL-Ptprca Pepcb/BoyJ (CD45.1 B6, JAX #002014), *Sp7*Cre (B6.Cg-Tg(Sp7-tTAtetO-EGFP/Cre), and TdT (B6.Cg-Gt(ROSA)26SorTm9(CAG-tdTomato)Hze/J) were purchased from The Jackson Laboratory at 6-8 weeks of age. After delivery, mice were allowed to acclimatize to the new environment for at least 2 weeks. *Sp7*Cre mice, which harbor a tetracycline-responsive Osx promoter driving Cre (OsxCre), were crossed with TdT reporter mice to generate OsxCre;TdT mice. To suppress Cre expression, 200 ppm doxycycline (doxy) was added to the chow (Test Diet #1816332–203, Purina, MO, USA) and administered to specific groups of mice until weaning (P25); thereafter, pups were transitioned to standard rodent chow.

αSMACre^ERT2^ transgenic mice were generated by Dr. Ivo Kalajzic (University of Connecticut Health Center, Farmington, CT)^57^. *Osx/Sp7*^fl/fl^ mice were generated in the laboratory of Dr. Benoit de Crombrugghe (University of Texas MD Anderson Cancer Center, Houston, TX)^58^. These strains were crossed with TdT reporter mice to generate αSMACre^ERT2^;*Osx/Sp7*^fl/fl^;TdT mice. Animals were housed in a pathogen-free animal facility at Washington University (St. Louis, MO) with a 12-h light/12-h dark cycle and 20 ~ 23 °C and 40 ~ 60% humidity housing conditions.

### TNBC patient biopsies

Clinically annotated, deidentified, diagnostic biopsies from TNBC patients were obtained from the Department of Pathology at Washington University. Patients were treated with neoadjuvant chemotherapy plus immunotherapy (Keynote522^44^), consisting of pembrolizumab every 3 weeks plus paclitaxel and carboplatin weekly for the first 12 weeks, followed by evaluation of the Residual Cancer Burden (RCB) score. Responders were selected based on RCB = 0 and non-responders on RCB = II-III, with an age range between 32 and 78 years (Supplementary Table 2). Tissues from 6 responders and 7 non-responders were used for mIHC. Data and tissues were obtained in accordance with the Washington University Institutional Review Board (IRB #201105394) guidelines, and written informed consent was obtained from all patients. All patient information was deidentified before sharing it with investigators.

### Cell lines

Polyoma middle tumor-antigen murine mammary tumor cells (PyMT, C57BL/6), and mCherry-conjugated PyMT (from Dr. DeNardo, Washington University in St. Louis, MO, USA), bone-trophic GFP- and firefly-luciferase-conjugated PyMT-BO1 (from Dr Weilbaecher, Washington University in St. Louis, MO, USA), Primary mammary fibroblasts (MMF; C57BL/6) (from Dr. S.A. Stewart, Washington University in St. Louis, MO, USA), GFP- and Fluc-conjugated 4T1 murine mammary tumor cells (BALB/c; provided by David Piwnica-Worms, The University of Texas MD Anderson, Houston TX, USA), Met-1 breast cancer cell line (FVB/N) and immortalized CAFs (FVB/N; provided by Dr. D. Longmore, Washington University in St. Louis, MO, USA) were cultured at 37 °C with 5% CO_2_ in complete Dulbecco's modified Eagle' medium (DMEM) supplemented with 10% heat-inactivated FBS, 100 μg/ml streptomycin, 100 IU/ml penicillin, and 1 mM sodium pyruvate. All cell lines were tested for *Mycoplasma* every 2 months. Aliquots for each cell line were used for 1 month after thawing.

### Lentivirus preparation and cell infection

The puromycin-resistant pLenti-puro ORF clone of Sp7 (mGFP-tagged Origene MR226379L4) was used to ectopically express *Sp7* (*Osx*) in MMFs or immortalized CAFs. pLenti-puro (Addgene plasmid #39481) was used as a vehicle control. Briefly, HEK293T cells were transiently transfected with lentiviral accessory plasmids (VSV.G and psPAX2) and the designated transfer plasmid using the TransIT-293 transfection reagent (MIR2700, Mirus Bio LLC), according to the manufacturer’s instructions. The virus-containing cell supernatant was collected 48 h after transfection, filtered through a 0.45-μm filter, supplemented with 8 mg/mL protamine sulfate, and immediately added to target cells. Selection of cells expressing vehicle or Osx was performed in DMEM containing 2μg/ml puromycin and supplemented with 10% heat-inactivated fetal bovine serum, 100 IU/ml penicillin plus 100 μg/ml streptomycin, and 1 mM sodium pyruvate.

### siRNA-mediated MMP13 knockdown

siRNA targeting MMP13 was used to silence  *Mmp13*, and non-targeting siRNA was used as a negative control (Dharmacon, *Mmp13* smart pool and non-targeting pool control, on-Targetplus siRNA). MMFOsx^+^ were transiently transfected with siRNA in OptiMEM medium (Gibco, 31985-070) with Lipofectamine RNAiMAX (Invitrogen, 13778075). After 24 h, the medium was replaced, and the cells were seeded for further experiments. The efficacy of silencing was analyzed using qRT-PCR after transfection using the following primers:

m*Mmp13*: 5’-CCCAGGAGCCCTGATGTTTC-3’ and 5’-GCGCCAGAAGAATCTGTCTTT-3’

*mGapdh* 5’-TGCACCACCAACTGCTTAG-3’5’-GGATGCAGGGATGATGTTC-3’.

### Tumor Cell Inoculation and CAF-tumor co-injections

Tumor cells (1 × 10⁵) resuspended in a 1:1 PBS/Matrigel mixture (Corning, 354234; 50 μL) were implanted orthotopically into the mammary fat pad (MFP) of 6–8-week-old female mice.

For CAF co-injection experiments, tumor cells were mixed with stromal populations at defined stroma-to-tumor ratios before implantation. Experiments co-injecting tumor-derived or BM-derived OsxTdT^+^ cells, MMFOsx⁺ and MMFctr, or CAFOsx+ and CAFctr, were performed at a 1:1 stroma-to-tumor ratio. For direct comparison of CAF subsets, PyMT-BO1-GFP⁺ tumor cells were co-injected with OsxTdT^+^ or OsxTdT^neg^ at a 1:2 stroma-to-tumor ratio. Tumor cell-only injections were used as controls for all co-injection experiments.

For inducible Osx deletion studies, Cre recombination was activated in 12-week-old αSMA-OsxcKOTdT and αSMA-OsxWTTdT mice by intraperitoneal tamoxifen administration (100 mg/kg daily for five consecutive days), beginning 4 h before tumor implantation in MFP.

Tumor growth for all experiments was monitored with caliper measurements every other day starting 7 days after implantation, and tumor volume was calculated using the formula V = 0.5 × (length × width²). The maximal tumor size ( < 2000 mm^3^) permitted by the Institutional Animal Care and Use Committee was not exceeded.

### Tumor, bone marrow, and blood processing

Tumors were dissociated in serum-free medium containing collagenase type I (2 mg/mL, Thermo Scientific T-27491) and DNase I (2 U/mL, Sigma-Aldrich D5025-150KU) for 30 min at 37 °C. The resulting cell suspension was filtered through 70-µm nylon strainers and washed twice with PBS. Red blood cells were lysed using RBC lysis buffer (Sigma-Aldrich R7757), followed by an additional wash in PBS. Cells were counted and prepared for subsequent experiments.

BM cells were isolated from mouse femurs and tibias. Hindlimbs were dissected, cleaned of muscle tissue, and bones were maintained on ice in PBS supplemented with 2% FBS. BM-derived OsxTdT^+^ populations were isolated from femurs, tibias, and hips. The ends of each bone were trimmed to expose the marrow cavity, and BM was isolated by centrifugation in cold PBS at 13,800 g for 2 minutes. Following RBC lysis (Sigma-Aldrich) and washing, cells were counted and prepared for subsequent experiments.

Peripheral blood was collected by cardiac puncture. Red blood cells were removed by two sequential 10-minute incubations with RBC lysis buffer, followed by an additional wash in PBS. The remaining cells were processed for flow cytometric staining.

### CAF isolation protocol

For CAF isolation, tumors were minced and enzymatically digested to generate single-cell suspensions. Cell suspensions from each tumor were plated onto 100-mm tissue culture dishes (one tumor per dish) in DMEM/F12 supplemented with 10% FBS and incubated for 30 minutes to allow preferential attachment of stromal cells. Non-adherent tumor and immune cells were removed by gentle washing, and adherent stromal cells were subsequently cultured in DMEM/F12 containing 10% FBS, 100 IU/ml penicillin plus 100 μg/ml streptomycin, 1 mM sodium pyruvate, and 1% L-glutamine for 16 h to increase stromal yield. Cells were subsequently harvested and used for flow cytometry and FACS sorting for characterization and isolation of OsxTdT⁺ and OsxTdT^neg^ CAF populations.

### BM OsxTdT^+^ cell isolation

BM-derived OsxTdT^+^ populations were isolated from femurs, tibias, and hips of OsxCre;TdT^+^ mice, as described above. BM single-cell suspensions were subjected to red blood cell lysis followed by depletion of CD45⁺ hematopoietic cells using magnetic bead separation according to the manufacturer’s instructions (Miltenyi Biotec, 130-052-301). The remaining CD45^neg^ stromal fraction was used for antibody staining and FACS sorting of OsxTdT^+^ cells.

### Administration of the MMP13 inhibitor

MMP13 inhibitor CL-82198 (0.5 mg/mouse; MedChem Express) dissolved in 10% DMSO, 40% PEG300, and 50% PBS, was administered by intravenous injection for 10 consecutive days to tumor-bearing mice. Control mice received vehicle (10% DMSO, 40% PEG300, 50% PBS) following the same route and schedule of administration as the MMP13 inhibitor.

### BM transplantation

Recipient C57BL/6 CD45.1 mice received two doses of 400 cGy 4 h apart, using an X-ray irradiator (XRAD 320), followed by transplantation of BM cells collected from at least two CD45.2 OsxCre⁺;TdT⁺ donor mice. 200 μL containing 5 × 10⁶ BM cells were administered via retro-orbital injection into each CD45.1 recipient mouse. Mice were given sucrose water for a week and monitored for the following 2 weeks for signs of radiation sickness or weight loss. Six weeks after irradiation, mice were used for the indicated experiments.

### Multiparametric flow cytometry

For all tissues, single-cell suspensions were blocked with anti-mouse CD16/CD32 Fc receptor blocking antibody before staining. Cells were stained in FACS buffer consisting of PBS supplemented with 0.5% BSA, 2 mM EDTA, and 0.01% sodium azide, using fluorochrome-conjugated anti-mouse antibodies (Supplementary Table 1).

Flow cytometric acquisition was performed using a BD LSR Fortessa X-20 cell analyzer with FACSDiva software (BD Biosciences). Data were analyzed using FlowJo software (v10.9.0, Tree Star).

### Immunofluorescence staining and confocal imaging

Tumors were fixed in 4% paraformaldehyde (PFA) at 4 °C overnight. Fixed specimens were then dehydrated in 30% sucrose solution and cut into 50 μm-thick sections at the cryostat (Leica). Staining on free-floating sections was performed. Cryosections were blocked for 4 h in 5% BSA solution with 0.5% Triton X-100 and stained with primary antibodies for 48 h at 4 °C. Secondary staining was performed at room temperature for 2 h (Antibodies listed in Supplementary Table 1). Sections were then mounted on Superfrost glass slides (Fisher Scientific) and embedded in Prolong Glass antifade mounting media (Thermo Fisher Scientific). Sections were covered with 1.5H high-precision cover glass (Marienfeld Superior) and allowed to dry overnight at room temperature before imaging. Confocal imaging of tumor cryosections was performed using a Zeiss LSM880 Airyscan inverted confocal microscope with a 40X/NA 1 oil-immersion objective. Images were acquired at 2048 × 2048‑pixel resolution with 10–30 μm‑thick z‑stacks (z‑step = 1 μm, line averaging = 2) using ZEN Black software (ZEISS Efficient Navigation, Zeiss). Maximum‑intensity projections were generated in ImageJ (version 2.3.0).

### Histology and Immunohistochemistry

Freshly isolated mouse primary tumors were fixed in 10% neutral-buffered formalin (DiRuscio & Associates, Inc.) for 24 h. Tissues were paraffin-embedded and sectioned 5 μm thick by the histology core of the Washington University Musculoskeletal Research Center. For human studies, sections of diagnostic biopsies were obtained from the Department of Pathology at Washington University.

Tissues were stained (antibodies listed in Supplementary Table 1) using the Bond Rxm (Leica Biosystems) after dewaxing and appropriate epitope retrieval. Immunostaining was chromogenically visualized using the Bond Polymer Refine Detection (#DS9800, Leica Biosystems) or the Bond Polymer Refine Red Detection (#DS9390, Leica Biosystems). Slides were mounted using Xylene-based Cytoseal (Thermo Fisher) or Vectamount (Vector Labs), as appropriate, scanned with a Zeiss AxioScan 7 microscope, and IHC analyses performed using the HALO image analysis platform (Indica Labs; Deconvolution v1.1.1, Multiplex IHC v.3.2.3 algorithms).

### SHG imaging of collagen

Collagen fiber organization was assessed by Second-Harmonic Generation (SHG) microscopy of 20 µm frozen tumor sections. SHG images were acquired using a Zeiss LSM 880 laser-scanning microscope equipped with a Plan-Apochromat 20×/0.8 M27 objective under two-photon excitation. Laser intensity, detector gain, and exposure time were kept constant for all acquisitions within each experiment to allow quantitative comparison across samples. Collagen enrichment was quantified as the total collagen-covered area using Imaris software (Bitplane, Oxford Instruments V 10.1.1), applying identical segmentation and thresholding parameters to all images.

### Picrosirius Red staining

Collagen deposition in 5 µm paraffin-embedded tumor sections was evaluated using Picrosirius Red staining (Abcam, Picrosirius Red Stain Kit, #ab150681). Slides were baked at 60°C for 60 min before deparaffinization and staining, according to the manufacturer’s instructions. Stained sections were scanned with a Zeiss AxioScan.Z7 slide scanner, and collagen-positive areas and intensity were quantified from whole-slide images using ImageJ (version 2.3.0) with a consistent thresholding parameter across all samples.

### Generation of MMF-derived conditioned media and MTT assays

MMFctr or MMFOsx⁺ cells were cultured in Dulbecco’s Modified Eagle Medium (DMEM) supplemented with 10% FBS, 100 IU/ml penicillin plus 100 μg/ml streptomycin, and 1 mM sodium pyruvate. Once cells reached ~90% confluence, they were washed with PBS and incubated in 1% FBS medium. Conditioned media (CM) was collected after 24 hours. Tumor cells were seeded in 96-well plates (5 × 10³ cells/180 μl per well) and treated with 100% CM from MMFctr or MMFOsx^+^. Tumor cells cultured in 1% FBS media were used as a control. After 48 hours, cell viability was assessed using the MTT assay (Invitrogen) according to the manufacturer’s protocol. In some experiments, tumor cells were cultured for 72 hours with or without MMFOsx⁺ CM, in the presence or absence of MMP13i (20 µM). MTT assay was also performed on tumor cells or MMFOsx^+^ alone cultured in DMEM supplemented with 10% FBS, 100 IU/ml penicillin plus 100 μg/ml streptomycin, and 1 mM sodium pyruvate in the presence or absence of MMP13i.

### Crystal violet assay

Tumor cells cultured for 48–72 h at 37 °C in the presence of MMFctr or MMFOsx^+^ CM and treated with vehicle or 20 µM MMP13i (details described in MTT assay), were washed once with 1X PBS and fixed with cold methanol for 10 minutes at room temperature. The fixed cells were washed twice with 1X PBS to remove residual methanol, then stained with a crystal violet solution (1% crystal violet in 25% methanol in distilled water) for 10 minutes. Excess stain was removed by thoroughly rinsing the wells with distilled water. After air drying, stained cells were visualized under a microscope, and the covered area was calculated using ImageJ (Version 2.3.0).

### 3D Spheroid cultures

Three-dimensional spheroid cultures were established using Cultrex Organoid Qualified BME, Type 2 (R&D Systems, Inc., 3532-005-02). A total of 2.5 × 10⁴ mCherry⁺PyMT cells, either alone or combined with MMFctr or MMFOsx⁺ at a 1:1 ratio, were resuspended in 20 µl of Matrigel. Domes were plated into a 6-well plate (three domes per well, per condition). The plate was placed in a tissue culture incubator at 37°C with 5% CO₂ to allow the organoids to polymerize for 20 minutes. After polymerization, 2 mL of medium (DMEM supplemented with 10% FBS, 100 IU/ml penicillin plus 100 μg/ml streptomycin, and 1 mM sodium pyruvate) was added to each well. In some experiments, 20 µM MMP13i was added to the media every two days with a half media change, and the experiment was stopped on day 5. At least *n* = 3 per group was used for the statistical power analysis. mCherry^+^PyMT tumor cell growth was monitored by fluorescence imaging using a Nikon Eclipse Ti2 (temperature 37 °C, 5% CO_2_, and a humid environment) and a 4X objective stitch to image the entire dome. Images were captured every 2 days using an ORCA-Flash4.0 LT3 Digital CMOS camera and processed via NIS-Elements (version 5.42.04) 64-bit software. Z-stacks were obtained to reconstruct the entire dome. Images were analyzed using NIS-Elements software to quantify fluorescence intensity and assess the number of clusters, while ImageJ (version 2.3.0) was used to measure the surface area of cell clusters.

In MMP13 knockdown experiments, PyMTmCherry^+^ cells were mixed 1:1 with MMFOsx⁺ cells transiently transfected with MMP13 siRNA or non-targeting control siRNA and resuspended in 20 µl of Matrigel. Domes were prepared as described above, and cells were fixed in 3.7% Formalin for 3 h. Tumor cell growth was monitored using a Leica SP8 confocal microscope using a 10x objective HC PL APO CS2 10x/0,4 DRY, 7 × 7 tiles, Line average 1. Images were analyzed using ImageJ (version 1.54 d).

### Quantitative Real-time PCR analysis

Total RNA was extracted using TRIzol reagent (Invitrogen, CA, USA) and purified with the RNeasy Mini Kit (Qiagen, Cat. No. 74104) according to the manufacturer’s instructions. RNA concentration was determined using an ND-1000 spectrophotometer (NanoDrop Technologies). The cDNA was synthesized with 1 μg RNA using High-Capacity cDNA Reverse Transcription Kit (#4368814, Applied Biosystems, CA, USA). The amount of each gene was determined using Power SYBR Green mix on the 7300 Real-Time PCR System (Applied Biosystems). Cyclophilin mRNA was used as a housekeeping control. Specific primers for mice were as follows: *Cyclophilin*, 5’-AGCATACAGGTCCTGGCATC-3’ and 5’-TTCACCTTCCCAAAGACCAC-3’; *Osx/Sp7*, 5’-AAGGGTGGGTAGTCATTTGCA-3’ and 5’-CCCTTCTCAAGCACCAATGG-3’; *Mmp13*, 5’-ACAGGGGCTAAGGCAGAAA-3’ and 5’-CGCTAAGGAAAGCAGAGAGG-3’. Relative gene expression quantification was determined using the 2^-ΔΔCt^ method.

### Single-cell RNA sequencing

Total GFP^neg^CD45^neg^ stromal fraction was sorted from 3 PyMT-BO1-GFP^+^ orthotopic tumors each, eleven days post-inoculation in OsxCre⁺;TdT⁺ mice. Sorted cells were pooled and resuspended in PBS with 0.04% BSA at a final concentration of ~1000 cells/μl. In parallel, GFP^neg^CD45^neg^TdT^+^ were sorted from 3 additional tumors in OsxCre⁺;TdT⁺ mice, pooled and resuspended as described above. cDNA was prepared after the GEM generation and barcoding, followed by the GEM-RT reaction and bead cleanup. Purified cDNA was amplified for 11–13 cycles, then cleaned up using SPRIselect beads. The sample’s cDNA concentration was determined using a Bioanalyzer. GEX libraries were prepared as recommended by the 10x Genomics Chromium Single Cell 3’ Reagent Kits User Guide (v3.1 Chemistry Dual Index) with appropriate modifications to the PCR cycles based on the calculated cDNA concentration. For sample preparation on the 10x Genomics platform, the Chromium Next GEM Single Cell 3’ Kit v3.1, 16 rxns (PN-1000268), Chromium Next GEM Chip G Single Cell Kit, 48 rxns (PN-1000120), and Dual Index Kit TT Set A, 96 rxns (PN-1000215) were used. The concentration of each library was determined by qPCR using the KAPA Library Quantification Kit (KAPA Biosystems/Roche) according to the manufacturer’s protocol to produce cluster counts suitable for the Illumina NovaSeq 6000 instrument. Normalized libraries were sequenced on a NovaSeq 6000 S4 Flow Cell using the XP workflow and a 50 × 10 × 16 × 150 sequencing recipe, according to the manufacturer’s protocol. A median sequencing depth of 50,000 reads/cell was targeted for each Gene Expression Library.

### Mouse single-cell RNA-sequencing analysis

FASTQ files were aligned to a customized GRCm39 reference genome built with cellranger (v7.0.0) mkref per 10X’s tutorial to include *Eyfp* and *tdTomato* (including 3’UTR). Cellranger count with default parameters was used for sequence alignment and generation of the count matrix.

Analysis was performed using Seurat v4.1.1. For quality control, cells with mitochondrial content (percent.mt) exceeding 6% or containing <3500 or > 9500 genes (nFeature_RNA) were removed. After removing unwanted cells and genes, counts were normalized and scaled via the *SCTransform* function^59^. Potential batch effects were removed using harmony^60^. Top 20 principal components (PCs) and resolutions of 0.3 (for the entire dataset) or 0.35 (for CAF/pericytes) were used for downstream analysis. After preliminary annotation, CAFs were isolated for further analysis using the same workflow. Differential expression analysis was performed using the FindAllMarkers function with the Wilcoxon test. GO enrichment analysis was performed using the enrichGO function from clusterProfiler^61^. Top DEGs were selected based on average log2fold change weighted by the proportion of expressing cells within each cluster (avg_log2FC * pct. 1). Murine OsteoLin-myCAFs were defined according to the proportion of OsxTdT⁺ cells, and an osteolineage gene signature was generated consisting of 54 genes. The signature included 48 of the top 50 DEGs ranked by log₂ fold change weighted by differential expression between in-cluster and out-of-cluster cells [avg_log2FC * (pct.1 − pct.2)]. Two murine genes lacked identifiable human orthologs and were excluded. The signature additionally included six osteoblast-related genes identified from Gene Ontology (GO) enrichment analysis of the top 200 DEGs.

### Human scRNA-seq analysis

Processed data were retrieved from E-MTAB-10607 and GSE176078^21,41^. Data was processed using Seurat v5.2.1. Cells with percent.mt > 10%, log10 nCount_RNA < 2 or > 5, and log10 nFeature_RNA < 2 or > 4 were removed. After log-normalization and MVP-based feature selection, the data were scaled by regressing out percent.mt and nCount_RNA. Sample-wise batch effect was regressed out using harmony^60^. Top 40 PCs and resolutions between 0.1 and 1 were used for downstream analysis. The CAF clusters were isolated and merged from both datasets for further analysis using the same workflow with additional correction for dataset differences. DEG and GO analyses were performed similarly to the mouse data. The OsteoLin-myCAF signature score was calculated for each cell using the AddModuleScore function from Seurat.

### Survival analyses

The TCGA-BRCA bulk RNA-seq data^62^ were retrieved and normalized before performing ssGSEA-2.0 (https://github.com/broadinstitute/ssGSEA2.0). Patients were divided into two groups using a score of 4, a value around the median of the score distribution, as the threshold for high enrichment of the OsteoLin-myCAF signature. Survival analysis was performed using the K-M curve from the RTCGA package.

### Statistics and reproducibility

All statistical analyses were performed using GraphPad Prism for Mac (v11) except for scRNA-seq datasets. Comparisons between two groups were performed using unpaired, two-tailed t-tests (unless otherwise specified). For multiple-group comparisons, one-way ANOVA was used; experiments involving multiple groups across time were analyzed using two-way ANOVA. When significant effects were observed, Tukey’s or Sidak’s multiple-comparisons post hoc tests were applied, as specified in the figure legends. Data are presented as mean ± SD or mean ± SEM, as indicated in the figure legends. A *p*-value equal or less than 0.05 was considered statistically significant. In vitro experiments were performed in technical and biological triplicates. In vivo studies were conducted in at least 3 independent experiments, with sample sizes indicated in the corresponding figure legends.

### Reporting summary

Further information on research design is available in the Nature Portfolio Reporting Summary linked to this article.

## Supplementary information

Supplementary Information

Description of Additional Supplementary Files

Supplementary Data 1

Supplementary Data 2

Supplementary Data 3

Supplementary Data 4

Supplementary Data 5

Reporting Summary

Transparent Peer Review file

## Source data

Source Data

## Data Availability

The single-cell RNA sequencing data generated in this study were deposited at Gene Expression Omnibus under GSE292888. The processed feature barcode matrices are included in the same accession. The processed human scRNA-seq data were retrieved from https://zenodo.org/records/7540604 (10.5281/zenodo.7540604) and GSE176078 as specified in the text. Public human microarray data were retrieved from GSE8977 and GSE9014 using getGEO in R. The TCGA-BRAC data bulk RNA-seq was retrieved using the TCGAbiolinks function in R (see codes” Human-2-0_prepare-tcga-brca-data.R”) and the clinical metadata were retrieved from the supplementary tables of the TCGA-BRAC 27653561^62^ and the survivalTCGA function. Raw flow cytometry data and deconvoluted mIHC images from patients’ biopsies are available at (10.5281/zenodo.20128707). The remaining data are available within the Article, Supplementary Information, and Source Data file. Source data are provided with this paper.

## Code availability

Codes for the tdTomato sequence used for creating the customized reference genome are available at https://zenodo.org/records/19655602^63^ and documented in the Zenodo page. Codes and relevant information for data analysis and visualization in R are available at (10.5281/zenodo.20097508).
