# Supplementary material for: Bone-derived Osterix+ osteolineage cells are a source of tumor-promoting myofibroblastic cancer-associated fibroblasts in breast cancer
Source: Nat Commun. 2026 Jun 11;17:7452. doi: 10.1038/s41467-026-73980-7 (PMC13408961; doi:10.1038/s41467-026-73980-7)
Supplement: Supplementary file 1 — Supplementary Information [file 41467_2026_73980_MOESM1_ESM.pdf]

Supplementary Figure 1

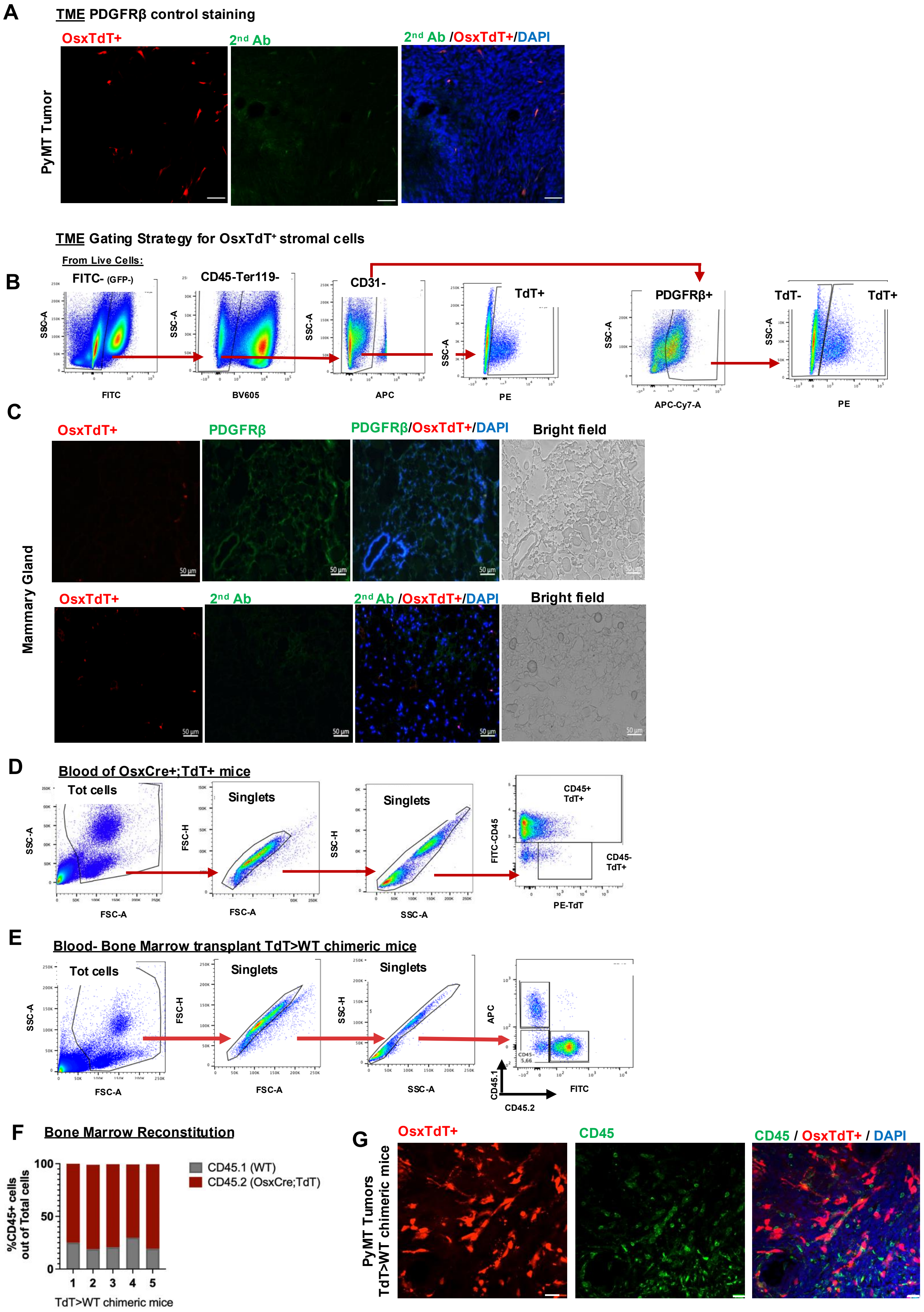

A, Representative Immunofluorescence (IF) images of the tumor microenvironment (TME) showing secondary antibody-only control staining (Alexa Fluor 488/FITC, green) with OsxTdT$^+$ cells (red) and nuclei (DAPI, blue). Scale bar 50 μM, n=3 . **B,** Flow cytometry gating strategy used to determine the percentage of OsxTdT$^+$ cells and PDGFRβ$^+$ OsxTdT$^+$ stromal cells in the TME in Figure 1C-E. n=5. **C,** Representative IF images and bright field of healthy mammary gland showing PDGFRβ (green), OsxTdT$^+$ cells (red), and nuclei (DAPI, blue) (top). Secondary antibody-only control shown below (Scale bar 50 μM, n=3). **D,** Flow cytometry gating strategy used to determine the percentage of circulating OsxTdT$^+$ cells in Figure 1F. n=3. **E, F** Flow cytometry gating strategy for circulating OsxTdT$^+$ cells and quantification of blood chimerism based on CD45.1 and CD45.2 expression in CD45.1 WT mice transplanted with bone marrow from OsxCre$^+$;TdT$^+$ animals (TdT>WT chimeric mice, n=3/group). **G,** Representative IF images showing CD45 staining (green), OsxTdT$^+$ cells (red), and nuclei (DAPI, blue) in PyMT tumors from TdT>WT chimeric mice (Scale bar 50 μM, n=3).

Supplementary Figure 2

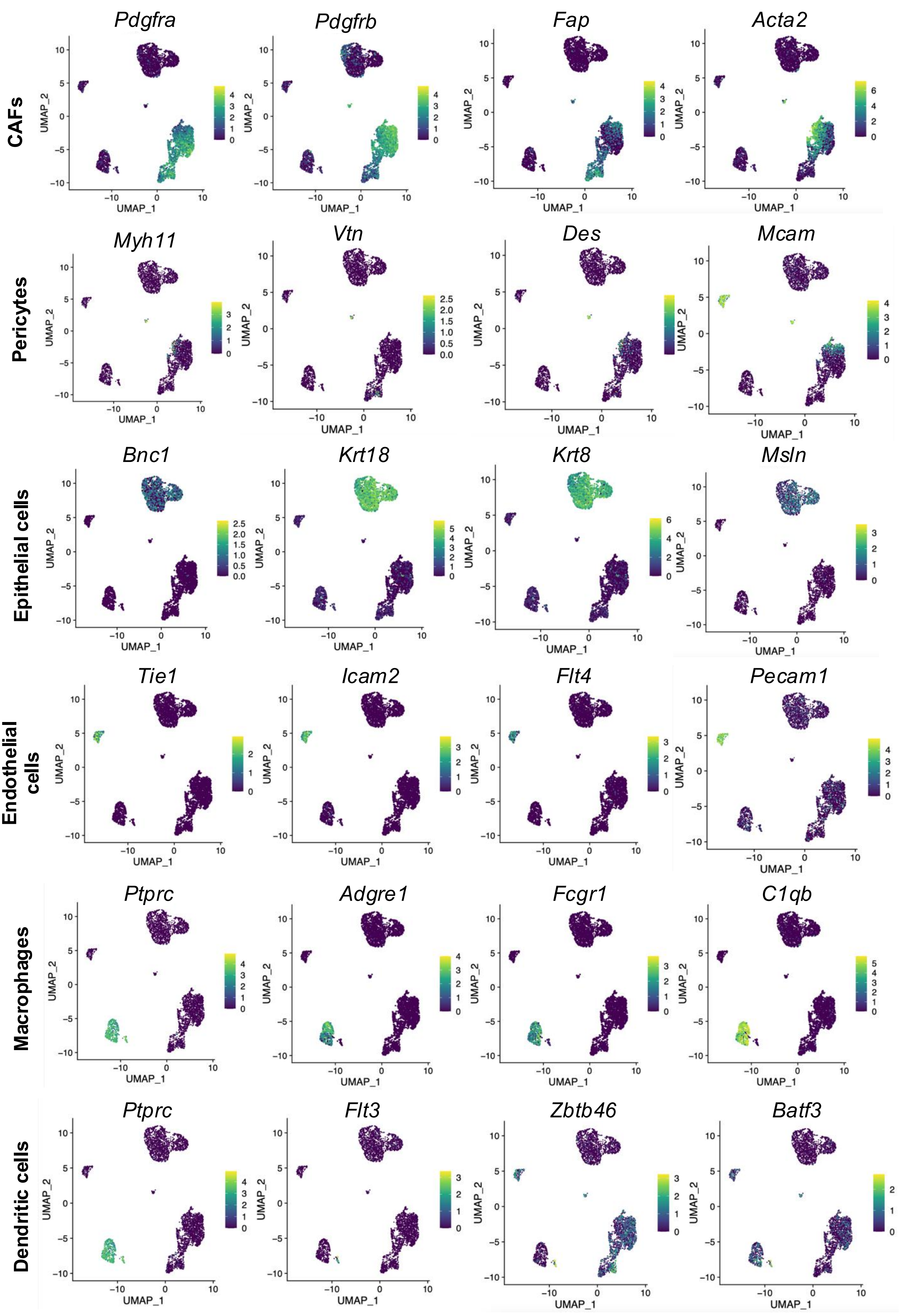

Feature plots showing expression of subpopulation markers in murine scRNAseq data set (n=6).

Supplementary Figure 3

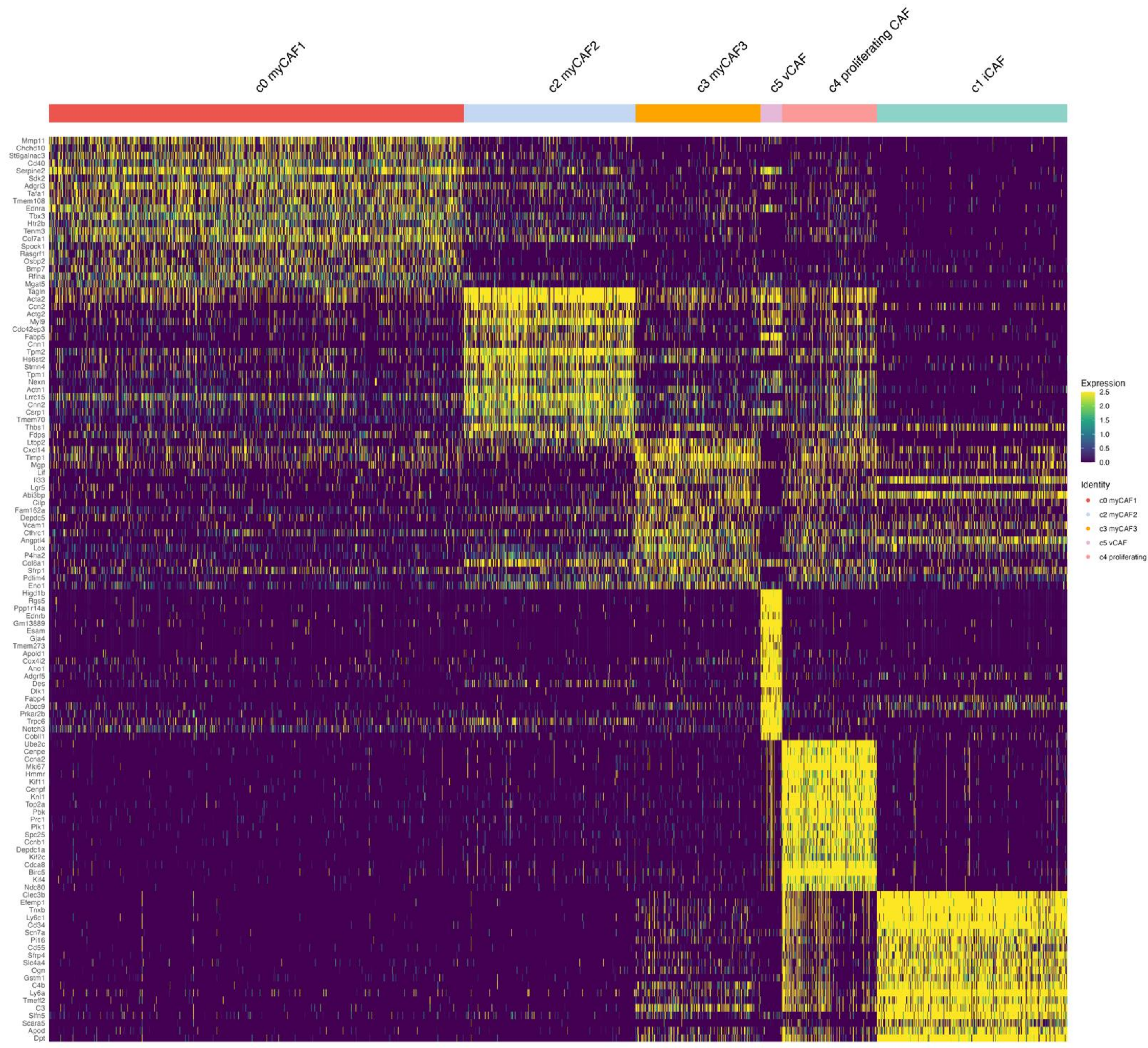

Heatmap of top genes among CAF subclusters in murine scRNAseq dataset (n=6).

Supplementary Figure 4

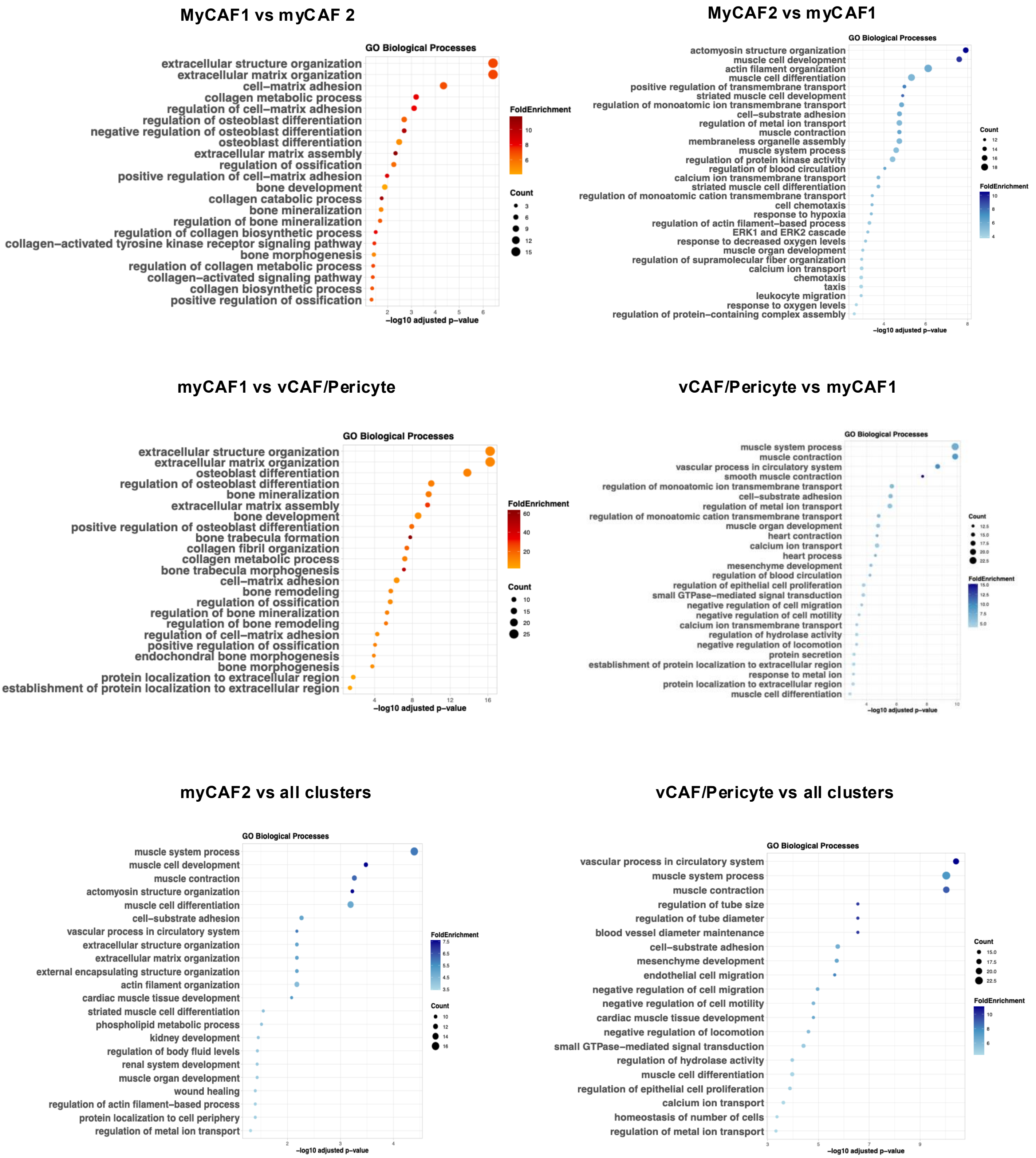

Gene ontology (GO) Pathway Analysis of differentially expressed genes (DEGs) in CAF subclusters from murine scRNA-seq dataset. P-values were calculated using enrichGO with one-sided Fisher’s exact test, and multiple testing correction was performed using the Benjamini-Hochberg method. Differential expression for each paired comparison was performed using FindAllMarkers with default parameters. Top GO pathways were selected based on gene counts (n=6).

Supplementary Figure 5

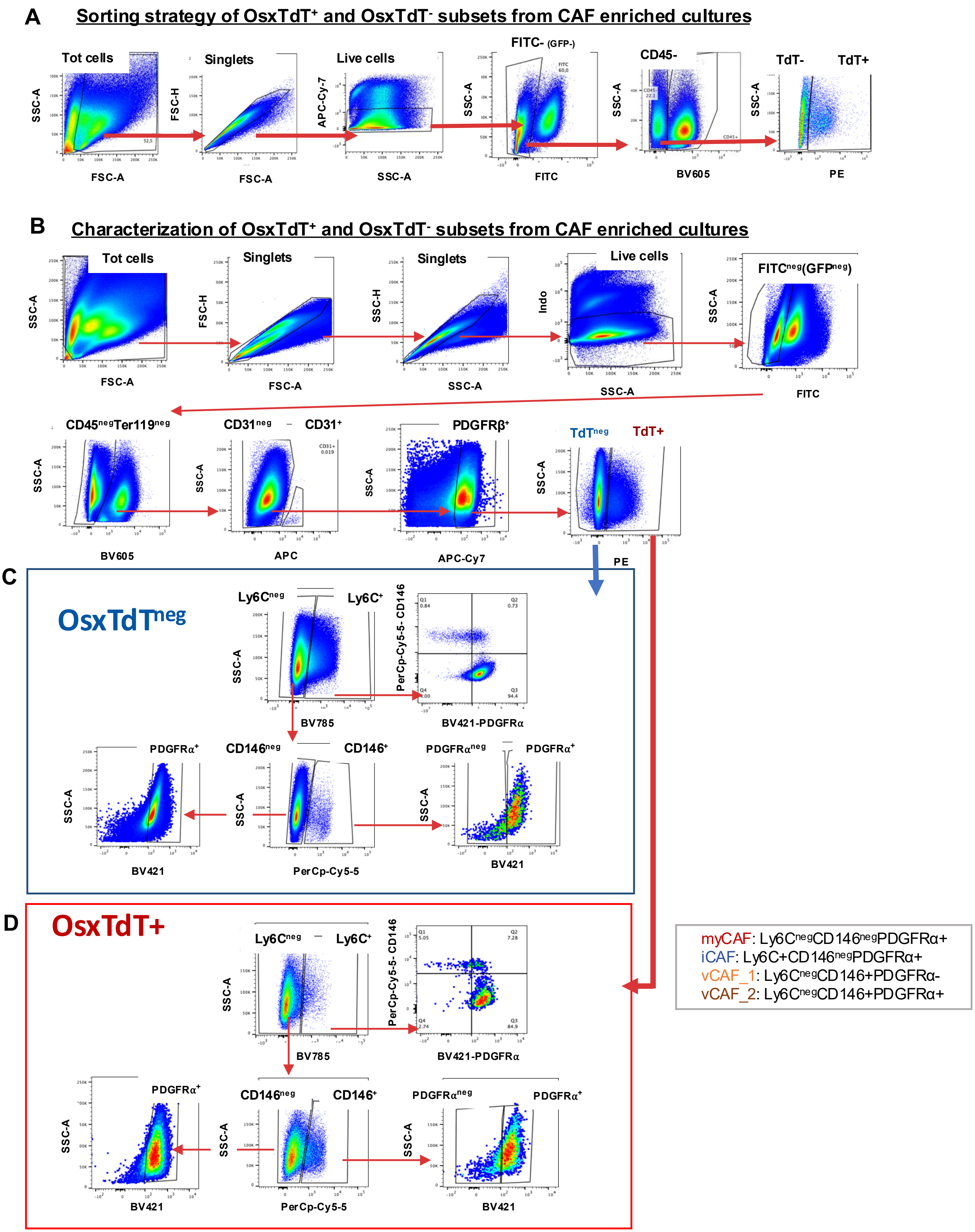

A, Gating strategy used for sorting OsxTdT^{+} and OsxTdT^{neg} subsets from CAF enriched cultures in Figure 3C (n=4). **B-D,** Gating strategy for flow cytometric characterization of OsxTdT^{+} and OsxTdT^{neg} populations from CAF-enriched cultures from PyMT-BO1-GFP^{+} tumors in OsxCre^{+};TdT $^{+}$ mice **(B)** shown in Figure 5B (n=3). CAFs were defined after exclusion of tumor cells (GFP^{+}) and immune/endothelial lineage populations (CD45^{+}, Ter119^{+}, CD31^{+}), followed by selection of PDGFRβ^{+} stromal cells and separation into OsxTdT^{+} **(C)** and OsxTdT^{neg} **(D)** subsets.

Supplementary Figure 6

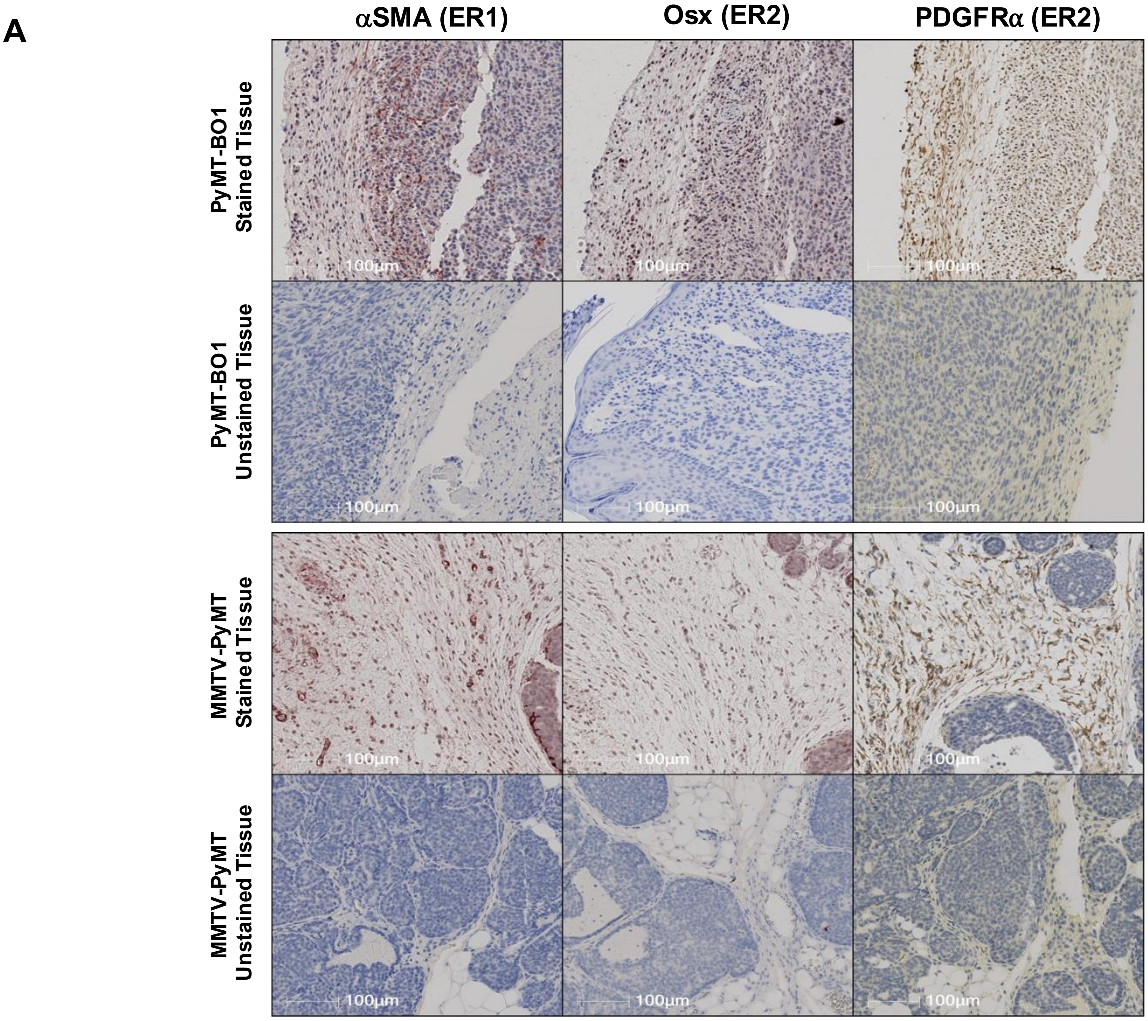

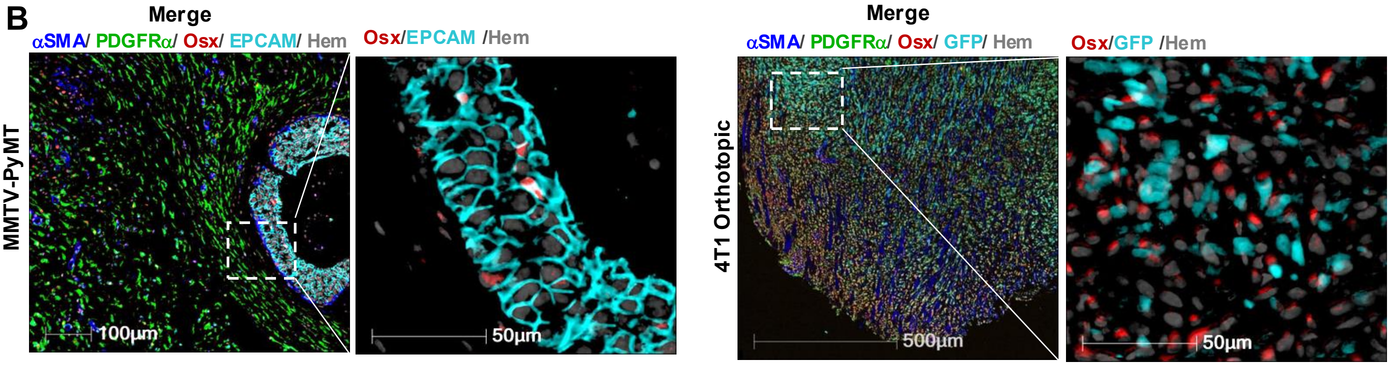

A, Representative images of PyMT-BO1 and MMTV-PyMT tumor sections subjected to antigen retrieval with BOND Epitope Retrieval Solution 1 (ER1) or BOND Epitope Retrieval Solution 2 (ER2) in the absence of primary antibody, and alongside sections processed with the full BOND mIHC protocol with indicated primary antibodies (n=3). **B,** Representative deconvoluted mIHC images of spontaneous MMTV-PyMT, and 4T1 breast tumors stained for Osx (red), hematoxylin (gray), αSMA (blue), PDGFRα (green), and EPCAM or GFP (cyan) (n=3). Staining of the corresponding stromal compartment shown in Figure 3E.

Supplementary Figure 7

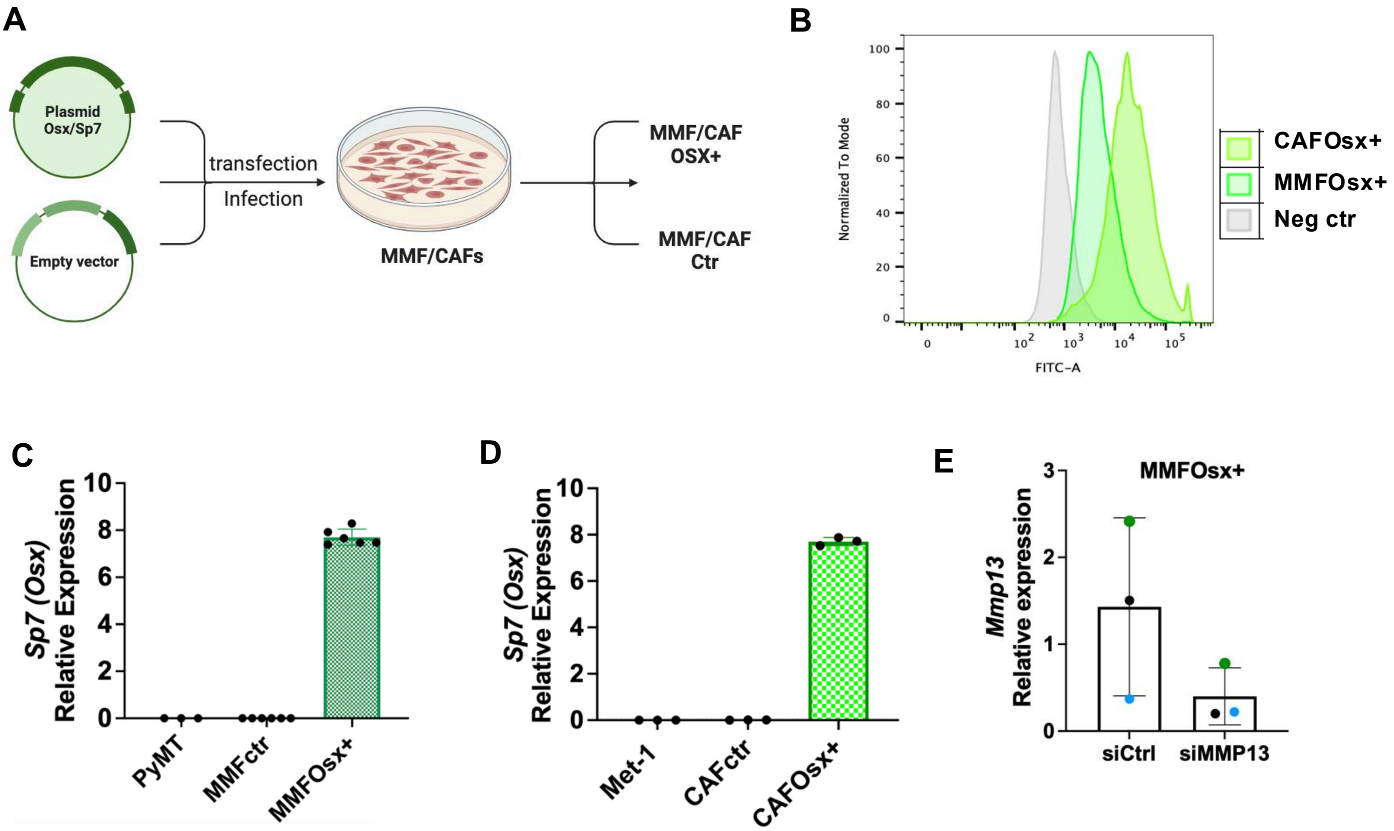

A, Schematic representation of GFP-tagged Osx/SP7 plasmid or the corresponding empty vector used to generate MMFCtr, MMFOsx⁺, CAFctr, and CAFOsx⁺ for experiments in Figure 4A-G. **B,** Infection and transfection efficiency determined by flow cytometry through detection of GFP expression in indicated cells (n=3). **C-D,** *Sp7* expression validated by qPCR in MMFOsx⁺ (n=6) and CAFOsx⁺ (n=3) . Tumor cells (n=3), along with MMF (n=6) and CAF (n=3) expressing empty vector, were used as controls. **E,** qPCR analysis of *Mmp13* in MMFOsx⁺ transfected with control (scrambled) or si-MMP13 for experiments in Figure 5N-O (data shown as average of 3 different experiments). Each dot represents average of multiple wells per experiment. Source data provided in Source Data file. Supplementary figure 7A was created in BioRender. Furesi, G. (2026) https://BioRender.com/rpuownu.

Supplementary Fig 8

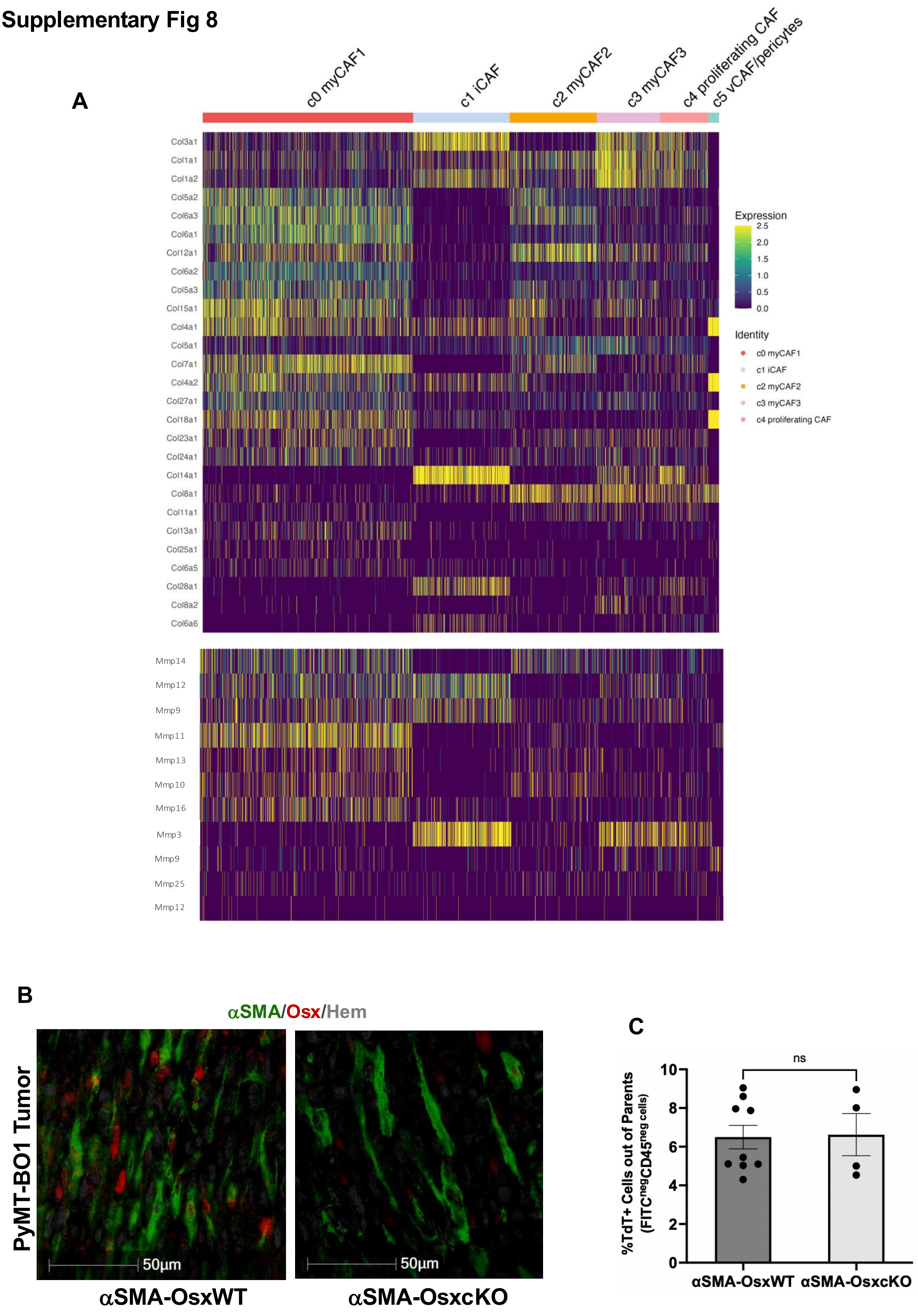

A, Heatmap depicting collagen and extracellular matrix gene expression across stromal cell clusters defined by murine scRNA-seq analysis (n=6). **B,** Representative deconvoluted multiplex immunohistochemistry (mIHC) images of orthotopic PyMT-BO1 breast tumors from αSMA-OsxWT and αSMA-OsxcKO mice stained for Osx (red), αSMA (green), and hematoxylin (gray), corresponding to Figure 4I (n=4). **C,** Flow cytometric quantification of $\alpha SMATdT^{+}$ cells as a percentage of the parent stromal gate (live cells GFP^{neg} $CD45^{neg}$) in αSMA-OsxWT (n=9) and αSMA-OsxcKO tumors (n=4). An unpaired two-tailed Student T-test was used to determine significance in C. Source data and exact p-values are provided in Source Data file.

Supplementary Figure 9

A

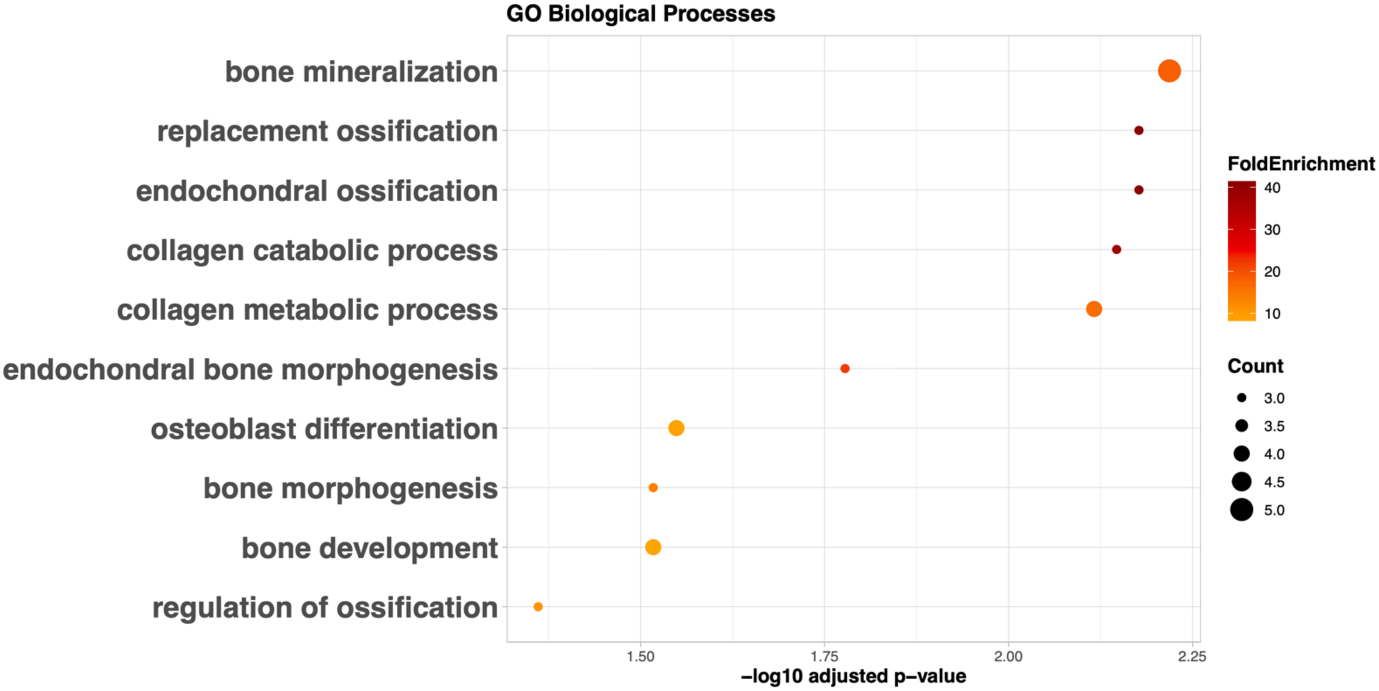

B

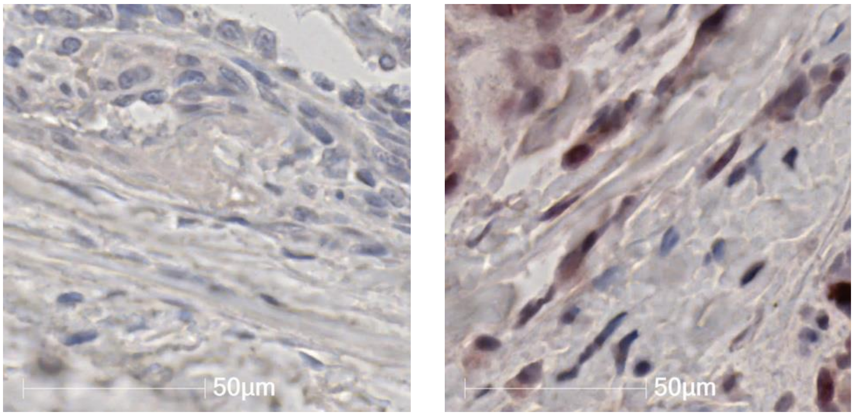

Osx staining-Tonsil **Osx staining-TNBC biopsy**

A, Gene Ontology (GO) pathway analysis of murine scRNA-seq data showing enrichment of osteogenic pathways within the OsteoLin gene signature comprising 54 genes (n=6). P-values were calculated by enrichGO with one-sided Fisher's exact test, and multiple testing correction was performed using the Benjamini-Hochberg method. **B,** Representative IHC images of human tonsil (n=1) and triple-negative breast cancer (TNBC) biopsy tissues (n=13) stained with anti-OSX antibody used as negative and positive control staining, respectively, for images in Figure 7.

Supplementary Table 1

Reagent List

**Flow Cytometry Antibodies**	**Fluorophore**	**Clone**	**Source**	**Identifier**	**Dilution**
CD16/32	blocker	93	Biolegend	Cat# 101302	1:500
CD45	BV605	30-F11	Biolegend	Cat# 103155	1:200
CD45.1	APC	A20	Biolegend	Cat# 110714	1:200
CD45.2	FITC	104	Biolegend	Cat# 109806	1:200
CD31	APC	MEC13.3	Biolegend	Cat# 102510	1:200
Ter119	Pacific Blue	Ter-119	Biolegend	Cat# 116232	1:200
Ter119	BV605	Ter-119	Biolegend	Cat# 116239	1:200
CD140b(Pdgfrβ)	APC-eFluor780	APB5	Invitrogen	Cat# 47-1402-82	1:200
CCD140a (Pdgfrα)	BV421	APA5	Biolegend	Cat# 135923	1:200
CD146	PerCP-Cy5.5	ME-9F1	Biolegend	Cat# 134709	1:200
Fixable viability dye	eFlour780	-	Invitrogen	Cat# 65-0865-14	1:1000
Zombie UV fixable dye	Indo-1	-	Biolegend	Cat# 423108	1:200

**ICH Antibodies**	**Source**	**Identifier**	**Dilution**
Anti-human/mouse αSMA	Abcam	Cat# ab5694	1:1500 (H), 1:200 (M)
Anti-human/mouse OSX	Abcam	Cat# ab227820 or ab209484	1:200 (H), 1:500 or 1:100 (M)
Anti-human PDGFRα	Cell signaling	Cat# 5241S	1:200
Anti-mouse PDGFRα	Cell signaling	Cat# 3174S	1:500
Anti-Human PanCK	Novus	Cat# NBP2-29429	1:1000
Anti-mouse Epcam	Cell signaling	Cat# 93790	1:300
Anti-GFP	Abcam	Cat# 183734	1:200
Anti-human/mouse MMP13	Abcam	Cat# ab219620-1001	1:100
Anti-human COL14a1	Cell signaling	Cat# 61964S	1:300
Anti-human MCAM (CD146)	Cell signaling	Cat# 81701S	1:200
**IF Antibodies**			1:1000
Anti-mousePDGFRβ	Cell signaling	Cat# 3169S	1:200
Anti-mouse FITC-CD45	Biolegend	Cat#103108	!:500
Anti-goat Alexa 488	Abcam	Cat# ab15007	1:800
DAPI	Abcam	Cat# ab228549	1:2000

Supplementary Table 2

New ID	RCB score	Legal Sex	BRCA1/2 (Y=1, N=0)	Diagnosis Date	Cancer Stage (T)	Cancer Stage (N)	ER positive (Y= 1/N =0)	PR positive (Y=1/N=0)	HER2 IHC (Y=1/N=0)
KN522-1	0	F	0	TNBC	3	0	0	0	0
KN522-2	0	F	0	TNBC	3	0	0	0	0
KN522-3	0	F	0	TNBC	2	0	0	0	0
KN522-4	0	F	0	TNBC	2	0	0	0	1
KN522-6	0	F	1	TNBC	3	3	0	0	1
KN522-7	0	F	0	low PR+	3	3	0	1	1
KN522-8	2	F	1	TNBC	3	0	0	0	0
KN522-9	2	F	0	TNBC	4a	1	0	0	
KN522-10	2	F		low ER+	2	1	1 (weakly)	0	0
KN522-11	2	F		TNBC					
KN522-12	3	F	0	TNBC	4b	1	0	0	0
KN522-13	3	F	0	TNBC	2	1	0	0	0
KN522-14	3	F	0	TNBC		1	0	0	1

Characteristics of breast cancer patients used for multiplex immunohistochemistry.
Patients were treated with neoadjuvant chemotherapy plus immunotherapy (Keynote522),
followed by evaluation of the Residual Cancer Burden (RCB) score. Responders were selected based on RCB=0, and Non-Responders on RCB=II-III
