# Supplementary material for: Bone-derived Osterix+ osteolineage cells are a source of tumor-promoting myofibroblastic cancer-associated fibroblasts in breast cancer
Source: Nat Commun. 2026 Jun 11;17:7452. doi: 10.1038/s41467-026-73980-7 (PMC13408961; doi:10.1038/s41467-026-73980-7)
Supplement: Supplementary file 2 — Description of Additional Supplementary Files [file 41467_2026_73980_MOESM2_ESM.pdf]

Description of Additional Supplementary Files

Supplementary Data 1: The marker table was generated by applying the FindAllMarkers function to the CAF/pericyte Seurat object at a clustering resolution of 0.35. Differential expression analysis was performed using the Wilcoxon rank-sum test implemented in the function, and genes are ordered by adjusted p-value and cluster identity.

Supplementary Data 2: Filtered tables used for visualization were generated from the GO enrichment analysis of clusters identified at resolution = 0.35. GO terms were filtered based on adjusted p-value and gene count (see code for details). The differential expression comparison used for each GO enrichment analysis is indicated in the corresponding Excel sheet name. Overrepresentation analysis was performed using a one-sided Fisher’s exact test.

Supplementary Data 3: Raw tables were generated from the GO enrichment analysis of clusters identified at resolution = 0.35. The differential expression comparison used for each GO enrichment analysis is indicated in the corresponding Excel sheet name. Overrepresentation analysis was performed using a one-sided Fisher’s exact test.

Supplementary Data 4: Tables generated from the human scRNA-seq data analysis, including the marker table for human CAF clusters identified at resolution = 0.1, GSEA results for cluster markers, and GO enrichment analysis of marker genes. Additional details are provided in the header of each sheet.

Supplementary Data 5: Differential gene expression profile of myCAF1 marker genes selected as the osteolineage signature. The table was derived from the marker table generated using FindAllMarkers(), with corresponding human gene symbols added. Default parameters were used for the FindAllMarkers() analysis.
