# Supplementary material for: Bone-derived Osterix+ osteolineage cells are a source of tumor-promoting myofibroblastic cancer-associated fibroblasts in breast cancer
Source: Nat Commun. 2026 Jun 11;17:7452. doi: 10.1038/s41467-026-73980-7 (PMC13408961; doi:10.1038/s41467-026-73980-7)
Supplement: Supplementary file 8 — Reporting Summary [file 41467_2026_73980_MOESM8_ESM.pdf]

nature portfolio

Corresponding author(s): Roberta Faccio

Last updated by author(s): 2026.05.11

Reporting Summary

Nature Portfolio wishes to improve the reproducibility of the work that we publish. This form provides structure for consistency and transparency in reporting. For further information on Nature Portfolio policies, see our Editorial Policies and the Editorial Policy Checklist.

Statistics

For all statistical analyses, confirm that the following items are present in the figure legend, table legend, main text, or Methods section.

n/a	Confirmed	
☐	☒	The exact sample size (*n*) for each experimental group/condition, given as a discrete number and unit of measurement
☐	☒	A statement on whether measurements were taken from distinct samples or whether the same sample was measured repeatedly
☐	☒	The statistical test(s) used AND whether they are one- or two-sided<br>*Only common tests should be described solely by name; describe more complex techniques in the Methods section.*
☒	☐	A description of all covariates tested
☐	☒	A description of any assumptions or corrections, such as tests of normality and adjustment for multiple comparisons
☐	☒	A full description of the statistical parameters including central tendency (e.g. means) or other basic estimates (e.g. regression coefficient) AND variation (e.g. standard deviation) or associated estimates of uncertainty (e.g. confidence intervals)
☐	☒	For null hypothesis testing, the test statistic (e.g. *F*, *t*, *r*) with confidence intervals, effect sizes, degrees of freedom and *P* value noted<br>*Give P values as exact values whenever suitable.*
☒	☐	For Bayesian analysis, information on the choice of priors and Markov chain Monte Carlo settings
☒	☐	For hierarchical and complex designs, identification of the appropriate level for tests and full reporting of outcomes
☒	☐	Estimates of effect sizes (e.g. Cohen's *d*, Pearson's *r*), indicating how they were calculated

Our web collection on statistics for biologists contains articles on many of the points above.

Software and code

Policy information about availability of computer code

Data collection	BD FACSDiva v9.0 or Sony Sinergy cell sorter for FACS. BD LSRFortessa X-20 Cell Analyzer for flow cytometry. ORCA-Flash4.0 LT3 Digital CMOS camera and Leica SP8 confocal microscope for 3D culture. Bond Rxm (Leica Biosystems) and Zeiss AxioScan 7 microscope for multiplex immunohistochemistry. Zeiss LSM880 airyscan inverted confocal microscope for confocal images. A Zeiss LSM 880 laser-scanning microscope equipped with a Plan-Apochromat 20×/0.8 M27 objective was used for second harmonic generation (SHG) imaging under two-photon excitation. 7300 Real Time PCR System was used for qPCR. EL800 microplate reader for MTT assay. For scRNAseq sample preparation on the 10x Genomics platform, the Chromium Next GEM Single Cell 3’ Kit v3.1, 16 rxns (PN-1000268), Chromium Next GEM Chip G Single Cell Kit, 48 rxns (PN-1000120), and Dual Index Kit TT Set A, 96 rxns (PN-1000215) were used. Normalized libraries were sequenced on NovaSeq6000 S4 Flow Cell.

Data analysis	NIS-Elements (v5.42.04) 64-bit software for 3D culture. FlowJo (v10.9.0) for flow cytometry. and IHC analyses HALO image analysis platform (Indica Labs, Deconvolution v1.1.1, Multiplex IHC v.3.2.3 algorithms) were used for multiplex immunohistochemistry. ZEN Black (ZEISS Efficient Navigation) software (Zeiss), and ImageJ (v2.3.0) for confocal images. KCJunior software (v1.4) was used for MTT assay. ImageJ (version 2.3.0) was used for Crystal violet assay and Cluster analyses in 3D cultures. ImageJ (version 1.54d) was used to determine fluorescence Intensity in 3D culters. Imaris software (Bitplane, Oxford Instruments, v10.1.1) was used for the quantification of collagen-covered area. 7300 System software (v1.4.0) was used for qPCR analyses. Cellranger (v7.0.0) was used for alignment of FASTQ files to a customized GRCm39 reference genome. Sc-RNASeq data were analyzed using SR_4.5.0, harmony_1.2.3, RColorBrewer_1.1-3, dplyr_1.1.4, tidyverse_2.0.0, Seurat_5.3.0, SeuratObject_5.1.0, ggplot2_3.5.2, org.Mm.eg.db_3.21.0, AnnotationDbi_1.70.0, org.Hs.eg.db_3.21.0,Orthology.eg.db_3.21.0, clusterProfiler_4.16.0, harmony_1.2.3, readxl_1.4.5, data.table_1.17.0, gridExtra_2.3, msigdbr_7.5.1<br>fgsea_1.34.0, GEOquery_2.76.0, limma_3.64.0, ggpubr_0.6.0, ssGSEA2_1.0.0, SummarizedExperiment_1.38.0, RTCGA_1.38.0, TCGAbiolinks_2.38.0

For manuscripts utilizing custom algorithms or software that are central to the research but not yet described in published literature, software must be made available to editors and reviewers. We strongly encourage code deposition in a community repository (e.g. GitHub). See the Nature Portfolio guidelines for submitting code & software for further information.

Data

Policy information about availability of data

All manuscripts must include a data availability statement. This statement should provide the following information, where applicable:

- Accession codes, unique identifiers, or web links for publicly available datasets
- A description of any restrictions on data availability
- For clinical datasets or third party data, please ensure that the statement adheres to our policy

Data Availability
The single-cell RNA sequencing data generated in this study were deposited at Gene Expression Omnibus under GSE292888 [https://www.ncbi.nlm.nih.gov/geo/query/acc.cgi?acc= GSE292888]. The processed feature barcode matrices are included in the same accession. The processed human scRNA-seq data were retrieved from https://zenodo.org/records/7540604 [https://doi.org/10.5281/zenodo.7540604] and GSE176078 [https://www.ncbi.nlm.nih.gov/geo/query/acc.cgi?acc=GSE176078] as specified in the text. Public human microarray data were retrieved from GSE8977 [https://www.ncbi.nlm.nih.gov/geo/query/acc.cgi?acc=GSE8977] and GSE9014 [https://www.ncbi.nlm.nih.gov/geo/query/acc.cgi?acc=GSE9014] using getGEO in R. The TCGA-BRAC data bulk RNA-seq was retrieved using the TCGAbiolinks function in R (see codes" Human-2-0_prepare-tcga-brca-data.R") and the clinical metadata were retrieved from the supplementary tables of the TCGA-BRAC 27653561 (1) and the survivalTCGA function. Raw flow cytometry data and deconvoluted mIHC images from patients' biopsies are available at https://doi.org/10.5281/zenodo.20127527. The remaining data are available within the Article, Supplementary Information, and Source Data file.

(1) Grossman, R. L. et al. Toward a Shared Vision for Cancer Genomic Data. N Engl J Med 375, 1109-1112 (2016). https://doi.org/10.1056/NEJMp1607591

Code Availability
Codes for the tdTomato sequence used for creating the customized reference genome are available at https://zenodo.org/records/1965560263 and documented in the Zenodo page. Codes and relevant information for data analysis and visualization in R are available at https://doi.org/10.5281/zenodo.20097508

Research involving human participants, their data, or biological material

Policy information about studies with human participants or human data. See also policy information about sex, gender (identity/presentation), and sexual orientation and race, ethnicity and racism.

Reporting on sex and gender	Women with a diagnosis of TNBC breast cancer were eligible for this study
Reporting on race, ethnicity, or other socially relevant groupings	Patients were not excluded from participation in this study based on race, ethnicity, or any other socially relevant construct, as cancer affects women of all races and ethnicities. All race and ethnicity data collected was self-reported by each enrolling patient. Data presented in this manuscript was blinded to race, ethnicity, and all other socially relevant constructs.
Population characteristics	A cohort of non metastatic TNBC patients was subjected to neoadjuvant standard-of-care regimen (Keynote522) consisting of pembrolizumab (200 mg) every 3 weeks plus paclitaxel and carboplatin weekly for the first 12 weeks, followed by evaluation of Residual Cancer Burden (RCB) score. Responders were selected based on RCB=0 and Non-Responders on RCB=II-III.
Recruitment	Patients gave permission to use biopsies and clinical info for research purposes
Ethics oversight	Clinically annotated diagnostic biopsies from TNBC patients were obtained from Department of Pathology at Washington University IRB #201105394.

Note that full information on the approval of the study protocol must also be provided in the manuscript.

Field-specific reporting

Please select the one below that is the best fit for your research. If you are not sure, read the appropriate sections before making your selection.

☒ Life sciences ☐ Behavioural & social sciences ☐ Ecological, evolutionary & environmental sciences

Life sciences study design

All studies must disclose on these points even when the disclosure is negative.

Sample size	Sample size was calculated based on our previous published data and preliminary experiments.
Data exclusions	Outlier test was performed to determine whether any data point should have been excluded. (P<0.05 (two sided)
Replication	In vitro experiments included technical and biological replicates and were performed at least 3 times. In vivo experiments were done with at least 5-6 mice per group. (the number of mice used for each experiment is specified in the figure legends). All attempts at replication were successful.
Randomization	Mice were randomized before treatment.
Blinding	Blinding was not possible due to the repeated measurements of tumor progression over time and delivery of specific treatments.

Reporting for specific materials, systems and methods

We require information from authors about some types of materials, experimental systems and methods used in many studies. Here, indicate whether each material, system or method listed is relevant to your study. If you are not sure if a list item applies to your research, read the appropriate section before selecting a response.

Materials & experimental systems

n/a	Involved in the study
☐	☒ Antibodies
☐	☒ Eukaryotic cell lines
☒	☐ Palaeontology and archaeology
☐	☒ Animals and other organisms
☐	☒ Clinical data
☒	☐ Dual use research of concern
☒	☐ Plants

Methods

n/a	Involved in the study
☒	☐ ChIP-seq
☐	☒ Flow cytometry
☒	☐ MRI-based neuroimaging

Antibodies

Antibodies used

<Flow cytometry, Anti Mouse>
CD16/32 (blocker, clone 93, Biolegend, Cat# 101302, 1:500), CD45-BV605 (clone 30-F11, Biolegend, Cat# 103155, 1:200), CD45.1-APC (clone A20, Biolegend, Cat# 110714, 1:200), CD45.2 (clone 104, Biolegend, Cat# 109806, 1:200), CD31-APC (clone MEC13.3, Biolegend, Cat# 102510, 1:200),Ter119-Pacific Blue (clone Ter-119, Biolegend, Cat# 116232, 1:200), Ter119-BV605 (clone Ter-119, Biolegend, Cat# 116239, 1:200), CD140b(Pdgfrβ) APC-eFluor780 (clone APB5, Invitrogen, Cat# 47-1402-82, 1:200), CD140a (Pdgfrα)-BV421 (clone APA5, Biolegend, Cat# 135923, 1:200), CD146-PerCP-Cy5.5 (clone ME-9F1, Biolegend, Cat# 134709, 1:200), Fixable viability dye eFlour780 (Invitrogen, Cat# 65-0865-14, 1:1000), Zombie UV fixable dye Indo-1 (Biolegend, Cat# 423108,1:200).
<IHC>
Anti-human/mouse αSMA (Abcam , Cat# ab5694, 1:1500 (Human), 1:200 (Mouse), Anti-human/mouse OSX (Abcam ,Cat# ab227820 or ab209484, 1:200 (Human), 1:500 or 1:100 (Mouse), Anti-human PDGFRα (Cell signaling , Cat# 5241S, 1:200), Anti-mouse PDGFRα (Cell signaling, Cat# 3174S , 1:500), Anti-Human PanCK (Novus, Cat# NBP2-29429, 1:1000), Anti-mouse Epcam,(Cell signaling , Cat# 93790, 1:300), Anti-GFP (Abcam, Cat# 183734, 1:200), Anti-human/mouse MMP13 (Abcam, Cat# ab219620-1001, 1:100), Anti-human COL14A1 (Cell Signaling, Cat# 61964S 1:300, Anti-human MCAM (Cell Signaling Technology, Cat# 81701S 1:200).
<IF>
Anti-mouse PDGFRβ (Cell signaling, Cat# 3169S, 1:200), Anti-mouse FITC-CD45 (Biolegend, Cat#103108, 1:500), Anti-goat Alexa 488 (Abcam, Cat# ab15007, 1:800), DAPI (Abcam, Cat# ab228549, 1:2000).

Validation

Validation of commercial antibodies used in this study was done by the manufacturer, and details of the validation are described on the manufacturer's website.
<Flow cytometry, Anti Mouse>
CD16/32 https://www.biolegend.com/en-ie/products/purified-anti-mouse-cd16-32-antibody-190?GroupID=BLG9237, CD45-BV605 https://www.biolegend.com/fr-lu/products/brilliant-violet-605-anti-mouse-cd45-antibody-8721, CD45.1-APC https://www.biolegend.com/en-ie/products/apc-anti-mouse-cd45-1-antibody-2319 , CD45.2-FITC https://www.biolegend.com/nl-be/products/fitc-anti-mouse-cd45-2-antibody-6?GroupID=BLG7007, CD31-APC, https://www.biolegend.com/en-ie/products/apc-anti-mouse-cd31-antibody-375?GroupID=BLG10531, Ter119-Pacific Blue, https://www.biolegend.com/de-de/products/pacific-blue-anti-mouse-ter-119-erythroid-cells-antibody-6137, Ter119-BV605 https://www.biolegend.com/fr-fr/products/brilliant-violet-605-anti-mouse-ter-119-erythroid-cells-antibody-8839, CD140b https://www.thermofisher.com/antibody/product/CD140b-PDGFRB-Antibody-clone-APB5-Monoclonal/47-1402-82, CD140a https://www.biolegend.com/nl-nl/products/brilliant-violet-421-anti-mouse-cd140a-antibody-17921, CD146-PerCP-Cy5.5 https://www.biolegend.com/nl-nl/products/percp-cyanine5-5-anti-mouse-cd146-

antibody-7872, Fixable viability dye eFlour780 https://www.thermofisher.com/order/catalog/product/65-0865-18, Zombie UV fixable dye Indo-1 https://www.biolegend.com/de-at/products/zombie-uv-fixable-viability-kit-9336.

<IHC>

Anti-human/mouse αSMA https://www.abcam.com/en-us/products/primary-antibodies/alpha-smooth-muscle-actin-antibody ab5694?srsltid=AfmBOoqSnUeEzcK9JNJbMEzBhwaPPCPuJMIjk9MhbB456EjFrKtKJH81 , Anti-human/mouse OSX https://www.abcam.com/en-us/products/primary-antibodies/sp7-osterix-antibody-epr21034-bsa-and-azide-free-ab227820?srsltid=AfmBOoodMjwulmuCoa8SHjyEWFH1Lm-Qa7aLGMDP6ggw6NGMZh48HGn9 or https://www.abcam.com/en-us/products/primary-antibodies/sp7-osterix-antibody-epr21034-ab209484?srsltid=AfmBOorW2lzlv0K20CEc9sSxPpcYmHEqwOj-D6SP4f1S_hxmIg88Woa4 , Anti-human PDGFRα https://www.cellsignal.com/products/primary-antibodies/pdgf-receptor-a-d13c6-xp-rabbit-mab/5241?srsltid=AfmBOoqAVyktEXeqJmmsBU-rYowwlcObWwqJuA4LLXfb-6qgxs_lWMqY , Anti-mouse PDGFRα https://www.cellsignal.com/products/primary-antibodies/pdgf-receptor-a-d1e1e-xp-rabbit-mab/3174, Anti-Human PanCK https://www.novusbio.com/primary-antibodies/cytokeratin-pan?gad_source=1&gad_campaignid=20381572490&gbraid=0AAAAAD_u9xatNZpLF_KC2iNqHURzwoWZC&gclid=Cj0KCQjwxJvBBhDuARIsAGUgNfiiXlVKVGvJ-0OYlitaw43k64JUeCUg_DtasKrO5jPNGHA9VTIeO_IaAmD5EALw_wcB&gclsrc=aw.ds , Anti-mouse Epcam https://www.cellsignal.com/products/primary-antibodies/epcam-e6v8y-xp-rabbit-mab/93790, Anti-GFP https://www.abcam.com/en-us/products/primary-antibodies/gfp-antibody-epr14104-ab183734?srsltid=AfmBOopsMPv3TUH1VSMBljde94DTsxeq1ewnMRxMsZoWoqMWx4NQzkcp , Anti-human/mouse MMP13 https://www.abcam.com/en-us/products/primary-antibodies/mmp13-antibody-epr21778-ab219620?srsltid=AfmBOoo7m3ZxBQ7hvbtVB0ilq41N1A2aSKtfiWp5b7P1ie2PYSd2KCey#

Anti-human COL14A1 https://www.cellsignal.com/products/primary-antibodies/col14a1-e5w8s-rabbit-monoclonal-antibody/61964

Anti-human MCAM (CD146) https://www.cellsignal.com/products/81701/datasheet?images=1&protocol=0&srsltid=AfmBOopif4z5epBY36LO2cPRgOuBlPJgDGgZbz8WVkeIWCqYXumyAQlk

<IF>

Anti-mouse PDGFRβ https://www.cellsignal.com/products/primary-antibodies/pdgf-receptor-b-28e1-rabbit-mab/3169?srsltid=AfmBOorpUPN5FvZAi1hlgYtffSJkfpQw57co8zvsSorMyXGI8zA-VzAg , Anti-mouse FITC-CD45 https://www.biolegend.com/en-gb/products/fitc-anti-mouse-cd45-antibody-99, Anti-goat Alexa 488 https://www.abcam.com/en-us/products/secondary-antibodies/goat-rabbit-igg-h-l-alexa-fluor-488-ab150077?srsltid=AfmBOopeLPaL87YQ9egTsC-oGLL5vQe3zujfqZw2JHQyUGmC5nfXKodU, DAPI https://www.abcam.com/en-us/products/assay-kits/dapi-staining-solution-ab228549.

Eukaryotic cell lines

Policy information about cell lines and Sex and Gender in Research

Cell line source(s)	Polyoma middle tumor-antigen murine mammary tumor cells (PyMT, C57BL/6), and mCherry-conjugated PyMT (from Dr. DeNardo, Washington University in St. Louis, MO, USA), bone-trophic GFP- and firefly-luciferase-conjugated PyMT-BO1 (from Dr Weilbaecher, Washington University in St. Louis, MO, USA), Primary mammary fibroblasts (MMF; C57BL/6) (from Dr. S.A. Stewart, Washington University in St. Louis, MO, USA), GFP- and Fluc-conjugated 4T1 murine mammary tumor cells (BALB/c; provided by David Piwnica-Worms, The University of Texas MD Anderson, Houston TX, USA), Met-1 breast cancer cell line (FVB) and immortalized CAFs (FVB; provided by Dr. D. Longmore, Washington University in St. Louis, MO, USA) were cultured at 37°C with 5% CO2 in complete media DMEM supplemented with 10% heat-inactivated FBS, 100 µg/ml streptomycin, 100 IU/ml penicillin, and 1 mM sodium pyruvate. All cell lines were tested for Mycoplasma every 2 months. Aliquots for each cell line were used for 1 month after thawing.
Authentication	No cell line authentication was performed beyond information from suppliers.
Mycoplasma contamination	All cell lines were tested for Mycoplasma every 2 months. Aliquots for each cell line were used for a maximum of one month after the initial thaw.
Commonly misidentified lines<br>(See ICLAC register)	No commonly misidentified cell lines were used.

Animals and other research organisms

Policy information about studies involving animals; ARRIVE guidelines recommended for reporting animal research, and Sex and Gender in Research

Laboratory animals	Wild-type (WT) C57BL/6, FVB/N, BALB/c, B6.SJL-Ptprca Pepcb/BoyJ (CD45.1 B6, JAX #002014), Sp7Cre (B6.Cg-Tg(Sp7-tTAtetO-EGFP/Cre), and TdT (B6.Cg-Gt(ROSA)26SorTm9(CAG-tdTomato)Hze/J were purchased from The Jackson Laboratory at 6-8 weeks of age. After delivery, mice were allowed to acclimatize to the new environment for at least 2 weeks. Sp7Cre mice, which harbor a tetracycline-responsive Osx promoter driving Cre (OsxCre), were crossed with TdT reporter mice to generate OsxCre;TdT mice. To suppress Cre expression, 200ppm doxycycline (doxy) was added to the chow (Test Diet #1816332–203, Purina, MO, USA) and administered to specific groups of mice until weaning (P25); thereafter, pups were transitioned to standard rodent chow. αSMACreERT2 transgenic mice were generated by Dr. Ivo Kalajzic (University of Connecticut Health Center, Farmington, CT) (53). Osx/Sp7fl/fl mice were generated in the laboratory of Dr. Benoit de Crombrugghe (University of Texas MD Anderson Cancer Center, Houston, TX)57. These strains were crossed with TdT reporter mice to generate αSMACreERT2;Osx/Sp7fl/fl;TdT mice.
Wild animals	The study did not involve wild animals
Reporting on sex	Because the breast cancer cell lines used in this study were obtained from female mice, we have restricted our analyses to females.
Field-collected samples	After delivery, mice were allowed to acclimatize to the new environment for at least 2 weeks. Animals were housed in a pathogen-

	free animal facility at Washington University (St. Louis, MO) with a 12-h light/12-h dark cycle and 20~23°C and 40~60% humidity housing conditions.
Ethics oversight	This study complies with all relevant ethical regulations including protocols approved by the Institutional Animal Care and Use Committee and guidelines set by the Institutional Review Board of Washington University (Protocol ID: 2025-0181) along with federal and state guidelines.

Note that full information on the approval of the study protocol must also be provided in the manuscript.

Clinical data

Policy information about clinical studies

All manuscripts should comply with the ICMJE guidelines for publication of clinical research and a completed CONSORT checklist must be included with all submissions.

Clinical trial registration	*Provide the trial registration number from ClinicalTrials.gov or an equivalent agency.*
Study protocol	*Note where the full trial protocol can be accessed OR if not available, explain why.*
Data collection	*Describe the settings and locales of data collection, noting the time periods of recruitment and data collection.*
Outcomes	*Describe how you pre-defined primary and secondary outcome measures and how you assessed these measures.*

Plants

Seed stocks	*Report on the source of all seed stocks or other plant material used. If applicable, state the seed stock centre and catalogue number. If plant specimens were collected from the field, describe the collection location, date and sampling procedures.*
Novel plant genotypes	*Describe the methods by which all novel plant genotypes were produced. This includes those generated by transgenic approaches, gene editing, chemical/radiation-based mutagenesis and hybridization. For transgenic lines, describe the transformation method, the number of independent lines analyzed and the generation upon which experiments were performed. For gene-edited lines, describe the editor used, the endogenous sequence targeted for editing, the targeting guide RNA sequence (if applicable) and how the editor was applied.*
Authentication	*Describe any authentication procedures for each seed stock used or novel genotype generated. Describe any experiments used to assess the effect of a mutation and, where applicable, how potential secondary effects (e.g. second site T-DNA insertions, mosiacism, off-target gene editing) were examined.*

Flow Cytometry

Plots

Confirm that:

- [x] The axis labels state the marker and fluorochrome used (e.g. CD4-FITC).
- [x] The axis scales are clearly visible. Include numbers along axes only for bottom left plot of group (a 'group' is an analysis of identical markers).
- [x] All plots are contour plots with outliers or pseudocolor plots.
- [x] A numerical value for number of cells or percentage (with statistics) is provided.

Methodology

Sample preparation	Tumors were digested in serum-free media with 2 mg/ml collagenase type I and 2 U/ml DNaseI for 30 min at 37°C. The cell suspension was filtered through 70-µm nylon strainers and washed twice in PBS with 2% FBS. Red blood cells (RBC) were removed using RBC lysis buffer (Sigma-Aldrich #R7757), followed by an additional wash. Cells were then stained in PBS with 0.5% FBS using anti-mouse antibodies (Suppl. Table 2). For blood analyses, blood was collected from mice via cardiac puncture. Red blood cells were removed by two 10-minute incubations with RBC lysis buffer, followed by an additional wash to ensure complete lysis. The remaining cells were then processed for flow cytometric staining and subsequently acquired for analysis.
Instrument	Acquisition was performed on a BD LSRFortessa X-20 Cell Analyzer.
Software	For acquiring the data, dedicated software DIVA (BD) was used. Data were analyzed with FlowJo 10.9.0 software (Tree Star).
Cell population abundance	After sorting, collected cells were acquired via BD LSRFortessa x-20 Cell Analyzer and confirmed the purity was over 95 percentile.
Gating strategy	FSC-A vs FSC-H gating and SSC-A vs SSC-H gating were utilized to exclude doublets. Then, if applicable, live cells were gated based on the negativity on live/dead dye. In tumor samples, FITC positive tumor cells, Terr119 positive red blood cells, and CD31 positive Endothelial cells were excluded before profiling the CD45 negTdTpos population.

- [x] Tick this box to confirm that a figure exemplifying the gating strategy is provided in the Supplementary Information.
