# Supplementary material for: Bone-derived Osterix+ osteolineage cells are a source of tumor-promoting myofibroblastic cancer-associated fibroblasts in breast cancer
Source: Nat Commun. 2026 Jun 11;17:7452. doi: 10.1038/s41467-026-73980-7 (PMC13408961; doi:10.1038/s41467-026-73980-7)
Supplement: Supplementary file 9 — Transparent Peer Review file [file 41467_2026_73980_MOESM9_ESM.pdf]

nature portfolio

Peer Review File

Bone-derived Osterix+ osteolineage cells are a source of tumor-promoting myofibroblastic cancer-associated fibroblasts in breast cancer

Corresponding Author: Professor Roberta Faccio

This file contains all reviewer reports in order by version, followed by all author rebuttals in order by version.

Version 0:

Reviewer comments:

Reviewer #1

(Remarks to the Author)
In the manuscript Furesi et al aim to study the progenitor of CAFs in breast cancer and ask whether their origin dictates their function. They focused on bone marrow derived cells, specifically osteoblasts. By combining genetic mouse models and multiplexed imaging they performed lineage tracing to track the differentiation of osteolineage cells (Osx+) into CAFs and further show by adoptive cell transfer that infiltrating TdT+OsteoLin cells express PDGFRβ and enhance tumor growth in vitro and in vivo. Next, they performed scRNA-sequencing to mesenchymal cells isolated from tumor bearing mice and found that most TdT+ cells are positive for PDGFRβ and known myCAF markers, and are enriched for ECM organization pathways, thus they considered them as a myCAF subset. They then transfected primary normal mammary fibroblasts and CAFs with Osx and found that Osx expressing fibroblasts drive tumor growth in vivo and in vitro and regulate MMP13 expression. Finally, they test the relevance of their findings in human RNA-seq data sets and mIHC staining of breast cancer samples and confirm this myCAF subset expresses an osteolineage signature and correlates with poor prognosis.
CAFs are a heterogeneous population comprising distinct subsets that differ in their transcriptional programs, functions, and cellular origins. Although various cell types have been proposed as potential progenitors of CAFs, a comprehensive understanding of the origins of these subsets remains elusive. This study holds significant potential to shed light on the progenitor cells of myofibroblastic CAFs. Overall, the manuscript is well written and introduces a novel cell type as a potential origin of CAFs, reinforcing the notion that these cells may arise from bone marrow-derived mesenchymal cells. However, to substantiate their claims, the authors should address several key issues. Given the apparent role of Osx in promoting a protumorigenic tumor microenvironment (TME), it would be valuable to investigate whether Osx deficiency in osteoblasts affects their differentiation into CAFs. Additionally, while the authors identify Osx^{+} CAFs as a myCAF subset enriched in ECM remodeling pathways, they provide limited functional validation to support this claim such as collagen deposition and second harmonic generation (SHG) imaging that could strengthen their conclusions.

Major comments
1. Figure 1 – The authors claim that 20% of PDGFRbeta cells are TdT+ (in D) however in B the costaining seems to be 100%. How do these correspond?
In the same figure, the authors aim to show in J the protumorigenic effect of TdT+ osteolineage population by coinjection of PyMT-BO1 tumor cells and TdT+ OsteoLin cells. They nicely show that the tumor volume was higher in the tumor derived TdT+ OsteoLin cells compared the TdT-. However the baseline is missing – it would be good to show that injection of PyMT cells alone results in tumors smaller or similar to those of TdT- coinjection.
2. Figure 2 – in B and C The authors performed scRNA seq on mesenchymal cells from tumor bearing mice and focused their analysis on PDGFRβ expressing cells. Accumulating evidence highlights PDGFRβ as a pericyte marker (DOI: 10.3389/fcvm.2022.876591), however, along the manuscript the authors refer to PDGFRβ as a CAF marker, and exclude the pericyte cluster which expresses this marker in their data as well. The authors should reexamine their analysis to verify they don’t omit cells that are part of the tumor-infiltrating TdT+OsteoLin cells or at least acknowledge this in the text. This is true also for Figure 6 where the authors show that vCAFs are PDGFRβ positive. vCAFs in many datasets are actually pericytes. Following up on this, the authors show the expression of known myCAF and iCAF markers in their data. What are the top markers that come out of their own clustering? Are these the same markers?
3. In Supp Fig 3 the authors claim that “Nuclear localization of Osx was only observed in the TdT+OsteoLin cells, consistent

with its role as an active transcription factor (Fig. 3A), but not in the GFP⁺ tumor cells (Suppl. Fig. 3).” The authors should add brightfield images to show the cells in the frame.
4. In Figure 3 the authors claim that “Osx nuclear staining was observed in subsets of PDGFRα⁺ and αSMA⁺ cells across all tumor models.” To strengthen this claim the authors should add staining with markers such as CD45, Epcam etc.
5. Figure 5 – The authors state that “osteoprogenitor-derived Osx⁺myCAFs promote tumor progression, at least in part, through the release of MMP13.” In order to strength their claims, it would be interesting to test the effect of MMP13 inhibitor on tumor growth in the genetic model (OsxCre;TdT tumor bearing mice) as the effect of MMP13 inhibitor on NMF expressing Osx is not fully convincing.
6. Figure 7 – The authors performed mIHC on a TMA of human breast cancer, stained for Osx, PDGFRa and aSMA and found triple positive cells. However, they didn’t stain for other CAF markers, and didn’t rule out the possibility of Osx expression in another CAF subsets. Moreover. They should quantify the fraction of Osx+ myCAF population.
Minor comments
1. Supp1a – Axis names should be added to the gating strategy of the FACS plots.
2. Supp1b – Why do the authors gate osteolingeage cells as CD31+? Is this typo?
3. Figure 1h – The authors isolated CAFs by plating the tumor mass on tissue culture plate instead of FACS sorting directly from the tumor. It is established that fibroblasts are very plastic and change their phenotype in vitro, usually acquire a myCAF phenotype. Is there a reason for using this method for CAFs isolation rather than FACS sorting?
4. Figure 2b- although the authors sorted mesenchymal cells and gated the negative populations, it seems there is contamination of epithelial cells, macrophages and endothelial cells. They do address this issue in the text, but the fraction of epithelial cells is quite similar to the number of fibroblasts. It raises concerns regarding the identity and quality of the isolated mesenchymal cells.
5. In line 154 there is a typo in the text, instead of cluster 4 they wrote 8.
6. Figure 6 – The authors analyzed scRNA seq data of human breast cancer patients. In the mouse data they showed Osx was highly enriched in mycaf-1 cluster. However, in the human data they don’t show the expression of this marker, although they analyzed the top 50 DEG. Could the authors check the expression level of Osx and explain why it didn’t come up as one of the DEG? If the authors aim to show the similarity between Osx+ mycaf and h_mycaf1, they should at least assess the levels of Osx/Sp7 in human scRNA-seq data.

Reviewer #2

(Remarks to the Author)
In this manuscript Furesi et al. address an important and topical question in cancer-associated fibroblast (CAF) research, regarding whether the source of CAFs is a determinant of their functional contribution to tumour progression. The authors identify a novel role for osteoblast precursors forming a subpopulation of myofibroblastic CAFs in breast cancer with pro-tumorigenic properties, driven at least in part by Osx/Sp7 mediated expression of MMP13. The manuscript presents some interesting findings that may provide novel insight into identifying important tumour promoting components of the tumour microenvironment and therefore present novel opportunities for therapeutic intervention.
Overall, the findings are compelling and novel, particularly in terms of the functional analysis performed using mouse models. However, there are some issues that would need to be addressed before I could recommend that this manuscript was published. The main areas of concern are regarding the scRNA-seq analysis, validation in human tissues and potential discrepancies between some of the mxIHC data presented and the conclusions drawn. Further details of areas that should be addressed are detailed below.

Introduction
The introduction contains inaccuracies in its description of normal fibroblast markers (line 53). Specifically, the statement that SMA is expressed by normal fibroblasts is incorrect, as SMA is not a typical marker for fibroblasts in normal tissues. Similarly, PDGFRβ and FSP1 are not ubiquitously expressed in fibroblasts. Furthermore, these claims are presented without supporting references, which undermines the scientific rigor of the introduction. The authors should revise these statements to accurately reflect the current literature and provide appropriate citations to support their claims.

Results

Figure 1
The authors effectively demonstrate that bone marrow-derived cells constitute a small subset (approximately 20%) of PDGFRβ+ CAFs in orthotopic tumours. However, further control experiments would help to determine whether this is unique to the TME. For instance, the percentage of PDGFRβ+ cells originating from the osteolineage in normal mammary tissue is not reported.
The experimental design in panels H-K raises several questions. The manuscript does not specify how long the cells are cultured prior to re-injection, and there is inconsistency in the tumour models used (PyMT-B01 in panel J versus PyMT in panels I and K) without any justification for this change. Additionally, from the data presented it is not clear whether the TM-TdT− stromal population, isolated using negative selection, is a pure CAF population and likely includes endothelial or mural cells. This may confound the conclusions drawn regarding the osteolineage CAFs having enhanced tumour-promoting properties compared to CAFs derived from other precursors. Employing a positive selection strategy, such as isolating CD45−PDGFRβ+TdT+/− cells, to ensure the same population of cells is being analysed would yield more definitive results.
The interpretation of the results in panel K is not fully supported by the data. The conclusion that osteolineage CAFs acquire tumour-promoting properties in the tumour microenvironment does not align with the co-injection experiments. If this was the case, then these cells would still acquire tumour promoting properties when co-injected into the tumour. Perhaps an

alternative conclusion would be that only a subset of osteolineage mesenchymal cells are recruited and activated as tumour-promoting CAFs, so by co-injecting all bone marrow stromal cells this selection is bypassed.

Figure 2
The single-cell RNA sequencing analysis lacks adequate controls for batch effects, which is particularly concerning given that cells were pooled from different cohorts of mice. The absence of batch integration in the analysis could lead to clustering artifacts that confound the interpretation of TdT+ versus TdT− populations. The authors should re-analyze the data using established batch correction methods (e.g. 31178118) and provide detailed metadata, including mouse identifiers, processing dates, and pooling strategies with the dataset.
As far as I can tell, the analysis relies on a single pooled sample (effectively n=1) for defining differentially expressed genes (DEGs) and generating the “OSX+ myCAF” signature, which is a significant limitation.
The flow cytometry panel also requires further validation, using qPCR or RNA-seq to verify that the populations gated in this way in fact represent the populations identified by scRNA-seq. As presented, the TdT+ cells are determined to be predominantly myCAF based on PDGFRA+ and the absence of alternative iCAF (Ly6c) and vCAF (CD146). PDGFRA is not a de-facto myCAF marker and is in fact typically downregulated on myCAFs compared to quiescent or universal fibroblasts.

Figure 3
The proportion of Osx+ cells within the myCAF population (and other cell populations) should be quantified here. There appear to be discrepancies between the immunohistochemistry and flow cytometry, likely due to differences in gating strategies and marker selection. For example, In the 4T1 model it is evident that the Osx staining is found in cells that are both SMA+ and SMA-, So doesn’t seem to be limited to myCAFs. In the PyMT image shown, the SMA staining profile is indicative of vessels (not CAFs) and again there are clearly OSX+ SMA+ cells here that represent OSX+ mural cells not myCAFs.
The immunohistochemistry data show OSX+ cells in both SMA+ and SMA− populations, while the flow cytometry analysis pre-gates for PDGFRβ+. This suggests that the current approach may overestimate the number of OSX+ myCAFs due to the inclusion of mural cells. The authors should consider repeating the immunohistochemistry with PDGFRβ co-staining and reconciling these findings with the flow cytometry data.
In panels C&D, the definition of “OSX+ myCAFs” is unclear, as the authors note that Osx expression is not accurately detected in the scRNA-seq data. How are Osx+ myCAF defined? The method described for identifying DEGs (“avg_log2_FC *pct.1”) is unconventional and not clear regarding the threshold for calling a gene differentially expressed. Furthermore, it is not clear what comparison is being referred to for identifying genes used to generate the Osx+ myCAF signature. The most appropriate approach would be to compare TdT+ and TdT− cells within the same cluster (e.g., myCAF-0) and to use conventional criteria for identifying DEGs, such as adjusted p-values and fold-change cutoffs.

Figures 4 & 5
These figures are well-presented and do not raise major concerns.

Figure 6
The conclusions drawn in this figure are contingent on the definition of OSX+ CAFs and their associated DEGs, which, as noted above, require further validation and clarification.

Figure 7
The data showing an increased percentage of myCAFs that are OSX+ and/or MMP13+ in non-responders is intriguing. However, the classification of myCAFs as "PDGFRA+/αSMA+" is ambiguous. If the intention is to identify double-positive cells, the notation should be clarified (e.g., PDGFRA+αSMA+). If the analysis includes single-positive populations, it should be repeated using only double-positive cells, as the others are more likely to represent iCAFs or mural cells.
The use of CIBERSORT to distinguish between OSX+ and OSX− myCAFs requires robust validation, given the potential for transcriptional overlap. The authors should describe the validation and quality control steps undertaken for this analysis and, ideally, confirm the results using ground truth validation (e.g. by IHC or flow cytometry); or at the very least using pseudobulk transcriptome simulated by aggregation of scRNA-seq data (as described in the original CIBERSORT publications).
Additionally, the survival analysis requires further validation in independent cohorts and by examining differences across breast cancer subtypes.

General Recommendations
Overall, the manuscript would benefit from greater precision in marker selection and description, improved methodological rigor (including batch effect controls and consistent use of tumour models), and more transparent statistical reporting. The language should be clarified to avoid ambiguous terms and correct typos. It would also be useful for the code used to perform the bioinformatics analysis to be provided for review.

Reviewer #3

(Remarks to the Author)
The manuscript by Furesi et al. identifies a novel bone marrow-derived Osx+ osteoprogenitor cell which becomes a myofibroblast CAF (myCAF) to promote breast cancer progression driven by MMP13. These findings are timely and important for the field, identifying a new population of CAFS that promote breast cancer progression. Classifications of CAFs are complex, and the data included here further characterize these subsets and identify the progenitor cells involved. The studies are well-designed, using bone marrow adoptive transfer to show the origin of the cells, and the data for this are compelling. While the findings are interesting and novel, there are several areas to address:

Major

1. The population of osteoprogenitor cells that are identified as tumor-promoting, Osx+myCAFS, have very low abundance in the primary tumor (0.8-1.2%), which calls into question their relative importance. As indicated by the authors, >90% of the Osx+ cells in the TME were immune populations. What is the relative contribution of the Osx+myCAFS vs other tumor-promoting cells in the TME? In that vein, if Osx and MMP13 have a functional role in breast tumor progression, as suggested by the data, then what contribution do the Osx+CD45+ or Osx+CD45- cells play in the TME? Are they also pro-tumorigenic?
2. The experiment comparing the Tdt+OsteoLin vs Tdtneg cells is a nice control (Fig. 1J); however, other critical controls are lacking. Please include controls to show that the Tdt OsxCre system is not leaky (i.e., that the Tdt is specifically labeling Osx+ cells and is only active once transitioned to standard diet). Appropriate negative controls are also needed for all staining (e.g., PDGFRalpha and beta, etc). Controls for TdtnetOsx+ and negative controls for Osx staining are needed for Fig. 3.
3. There is a strong emphasis on ECM remodeling and how Osx+myCAFS have ECM remodeling properties, but this is not demonstrated in the manuscript. There are no functional collagen remodeling assays included, only gene signatures. See lines 336, 339, and 318. While the data confirm that Osx+myCAFS arise from the bone marrow, and promote tumor progression, there are no functional studies confirming that this is happening because the myCAFs alter the ECM. The data in Fig. 4 suggest that the effect of the Osx+myCAFS on tumor cells is through aggregation rather than proliferation, but this is not necessarily due to changes in the ECM. There either needs to be data demonstrating this or the language around ECM remodeling needs to be tempered.
4. The human data provided in Figure 7 suggests that higher Osx+myCAFs are significantly associated with reduced 10 yr survival in BC patients. Do the Osx+myCAFS increase with tumor growth in the pre-clinical models? The human data suggest that this population may expand with tumor progression, but it is not clear. Related to this, are the Osx+myCAFS the same cells as the CD15-CD34-Ocn+ cells previously identified, or are these distinct populations? The Ocn+ cells were found to increase as the tumor progressed, which links back to my other question.
5. The finding that the Osx+myCAFS do not become pro-tumorigenic until after they reach the TME is intriguing. Why do you think this is, and more importantly, why/how are these cells recruited to the primary tumor, if they do not inherently promote tumor progression until they have encountered the tumor microenvironment?
6. Should the myCAFS be aSMA and PDGFRa double positive? In multiple images it appears that aSMA and PDGFRa are mutually exclusive, which was confusing (e.g., middle panel of MMTV-PyMT and 4T1 orthotopic images in Fig. 3B). On lines 291 and 292 it says that myCAFS were identified by aSMA and PDGFRa staining and this is again referred to on lines 377 and 378. The populations also appear distinct in the human samples in Figure 7. Please clarify - are these supposed to be co-localized? If so, the colors may need to be adjusted to better demonstrate this. If they are distinct populations, please explain the importance of each and which are actually myCAFs.
7. Please provide data or references demonstrating that MMP13i is specific for MMP13. MMPs are highly redundant, and it is likely that the inhibitor used may target multiple MMPs, or that other MMPs are up-regulated in response to MMP13 inhibition. Do other MMPs change with MMP13 inhibition?
8. In the human data, where do the Osx+myCAF equivalent cells cluster on the UMAP projection? The enrichment for the human equivalent of the Osx+myCAF signature is significant as shown in Fig. 6G, but is it a distinct cluster? Please include these cells on a UMAP plot. It says these overlap some with the h-myCAF3 cluster, but the rest of the genes are clustered with h-myCAF1. This is an important point in translating the findings from the mouse to human. It seems like the myCAF populations differ some between human and mouse. Please expand on this. Related to this, on lines 284 and 285 it says the findings demonstrate strong overlap between h-myCAF1 and mouse Osx+myCAFs, but the data do not suggest this (rather they appear to potentially overlap with both h-myCAF1 and h-myCAF3 clusters).

Minor

9. The discussion appears to be one large paragraph. It would help to break it up on lines 363 and 383 into new paragraphs.
10. Higher magnification images of Figure 3 and all staining would be helpful.
11. In figure 4, why was the MET-1 cell line used instead of 4T1s? Was a similar effect seen in 4T1 cells?
12. On line 448, it refers to experiments with either 1:1 or 1:2 stroma-tumor ratios being injected, but only 1:1 was mentioned in the results.
13. For all graphs, the percentage on the y axis should indicate % of what was measured (e.g., % parent, % live, %CD45+, etc).
14. Please provide more information about why the treatment timeline was selected, with 10 consecutive days of treatment, followed by 8 days washout before sacrifice.
15. The correlative data linking the myCAFs with therapeutic response are interesting, but should be clarified to indicate that this is specifically for patients receiving chemotherapy with anti-PD-1, which is only a subset of TNBC patients (those with PD-L1+ tumors).

Reviewer #4

(Remarks to the Author)

This manuscript presents compelling evidence for the existence of a novel subset of cancer-associated myofibroblasts in the breast cancer tumor microenvironment, termed Osx$^+$myCAFs, which originate from Osterix-expressing (Osx$^+$) osteolineage precursors in the bone marrow. The authors used lineage tracing, flow cytometry, single-cell RNA sequencing, and spontaneous/orthotopic murine models of breast cancer to demonstrate that Osx$^+$myCAFs retain an osteoblastic gene signature and contribute to tumor growth via MMP13 secretion. By analyzing the dataset and samples from breast cancer patients, the author showed that the presence of Osx$^+$myCAFs in breast cancer tissue correlates with poor therapeutic response and reduced 10-year survival. The study proposes that the expression of Osx in myCAF is not only a phenotypic marker but also has a pro-tumorigenic role. While the characterization of Osx$^+$myCAFs is thorough and detailed in the manuscript, the investigation of pro-tumorigenic functions of Osx$^+$myCAFs is less convincing. Also, the translational potential of using Osx$^+$myCAFs for diagnostic or therapeutic targeting seems to be limited.

Major points
1. Low percentage of Osx+myCAFs in tumor
In the orthotopic breast cancer mouse model using PyMT cell line, the author found that Osx+myCAFs comprise 0.8-1.2% of the total primary tumor mass (line 103). Then, the author co-injected (Fig. 1I) or co-cultured (Fig. 4E) breast cancer cells with Osx+myCAFs at 1:1 ratio and showed that Osx+myCAFs stimulated tumor growth both in vivo and in vitro. Given the low percentage of Osx+myCAFs in tumor, the pro-tumorigenic effects of Osx+myCAFs seen at 1:1 ratio may raise questions regarding their actual contribution to tumor progression.

2. TdT+OsteoLin from BM failed to increase tumor growth
The author demonstrated that TdT+OsteoLin cells isolated from orthotopic PyMT-BO1 stimulated tumor growth while TdT+OsteoLin cells isolated from bone marrow failed (line 134). The difference between these two sets of cells was not investigated in the manuscript. Did TdT+OsteoLin cells from bone marrow have myCAF signature? Did TdT+OsteoLin cells in BM fail to stimulate tumor growth due to the lack of expression of MMP13?

3. Timing of the presence of Osx+myCAF
What is the timing of detecting Osx+myCAF in the tumor or in the circulating blood? Does Osx+myCAF contribute to the early or late stages of tumor progression?

4. Pro-tumorigenic role of Osx+myCAF
The author concluded from in vitro and in vivo experiments that Osx+myCAF contributes to tumor growth. The analysis of TNBC patients' samples showed that Osx+myCAF may impart therapy resistance, as higher portions of Osx+myCAF were detected in non-responders. The experimental data and clinical data do not appear to support each other. Stimulating tumor growth and inducing therapy resistance are two different functions. Co-injection (cancer cells+Osx+myCAF) mouse model with or without standard-of-care treatment will need to be conducted to investigate the role of Osx+myCAF in therapy resistance.

5. The author only used one MMP13 inhibitor (CL-82198) to show that MMP13 is required for Osx+myCAF pro-tumorigenic effects. More evidence will be needed, such as knockdown or knockout of MMP13 in Osx+myCAF, to confirm the finding.

Minor points
1. The author used matrix-producing CAF (line 76) and myCAF to refer Osx+myCAF. Although there may be an overlap, it would be good to keep the term consistent in the manuscript.

2. For the analyzed 15 TNBC patients receiving standard-of-care treatments, what are these treatments? Including a table listing the treatments in the supplementary data will be helpful. It may be interesting to see if Osx+myCAF causes therapy resistance to a specific treatment.

Version 1:

Reviewer comments:

Reviewer #1

(Remarks to the Author)
The authors have nicely addressed my comments, congratulations on this beautiful study.

Reviewer #2

(Remarks to the Author)
I would like to thank the authors for the extensive revisions made to address concerns raised by myself and other reviewers. I have no further comments to be addressed

Reviewer #3

(Remarks to the Author)
The authors have comprehensively addressed all of my comments and concerns. The new data and discussion points enhance the manuscript, which will be of interest to the readership in its current form.

Reviewer #4

(Remarks to the Author)
Comments
I would like to thank the authors for their revision and additional siMMP13 experiments to support the functional role of MMP13 in mediating the pro-tumorigenic effects of Osx^{+}
CAFs. The authors have addressed my raised issues, and the manuscript is substantially improved.

Open Access This Peer Review File is licensed under a Creative Commons Attribution 4.0 International License, which permits use, sharing, adaptation, distribution and reproduction in any medium or format, as long as you give appropriate credit to the original author(s) and the source, provide a link to the Creative Commons license, and indicate if changes were made.
In cases where reviewers are anonymous, credit should be given to 'Anonymous Referee' and the source.
The images or other third party material in this Peer Review File are included in the article's Creative Commons license, unless indicated otherwise in a credit line to the material. If material is not included in the article's Creative Commons license and your intended use is not permitted by statutory regulation or exceeds the permitted use, you will need to obtain permission directly from the copyright holder.
To view a copy of this license, visit https://creativecommons.org/licenses/by/4.0/

We thank the reviewers for their thoughtful and constructive comments, which have significantly strengthened our manuscript. We have carefully addressed all comments, including reanalyzing the snRNA-sequencing datasets to include vCAFs, performing functional studies assessing the impact of osteolineage CAFs on ECM remodeling, conducting MMP13 knockdown in osteolineage cells, and generating a mouse model with conditional deletion of Sp7/Osx in αSMA⁺ cells to define the role of the Osx–MMP13 stromal axis in tumor growth. We have also included additional controls and revised the manuscript for clarity.

Below, we provide a point-by-point response. Reviewer comments are shown in black, and our responses are provided in blue. Changes in the main manuscript are also indicated in blue.

Reviewer #1 (Remarks to the Author): with expertise in CAFs

In the manuscript Furesi et al aim to study the progenitor of CAFs in breast cancer and ask whether their origin dictates their function. They focused on bone marrow derived cells, specifically osteoblasts.
By combining genetic mouse models and multiplexed imaging they performed lineage tracing to track the differentiation of osteolineage cells (Osx+) into CAFs and further show by adoptive cell transfer that infiltrating TdT+OsteoLin cells express PDGFRβ and enhance tumor growth in vitro and in vivo. Next, they performed scRNA-sequencing to mesenchymal cells isolated from tumor bearing mice and found that most TdT+ cells are positive for PDGFRβ and known myCAF markers, and are enriched for ECM organization pathways, thus they considered them as a myCAF subset. They then transfected primary normal mammary fibroblasts and CAFs with Osx and found that Osx expressing fibroblasts drive tumor growth in vivo and in vitro and regulate MMP13 expression. Finally, they test the relevance of their findings in human RNA-seq data sets and mIHC staining of breast cancer samples and confirm this myCAF subset expresses an osteolineage signature and correlates with poor prognosis.
CAFs are a heterogeneous population comprising distinct subsets that differ in their transcriptional programs, functions, and cellular origins. Although various cell types have been proposed as potential progenitors of CAFs, a comprehensive understanding of the origins of these subsets remains elusive. This study holds significant potential to shed light on the progenitor cells of myofibroblastic CAFs. Overall, the manuscript is well written and introduces a novel cell type as a potential origin of CAFs, reinforcing the notion that these cells may arise from bone marrow-derived mesenchymal cells. However, to substantiate their claims, the authors should address several key issues. Given the apparent role of Osx in promoting a protumorigenic tumor microenvironment (TME), it would be valuable to investigate whether Osx deficiency in osteoblasts affects their differentiation into CAFs. Additionally, while the authors identify Osx⁺ CAFs as a myCAF subset enriched in ECM remodeling pathways, they provide limited functional validation to support this claim such as collagen deposition and second harmonic generation (SHG) imaging that could strengthen their conclusions.

We thank the reviewer for recognizing the significance of the study and acknowledging that the origin of the various CAF subsets remains elusive. We appreciate the constructive feedback and have incorporated new data to further strengthen our conclusions.

The reviewer also suggested that "*it would be valuable to investigate whether Osx deficiency in osteoblasts affects their differentiation into CAFs*". We apologize for not explaining the role of Osx in osteoblast biology more clearly in the original submission. Osx is a key transcription factor needed for osteoblast differentiation and bone deposition (PMID:11792318). Osx KO mice die at birth due to respiratory failure since the skeletal elements in the ribs remain cartilaginous and cannot support breathing. Therefore, it is not feasible to assess whether loss of Osx in osteoblasts affects their ability to give rise to CAFs, as osteoblast differentiation itself is abrogated.

To address whether Osx deletion in vivo influences CAF development, we generated mice carrying the inducible aSMA^{ERT2}Cre crossed with Osxfl/fl alleles. We selected the aSMACre driver because our single-cell RNA sequencing data show aSMA expression in osteolineage CAFs. Cre recombination was induced at 12 weeks of age using five consecutive tamoxifen (TAM) injections starting on the day of tumor cell inoculation. We confirmed efficient deletion of Osx within the tumor microenvironment; however, we did not observe changes in CAF numbers compared to WT controls (**New Suppl. Fig. 8B, C**). Importantly, Osx deletion resulted in a significant reduction in tumor growth relative to WT littermate controls, accompanied by decreased collagen deposition (**New Fig. 4H-K**). These findings demonstrate that while Osx is not required for CAF development, its expression within CAFs is critical for their pro-tumorigenic and ECM-remodeling functions.

Major comments
1. Figure 1 – The authors claim that 20% of PDGFRbeta cells are TdT+ (in D) however in B the costaining seems to be 100%. How do these correspond?

We apologize for the confusion. In the original Fig 1B we wanted to show the presence of elongated OsxTdT$^+$ cells in the TME. However, the selected field of view was not representative of the overall frequency of OsxTdT$^+$ cells within the stromal compartment. This higher-magnification image was selected to highlight the OsxTdT$^+$ cell morphology rather than their relative abundance. We have now included a new lower-magnification image demonstrating that only a subset of PDGFRβ$^+$ cells are OsxTdT$^+$ (**New Fig. 1B**), consistent with the quantification shown in **Fig. 1E**.

In the same figure, the authors aim to show in J the protumorigenic effect of TdT+ osteolineage population by coinjection of PyMT-BO1 tumor cells and TdT+ OsteoLin cells. They nicely show that the tumor volume was higher in the tumor derived TdT+ OsteoLin cells compared the TdT-. However the baseline is missing – it would be good to show that injection of PyMT cells alone results in tumors smaller or similar to those of TdT- coinjection.

We apologize for the omission. We have included tumor cells alone as a baseline control. To improve the clarity of the story, this result has been moved to **New Fig. 3C**, following the additional characterization of OsxTdT$^+$ and OsxTdT^{neg} cells.

2. Figure 2 – in B and C The authors performed scRNA seq on mesenchymal cells from tumor bearing mice and focused their analysis on PDGFRβ expressing cells. Accumulating evidence highlights PDGFRβ as a pericyte marker (DOI: 10.3389/fcvm.2022.876591), however, along the manuscript the authors refer to PDGFRβ as a CAF marker, and exclude the pericyte cluster which expresses this marker in their data as well. The authors should reexamine their analysis to verify they don't omit cells that are part of the tumor-infiltrating TdT+OsteoLin cells or at least acknowledge this in the text.
This is true also for Figure 6 where the authors show that vCAFs are PDGFRβ positive. vCAFs in many datasets are actually pericytes. Following up on this, the authors show the expression of known myCAF and iCAF markers in their data. What are the top markers that come out of their own clustering? Are these the same markers?

We appreciate the reviewer's point regarding PDGFRβ expression in pericytes. We therefore reanalyzed the dataset to include the vCAFs/pericytes in the CAF subcluster analysis (**New Fig. 2**). vCAFs/pericytes identified in cluster 5 comprise a small number of cells compared to the other CAF subsets. We annotated cluster 5 as vCAF/pericyte based on shared marker expression between these two populations, as the limited cell number reduces the resolution needed to definitively distinguish these two closely related populations.

Analysis of *tdTomato* distribution among all CAF clusters indicated that *tdTomato*$^+$ cells contribute predominantly to cluster#0 (myCAF1) and partially to cluster#2 (myCAF2) and cluster#5 (vCAF/pericyte), while very few *tdTomato*$^+$ cells are detected in clusters corresponding to myCAF3, iCAFs, and proliferating CAFs. GO pathway analysis further revealed that only myCAF1 is enriched in skeletal-related pathways compared to myCAF2 and vCAF/pericyte (**New Fig. 2J** and **Suppl. Fig. 4**). Importantly, unbiased differential expression analysis of our clustering identified myCAF1-specific top markers that include canonical osteoblast-associated genes (**New Fig. 2K**). List of top genes expressed in each cluster is shown in Supplementary Table 1.

3. In Supp Fig 3 the authors claim that "Nuclear localization of Osx was only observed in the TdT+OsteoLin cells, consistent with its role as an active transcription factor (Fig. 3A), but not in the GFP$^+$ tumor cells (Suppl. Fig. 3)." The authors should add brightfield images to show the cells in the frame.

As requested, we provide below brightfield images of GFP$^+$ tumor cells in vitro showing the absence of detectable Osx (see below). However, since we provide Osx staining of tumor tissues *in vivo*, this panel has been removed from the revised manuscript.

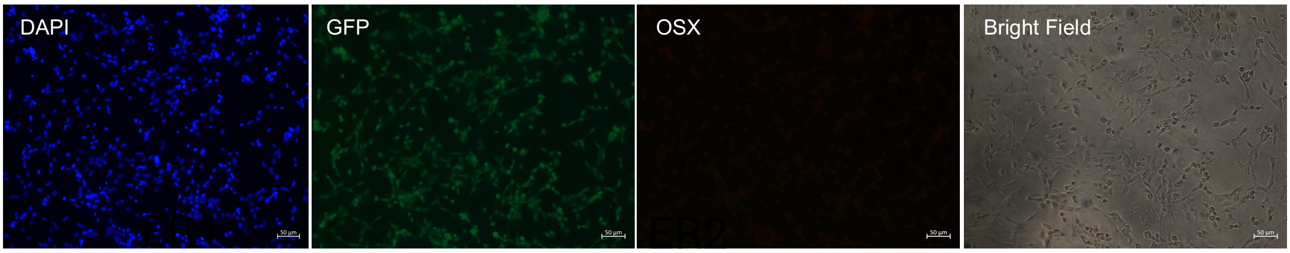

Reviewer #1*-IF staining of Osx in PyMT-BO1 cells in culture showing no detectable expression.*

4. In Figure 3 the authors claim that "Osx nuclear staining was observed in subsets of PDGFRα$^+$ and αSMA$^+$ cells across all tumor models." To strengthen this claim the authors should add staining with markers such as CD45, Epcam etc.

We apologize for not clearly describing the tumor cell markers used in our mIHC analyses. In the spontaneous PyMT-MMTV model, tumor cells were identified by Epcam staining, whereas GFP labeling was used to detect the 4T1 tumor cells (**New Suppl. Fig. 6B**). The PyMT parental line does not express Epcam and is not fluorescently labelled, thus tumor-specific staining could not be included in this model.

We previously demonstrated that mature CD45$^+$ immune cells do not express Osx, therefore, we did not include CD45 in mIHC staining (PMID: 32755539). Nonetheless, we have now incorporated CD45 staining in the initial characterization of OsxTdT$^+$ cells infiltrating the TME (**new Fig. 1A**).

5. Figure 5 – The authors state that "osteoprogenitor-derived Osx$^+$myCAFs promote tumor progression, at least in part, through the release of MMP13." In order to strength their claims, it would be interesting to test the effect of MMP13 inhibitor on tumor growth in the genetic model (OsxCre;TdT tumor bearing mice) as the effect of MMP13 inhibitor on NMF expressing Osx is not fully convincing.

We apologize for the lack of clarity in describing the effects of MMP13 inhibition on tumor growth. The impact of MMP13 inhibitors in breast cancer has been previously reported, including delayed primary tumor growth and reduced metastatic dissemination (PMID:35042959; PMCID:3256168). Because the OsxCre;TdT mouse is a lineage tracing model, assessing MMP13i in these animals would replicate these established findings.

Instead, our study specifically addresses whether MMP13 mediates the pro-tumorigenic effects of Osx$^+$ CAFs. First, conditioned medium from MMFOsx$^+$ cells increased tumor cell growth and spreading in vitro, effects that were abolished in the presence of the MPP13 inhibitor (**Fig. 5C-G**). Second, in a 3D co-culture system, the MMP13 inhibitor reduced tumor growth only in the presence of MMFOsx+ cells (**Fig. 5H-J**). Third, in vivo delivery of the MMP13 inhibitor selectively reduced tumor growth in mice co-injected with MMFOsx+ cells, but not in those co-injected with MMFctr (**Fig 5K-M**). Finally, we have added a new experiment showing that genetic silencing of MMP13 in MMFOsx$^+$ significantly reduced tumor growth compared to scramble controls (**New Fig. 5N-O**).
Collectively, these findings indicate that OsteoLin-CAFs promote tumor progression, at least in part, through MMP13 secretion.

6. Figure 7 – The authors performed mIHC on a TMA of human breast cancer, stained for Osx, PDGFRa and aSMA and found triple positive cells. However, they didn't stain for other CAF markers, and didn't rule out the possibility of Osx expression in another CAF subsets. Moreover. They should quantify the fraction of Osx+ myCAF population.

We thank the reviewer for this suggestion. We performed additional mIHC analyses using human breast cancer biopsies incorporating established markers that define CAF subtypes (PMID: 38683161): PDGFRα$^+$αSMA$^+$ cells as myCAFs, PDGFRα$^+$COL14A1$^+$ cells as iCAFs, and αSMA$^+$MCAM(CD146)$^+$ cells as vCAFs.

Using this classification, we quantified Osx$^+$ cells across CAF subtypes (**New Fig. 7A**). Osx$^+$ h-myCAFs constituted 47% of the total stromal compartment, whereas Osx$^+$ h-iCAFs accounted for 10%, and Osx$^+$ h-vCAFs represented only 0.04% of the total stroma. These results support the conclusion that Osx is preferentially expressed in the myCAF population in human breast cancer tissues.

Minor comments
1. Supp1a – Axis names should be added to the gating strategy of the FACS plots.

We apologize for omitting this info, and figures have been modified accordingly.

2. Supp1b – Why do the authors gate osteolingeage cells as CD31+? Is this typo?
Thank you for finding this typo. It has been corrected.

3. Figure 1h – The authors isolated CAFs by plating the tumor mass on tissue culture plate instead of FACS sorting directly from the tumor. It is established that fibroblasts are very plastic and change their phenotype in vitro, usually acquire a myCAF phenotype. Is there a reason for using this method for CAFs isolation rather than FACS sorting?

Co-injection experiments with CAFs and tumor cells require a large number of cells (10^5 CAFs) per recipient mouse. Sorting CD45^{neg};OsxTdT$^+$ cells directly from the tumor microenvironment yields an insufficient number of viable cells for in vivo co-injection experiments, necessitating pooling tumors from multiple donors for a single recipient mouse. Because of these technical limitations, we used an established protocol for CAF isolation and expansion, routinely used in the laboratory of Dr. Longmore at WashU (PMCID:4909540), which enables the enrichment of CD45^{neg};OsxTdT$^+$ and CD45^{neg};OsxTdT^{neg} cells for functional comparisons.
Further characterization of these two populations demonstrated a comparable distribution of myCAF, iCAF, and vCAF subsets, with myCAFs representing the most abundant subset in both TdT$^+$ and TdT^{neg} fractions (**New Fig. 3A, B**). Importantly, only CD45^{neg};OsxTdT$^+$ cells promoted tumor growth in co-injection studies (**New Fig. 3C**). These results support unique tumor-promoting features of CD45^{neg};OsxTdT$^+$ cells.

4. Figure 2b- although the authors sorted mesenchymal cells and gated the negative populations, it seems there is contamination of epithelial cells, macrophages and endothelial cells. They do address this issue in the text, but the fraction of epithelial cells is quite similar to the number of fibroblasts. It raises concerns regarding the identity and quality of the isolated mesenchymal cells.

CAFs only represent 3-5% of total cells in the PyMT tumor model, and by flow cytometry, OsxTdT$^+$ cells comprise ~20% of the PDGFRβ^+ CAFs. Given the limited number of these populations in the tumor, some degree of tumor cell contamination is expected when sorting TdT$^+$ cells. For scRNA-seq analysis, this does not compromise interpretation, as distinct tumor, immune, and stromal clusters can be computationally separated.

We recognize, however, that purity is more critical for functional co-injection studies. For this reason, we used the above-mentioned CAF enrichment protocol (PMCID:4909540). Briefly, dissociated tumor tissue was plated for 30 minutes to allow preferential adherence of stromal cells, followed by removal of non-adherent tumor and immune cells. This approach enabled efficient isolation of CD45^{neg};OsxTdT$^+$ cells and CD45^{neg};OsxTdT^{neg} for functional comparison in co-injection experiments.

5. In line 154 there is a typo in the text, instead of cluster 4 they wrote 8.

This typo has been corrected.

6. Figure 6 – The authors analyzed scRNA seq data of human breast cancer patients. In the mouse data they showed Osx was highly enriched in mycaf-1 cluster. However, in the human data they don't show the expression of this marker, although they analyzed the top 50 DEG. Could the authors check the expression level of Osx and explain why it didn't come up as one of the DEG? If the authors aim to show the similarity between Osx+ mycaf and h_mycaf1, they should at least assess the levels of Osx/Sp7 in human scRNA-seq data.

We apologize for the confusion. In the mouse scRNAseq dataset, we used tumors isolated from OsxCre$^+$;TdT$^+$ lineage tracing mice, and tracked *tdTomato* across CAF clusters, which shows enrichment in

the myCAF1subset. Because Osx is a transcription factor, its expression is difficult to detect in scRNAseq datasets, murine or human. Thus, to directly assess Osx protein localization, we performed mIHC in human breast cancer tissues. Results confirm scRNAseq analysis and show a higher proportion of Osx^{+} cells within the human myCAF subset compared to other CAF populations (**New Fig. 7A-B**).

Reviewer #2 (Remarks to the Author): with expertise in CAFs

In this manuscript Furesi et al. address an important and topical question in cancer-associated fibroblast (CAF) research, regarding whether the source of CAFs is a determinant of their functional contribution to tumour progression. The authors identify a novel role for osteoblast precursors forming a subpopulation of myofibroblastic CAFs in breast cancer with pro-tumorigenic properties, driven at least in part by Osx/Sp7 mediated expression of MMP13. The manuscript presents some interesting findings that may provide novel insight into identifying important tumour promoting components of the tumour microenvironment and therefore present novel opportunities for therapeutic intervention.
Overall, the findings are compelling and novel, particularly in terms of the functional analysis performed using mouse models. However, there are some issues that would need to be addressed before I could recommend that this manuscript was published. The main areas of concern are regarding the scRNA-seq analysis, validation in human tissues and potential discrepancies between some of the mxIHC data presented and the conclusions drawn. Further details of areas that should be addressed are detailed below.

We thank the reviewer for finding our work compelling and novel and for highlighting its significance. We have addressed the concerns related to scRNAseq analysis and mIHC data below.

Introduction
The introduction contains inaccuracies in its description of normal fibroblast markers (line 53). Specifically, the statement that SMA is expressed by normal fibroblasts is incorrect, as SMA is not a typical marker for fibroblasts in normal tissues. Similarly, PDGFRβ and FSP1 are not ubiquitously expressed in fibroblasts. Furthermore, these claims are presented without supporting references, which undermines the scientific rigor of the introduction. The authors should revise these statements to accurately reflect the current literature and provide appropriate citations to support their claims.

We apologize for the inaccuracies. The introduction has been revised to accurately reflect the current literature.

Results

Figure 1
The authors effectively demonstrate that bone marrow-derived cells constitute a small subset (approximately 20%) of PDGFRβ+ CAFs in orthotopic tumours. However, further control experiments would help to determine whether this is unique to the TME. For instance, the percentage of PDGFRβ+ cells originating from the osteolineage in normal mammary tissue is not reported.

We thank the reviewer for this suggestion. We have now included IF staining of PDGFRβ and Osx in the mammary glands from non-tumor-bearing OsxCre;TdT mice. We observed minimal TdT signal and did not detect elongated OsxTdT^{+} cells (**New Suppl. Fig. 1C**). Together with the bone marrow transplantation experiment (**Fig. 1G-I**), these findings support the conclusion that OsxTdT^{+} cells are recruited from the bone marrow to the TME during tumor progression.

The experimental design in panels H-K raises several questions. The manuscript does not specify how long the cells are cultured prior to re-injection, and there is inconsistency in the tumour models used (PyMT-B01 in panel J versus PyMT in panels I and K) without any justification for this change.

We apologize for the limited experimental details on CAF isolation and for not clarifying the rationale for using different tumor models. As noted in response to Reviewer 1, CAFs were isolated using an established enrichment protocol rather than direct sorting from the tumor mass (detailed experimental information included in material and methods). We used the PyMT-B01 model when directly comparing OsxTdT^{+} and OsxTdT^{neg} cells because this tumor line expresses GFP, allowing exclusion of contaminating tumor cells when sorting the TdT^{neg} CAF fraction.

Additionally, from the data presented it is not clear whether the TM-TdT− stromal population, isolated using negative selection, is a pure CAF population and likely includes endothelial or mural cells. This may confound the conclusions drawn regarding the osteolineage CAFs having enhanced tumour-promoting properties compared to CAFs derived from other precursors. Employing a positive selection strategy, such as isolating CD45−PDGFRβ+TdT+/− cells, to ensure the same population of cells is being analysed would yield more definitive results.

We thank the reviewer for this important point. We have now included the characterization of OsxTdT^{+} and OsxTdT^{neg} stromal populations isolated using the CAF enrichment protocol (**New Fig. 3A-C**). CAF enriched cultures and the dissociated tumor mass were analyzed by flow cytometry after exclusion of dead cells, GFP^{+} tumor cells, CD45^{+} immune populations and Ter119^{+} red blood cells (**see figure for Reviewer#2 below and Suppl. Fig. 5B-D for the full gating strategy**). CD31^{+} cells were detected in the tumor mass but not in the CAF cultures.

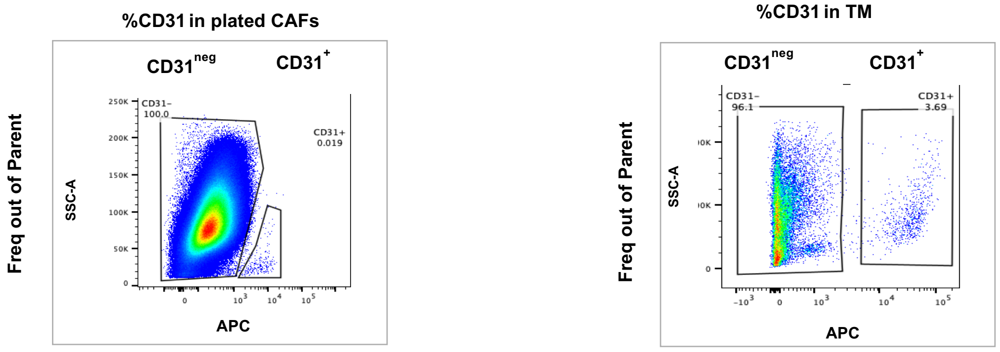

_Reviewer #2__- Flow cytometry analysis showing CD31+ cells in enriched CAF cultures (left) compared to the dissociated tumor mass (TM) (right)._

We have also included subset analysis of CAF enriched cultures and found similar subset distribution between the OsxTdT^{+} and OsxTdT^{neg} cells, with over 75% of cells expressing myCAF markers and the remaining cells expressing iCAF and vCAF markers (**New Fig. 3B)**. Interestingly, only the OsxTdT^{+} cells, which express Osx (**Fig. 3D**), increase tumor growth, supporting a functional role for Osx expression rather than differences in CAF subset composition (**New Fig. 3C).**
As mentioned to Reviewer 1, direct positive selection of CD45^{neg}PDGFRβ^{+}TdT^{+} cells yields insufficient number of cells for co-injection experiments and would require pooling tumors from many mice. For this reason, we employed the CAF enrichment strategy to obtain sufficient, phenotypically comparable CAF populations for our functional studies.

The interpretation of the results in panel K is not fully supported by the data. The conclusion that osteolineage CAFs acquire tumour-promoting properties in the tumour microenvironment does not align with the co-injection experiments. If this was the case, then these cells would still acquire tumour promoting properties when co-injected into the tumour. Perhaps an alternative conclusion would be that only a subset of osteolineage mesenchymal cells are recruited and activated as tumour-promoting CAFs, so by co-injecting all bone marrow stromal cells this selection is bypassed.

We agree with the reviewer. We modified our conclusion and discussed accordingly.

Figure 2
The single-cell RNA sequencing analysis lacks adequate controls for batch effects, which is particularly concerning given that cells were pooled from different cohorts of mice. The absence of batch integration in the analysis could lead to clustering artifacts that confound the interpretation of TdT+ versus TdT− populations. The authors should re-analyze the data using established batch correction methods (e.g. 31178118) and provide detailed metadata, including mouse identifiers, processing dates, and pooling. As far as I can tell, the analysis relies on a single pooled sample (effectively n=1) for defining differentially expressed genes (DEGs) and generating the “OSX+ myCAF” signature, which is a significant limitation.

In our initial scRNAseq analysis, batch correction was not applied because cluster distribution of the *tdTomato*$^{+}$ cells derived from tumors in OsxCre;TdT+ mice was consistent across independently sorted GFP^{neg};CD45^{neg};OsxTdT^{+} populations (pooled from 3 tumors) and the GFP^{neg};CD45^{neg} stromal cells, which included OsxTdT^{+} and OsxTdT^{neg} cells, (pooled from 3 additional tumors). To formally address the reviewer concern, we reanalyzed the dataset using harmony for batch integration (PMID:31740819). The overall distribution of *tdTomato*$^{+}$ cells across clusters remained consistent with our initial analysis, without shifts in cluster representation suggestive of batch-driven artifacts.

As stated above, we isolated and pooled GFP^{-}CD45^{-}OsxTdT^{+} stromal cells from three OsxCre;TdT+ mice prior to library preparation and sequencing. In parallel, we pooled total GFP^{-}CD45^{-} stromal cells from an additional three OsxCre;TdT+ mice and processed them separately. Pooling samples is a widely used strategy in single-cell studies that preserves biological heterogeneity while enabling reliable detection of differentially expressed genes (DEGs) at single-cell resolution within each cluster (Gubin et al., PMID: 30445041; Hao et al., PMID: 37146584).

The full metadata, including sample origin and processing information, is available online (GSE292888 - access restricted to reviewers). All mice were age and sex-matched, and tumors were pooled and processed together on the same day to further minimize technical variability.

The flow cytometry panel also requires further validation, using qPCR or RNA-seq to verify that the populations gated in this way in fact represent the populations identified by scRNA-seq. As presented, the TdT+ cells are determined to be predominantly myCAF based on PDGFRA+ and the absence of alternative iCAF (Ly6c) and vCAF (CD146). PDGFRA is not a de-facto myCAF marker and is in fact typically downregulated on myCAFs compared to quiescent or universal fibroblasts.

We validated the scRNA-seq findings using flow cytometry and immunohistochemistry with antibodies suitable for these approaches in our tumor model. Importantly, the same flow cytometry panel and gating strategy were previously validated in the spontaneous PyMT-MMTV model to identify a subset of senescent myCAFs (PMID:38683161). In our study, the panel included PDGFRβ and PDGFRα together with exclusion of Ly6C and CD146 to distinguish the myCAF subset from iCAFs and vCAFs, as previously reported (PMID:38683161; PMID:38683144).

In addition, we included orthogonal validation of the scRNA-seq findings. Consistent with the ECM-enriched signature identified by scRNA-seq, functional analyses demonstrated ECM remodeling in tumors co-injected with OsteoLin-myCAFs or following Osx deletion in the stromal compartment (**New Fig. 4**), Furthermore, mIHC in human TNBC samples showed a higher proportion of Osx^{+} cells within the h-myCAF subset compared to h-iCAFs and h-vCAFs, providing protein-level validation (**New Fig. 7A,B**).

Figure 3
The proportion of Osx+ cells within the myCAF population (and other cell populations) should be quantified here. There appear to be discrepancies between the immunohistochemistry and flow cytometry, likely due to differences in gating strategies and marker selection. For example, In the 4T1 model it is evident that the Osx staining is found in cells that are both SMA+ and SMA-, So doesn’t seem to be limited to myCAFs. In the PyMT image shown, the SMA staining profile is indicative of vessels (not CAFs) and again there are clearly OSX+ SMA+ cells here that represent OSX+ mural cells not myCAFs.
The immunohistochemistry data show OSX+ cells in both SMA+ and SMA− populations, while the flow cytometry analysis pre-gates for PDGFRβ+. This suggests that the current approach may overestimate the number of OSX+ myCAFs due to the inclusion of mural cells. The authors should consider repeating the immunohistochemistry with PDGFRβ co-staining and reconciling these findings with the flow cytometry data.

The reviewer raises an important point. We re-clustered the scRNAseq dataset to include vCAF/pericyte, and we found that *tdTomato* expression is primarily enriched in myCAF1, with lower contribution to myCAF2 and vCAF/pericyte clusters (**New Fig. 2G**). Flow cytometry of CD45^{neg};OsxTdT^{+} cells isolated from the TME confirmed these results, showing that ~ 75% of OsxTdT^{+} cells express myCAF markers, while smaller fractions display iCAF (~9%) or vCAF (vCAF1/vCAF2; ~6% each) markers (**New Fig. 2I**).

In addition, mIHC analysis of human patient biopsies showed that Osx^{+} h-myCAFs represent about 47% of the total stromal cells, while Osx^{+} h-iCAFs and Osx^{+} h-vCAFs account for ~10% and ~0.04% of the total stroma, respectively (**New Fig. 7A, B**).

Although PDGFRβ co-staining would have been informative, high background with the available PDGFRβ antibody prevented its reliable use in mIHC. Therefore, we used previously established CAF marker combinations reported in similar studies (PMID:38683161). Furthermore, while we would have liked to include αSMA as an additional CAF marker for flow cytometry to match mIHC analysis, intracellular detection of αSMA requires fixation and permeabilization, which abolishes tdTomato fluorescence.

In panels C&D, the definition of "OSX+ myCAFs" is unclear, as the authors note that Osx expression is not accurately detected in the scRNA-seq data. How are Osx+ myCAF defined?

We apologize for any confusion. Because Osterix is a transcription factor and the gene encoding for Osx (*Sp7*) is not detected in our scRNA-seq, we identified *TdTomato*-expressing myCAFs as Osx^{+}myCAFs because *TdTomato* expression is driven by Sp7Cre-mediated lineage tracing. However, we recognize that this terminology might be inaccurate and have renamed this population as OsteoLin-myCAF based on their lineage origin and transcriptional profile.

The method described for identifying DEGs ("avg_log2_FC *pct.1") is unconventional and not clear regarding the threshold for calling a gene differentially expressed. Furthermore, it is not clear what comparison is being referred to for identifying genes used to generate the Osx+ myCAF signature. The most appropriate approach would be to compare TdT+ and TdT− cells within the same cluster (e.g., myCAF-0) and to use conventional criteria for identifying DEGs, such as adjusted p-values and fold-change cutoffs.

We apologize for any confusion. We performed the differential expression analysis (DEA) and identified DEGs using conventional methods. Specifically, we used the Wilcoxon test and the FindAllMarkers function for DEA. DEGs were defined based on the average log2 fold change and adjusted p-values, following standard criteria.

The "avg_log2_FC*pct.1" formula was used for selecting the osteolineage gene signature. However, during the revision, we realized that pct.1 alone may not be the optimal metric for selecting signature genes in myCAF1, given some overlap with other CAF clusters. Therefore, we updated the weighting metric to "pct.1 – pct.2" which prioritizes genes that are not only highly expressed in myCAF1 but are also more uniquely expressed in this cluster compared to others.

Regarding the suggestion to compare *tdTomato*$^{+}$ and *tdTomato*^{neg} cells within the myCAF1 cluster, the latter population is scarcely represented within this cluster, making such a comparison not statistically meaningful. Such an imbalance could lead to unreliable DEG estimates and inflated statistical variance.

To more robustly define cluster-specific transcriptional programs, we included GO analysis comparing myCAF1 vs all clusters (**New Fig. 2J**), myCAF1 vs myCAF2, and myCAF1 vs vCAF, along with the reciprocal comparisons (**New Suppl. Fig 4**). These analyses revealed that only myCAF1, which has the highest representation of *tdTomato*+ cells, uniquely expresses skeletal development and ossification pathways compared to the other clusters.

Figures 4 & 5
These figures are well-presented and do not raise major concerns.

Figure 6
The conclusions drawn in this figure are contingent on the definition of OSX+ CAFs and their associated DEGs, which, as noted above, require further validation and clarification.

Please see the response to Figure 3.

Figure 7
The data showing an increased percentage of myCAFs that are OSX+ and/or MMP13+ in non-responders is intriguing. However, the classification of myCAFs as "PDGFRA+/αSMA+" is ambiguous. If the intention is to identify double-positive cells, the notation should be clarified (e.g., PDGFRA+αSMA+). If the analysis

includes single-positive populations, it should be repeated using only double-positive cells, as the others are more likely to represent iCAFs or mural cells.

We apologize for the lack of clarity in labeling these cells. The analysis included only PDGFRα$^+$ αSMA$^+$ double-positive cells, and the labeling has been modified. We have also added additional markers to identify h-iCAFs and h-vCAFs. Results confirmed a higher proportion of h-myCAFs expressing Osx than h-iCAFs or h-vCAFs **(New Fig. 7A,B).**

The use of CIBERSORT to distinguish between OSX+ and OSX− myCAFs requires robust validation, given the potential for transcriptional overlap. The authors should describe the validation and quality control steps undertaken for this analysis and, ideally, confirm the results using ground truth validation (e.g. by IHC or flow cytometry); or at the very least using pseudobulk transcriptome simulated by aggregation of scRNA-seq data (as described in the original CIBERSORT publications). Additionally, the survival analysis requires further validation in independent cohorts and by examining differences across breast cancer subtypes.

We agree that deconvolution approaches such as CIBERSORT can be challenging when distinguishing closely related CAF subsets with partially overlapping transcriptional programs. In response to this concern, we no longer use CIBERSORT-based deconvolution in the revised analysis. Instead, we evaluated enrichment of the OsteoLin-myCAF gene signature using single-sample Gene Set Enrichment Analysis (ssGSEA), which evaluates gene set enrichment in each tumor sample independently without attempting to estimate precise cell fractions. This approach avoids potential inaccuracies associated with deconvolution of closely related stromal populations.
The OsteoLin-myCAF signature was derived from genes selectively enriched in the OsteoLin-myCAF cluster in our murine scRNA-seq dataset. Importantly, independent validation was performed using mIHC in human breast cancer samples, which confirmed enrichment of Osx expression within the h-myCAF population (**New Fig. 7A,B**).

General Recommendations
Overall, the manuscript would benefit from greater precision in marker selection and description, improved methodological rigor (including batch effect controls and consistent use of tumour models), and more transparent statistical reporting. The language should be clarified to avoid ambiguous terms and correct typos. It would also be useful for the code used to perform the bioinformatics analysis to be provided for review.

We appreciate the reviewer's overall assessment and have revised the manuscript to improve marker definition, methods and statistical transparency, with additional statistical details now provided in each figure legend. The text has been carefully edited to correct typographical errors and remove ambiguous terminology.
The bioinformatics code is available via the link provided in the "Data and Code Availability" section of the revised manuscript

Reviewer #3 (Remarks to the Author): with expertise in breast cancer, bone biology

The manuscript by Furesi et al. identifies a novel bone marrow-derived Osx+ osteoprogenitor cell which becomes a myofibroblast CAF (myCAF) to promote breast cancer progression driven by MMP13. These findings are timely and important for the field, identifying a new population of CAFS that promote breast cancer progression. Classifications of CAFs are complex, and the data included here further characterize these subsets and identify the progenitor cells involved. The studies are well-designed, using bone marrow adoptive transfer to show the origin of the cells, and the data for this are compelling. While the findings are interesting and novel, there are several areas to address:

We thank the reviewer for finding the manuscript novel and important, and for appreciating the experimental design. We are also grateful for all the suggestions to improve the manuscript.

Major
1. The population of osteoprogenitor cells that are identified as tumor-promoting, Osx+myCAFS, have very low abundance in the primary tumor (0.8-1.2%), which calls into question their relative importance. As indicated by the authors, >90% of the Osx+ cells in the TME were immune populations. What is the relative contribution of the Osx+myCAFS vs other tumor-promoting cells in the TME? In that vein, if Osx and

MMP13 have a functional role in breast tumor progression, as suggested by the data, then what contribution do the Osx+CD45+ or Osx+CD45- cells play in the TME? Are they also pro-tumorigenic?

CAFs represent about 3-8% of cells in the TME of breast cancer tumor models (PMID: 32455670, PMID: 30514914), and their proportion can vary depending on the tumor type and stage. While OsteoLin-myCAFs constitute a small fraction of the total tumor mass, low abundance does not preclude functional significance. Similar to our findings, prior studies have identified transcriptionally distinct CAF subsets, including senescent CAFs and MHCII^{+} CAFs (PMID:38683161; PMID:31197017), which represent small CAF subpopulations, and yet exert robust tumor-promoting effects.

Importantly, we performed new experiments in which Osx was deleted in αSMA^{+} stromal cells (aSMACre^{ERT2};Osxfl/fl) and observed a significant reduction in tumor growth compared to littermate controls (**New Fig. 4H-K**). These data indicate that Osx expression in CAFs functionally contributes to tumor progression, independent of overall abundance.

Regarding the contribution of CD45^{+};OsxTdT^{+} cells to tumor progression, we previously showed that they do not support tumor growth and do not express Osx (PMID:32755539). Thus, the tumor-promoting effects observed in this study are attributable specifically to the CD45^{Neg}Osx^{+} stromal population.

2. The experiment comparing the Tdt+OsteoLin vs Tdtneg cells is a nice control (Fig. 1J); however, other critical controls are lacking. Please include controls to show that the Tdt OsxCre system is not leaky (i.e., that the Tdt is specifically labeling Osx+ cells and is only active once transitioned to standard diet).

To address the concern regarding potential leakiness of the OsxCre;TdT system, we performed IF analysis of the mammary gland isolated from naïve mice kept on doxycycline to suppress Cre activation until 6 weeks of age (no Cre activation). In parallel, we analyzed mammary gland isolated at 6 weeks of age following doxycycline withdrawal at weaning (Cre activation). No TdT signal was detected in mice maintained on doxycycline (see Figure for Reviewer #3 below). After doxycycline removal, only rare OsxTdT^{+} cells were observed in the mammary gland, but at a much lower frequency than in the TME. Similar findings were reported in inguinal fat at 6 weeks and 12 weeks of age using the same mouse model (PMID: 35318397). These results indicate that this model is suitable for studying the role of OsxTdT^{+} cells in the TME.

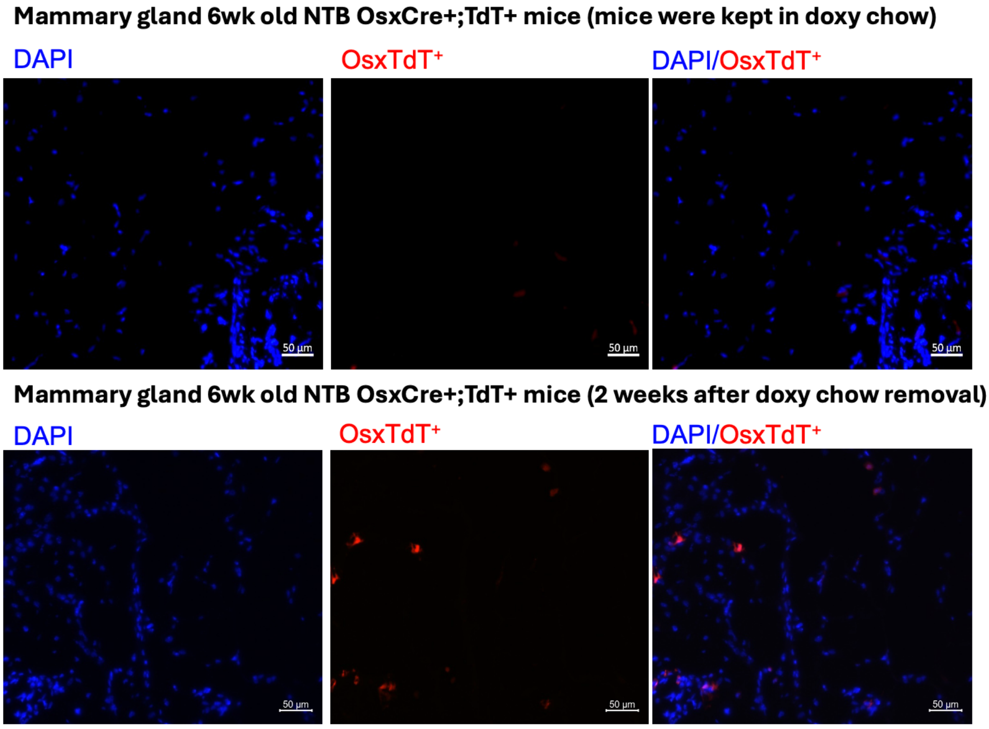

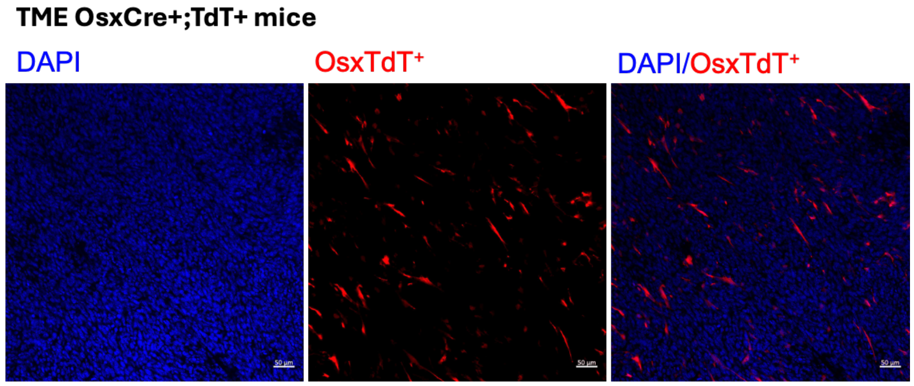

__Reviewer #3__ Representative IF images showing OsxTdT^{+} cells in the mammary gland isolated from OsxCre;TdT^{+} mice before (TOP) and after doxycycline withdrawal (MIDDLE), or infiltrating the tumor microenvironment (TME) of orthotopic PyMT tumors (BOTTOM PANELS).

Appropriate negative controls are also needed for all staining (e.g., PDGFRalpha and beta, etc). Controls for TdtnegOsx+ and negative controls for Osx staining are needed for Fig. 3.

We apologize for the lack of clarity regarding staining controls. In mIHC, negative controls were generated by performing the complete staining and stripping protocol while omitting primary antibodies, now shown in **new Suppl. Fig. 6A**.
To further validate antibody specificity for Osx, we performed Osx staining on healthy tonsil tissue as a negative control and compared it with TNBC tissue samples (**New Suppl. Fig. 10B**)
In addition, for IF staining, we included secondary antibody-only controls to exclude nonspecific background staining (**new Suppl. Fig. 1**).

3. There is a strong emphasis on ECM remodeling and how Osx+myCAFS have ECM remodeling properties, but this is not demonstrated in the manuscript. There are no functional collagen remodeling assays included, only gene signatures. See lines 336, 339, and 318. While the data confirm that Osx+myCAFS arise from the bone marrow, and promote tumor progression, there are no functional studies confirming that this is happening because the myCAFs alter the ECM. The data in Fig. 4 suggest that the effect of the Osx+myCAFS on tumor cells is through aggregation rather than proliferation, but this is not necessarily due to changes in the ECM. There either needs to be data demonstrating this or the language around ECM remodeling needs to be tempered.

The reviewer is correct that functional evidence of ECM remodeling was not sufficiently demonstrated in the original submission. To address this point, we have included second harmonic generation images and picrosirius red for collagen staining of orthotopic tumors showing changes in ECM in mice co-injected with MMFOsx^{+} versus MMFctr (**New Fig 4**). We have also included new data showing changes in tumor growth and collagen staining in aSMACre^{ERT2};Osxfl/fl mice compared to their WT counterpart (**New Fig 4**). These results indicate that Osx expression in CAFs affects ECM organization. Consistent with these observations, scRNAseq analysis revealed enrichment of ECM-remodeling genes in the OsteoLin myCAF subset (**New Suppl. Fig. 8**).

4. The human data provided in Figure 7 suggests that higher Osx+myCAFs are significantly associated with reduced 10 yr survival in BC patients. Do the Osx+myCAFS increase with tumor growth in the pre-clinical models? The human data suggest that this population may expand with tumor progression, but it is not clear. Related to this, are the Osx+myCAFS the same cells as the CD15-CD34-Ocn+ cells previously identified, or are these distinct populations? The Ocn+ cells were found to increase as the tumor progressed, which links back to my other question.

To address whether Osx^{+} myCAFs expand with tumor progression, we added new data showing a temporal increase of OsxTdT^{+} cells in circulation with tumor progression (**New Fig 1F)**, suggesting an active recruitment to the tumor site.

It is possible that OsxTdT^{+} cells in circulation are the same as the CD15^{neg}CD34^{neg} Ocn^{+} cells found by Lee KH et al. (PMID:32379371). However, our murine scRNAseq analysis of the myCAF1 cluster, which contains the majority OsxTdT^{+} cells, does not show detectable expression of Ocn (*Bglap*). Similarly, Ocn

expression was not detected in the human scRNAseq dataset, although we cannot exclude that OsteLin-myCAFs may express Ocn while in circulation.

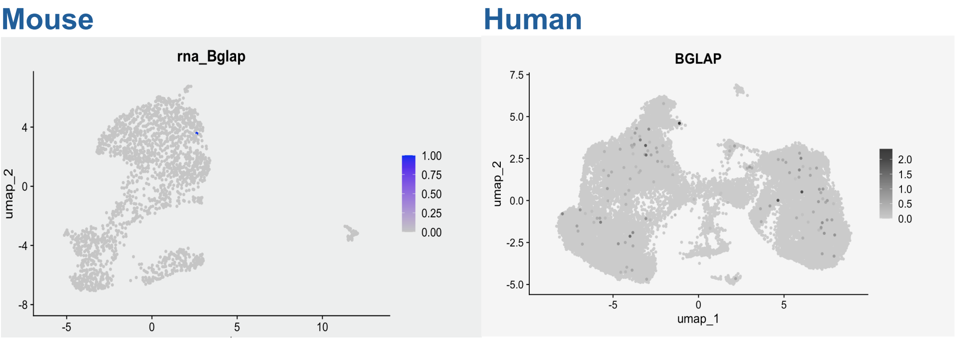

Reviewer #3 *Feature Plots showing lack of expression of Bglap (osteocalcin) in both the mouse and Human scRNAseq datasets.*

5. The finding that the Osx+myCAFS do not become pro-tumorigenic until after they reach the TME is intriguing. Why do you think this is, and more importantly, why/how are these cells recruited to the primary tumor, if they do not inherently promote tumor progression until they have encountered the tumor microenvironment?

We appreciate this insightful question. Another possibility, as also suggested by Reviewer 1, is that there is a small subset of OsxTdT^{+} cells in the bone marrow with intrinsic tumor-promoting abilities. We have included this possibility in the discussion. Addressing the signals involved in the recruitment of bone resident Osx+ cells to the TME is out of the scope of this paper.

6. Should the myCAFS be aSMA and PDGFRa double positive? In multiple images it appears that aSMA and PDGFRa are mutually exclusive, which was confusing (e.g., middle panel of MMTV-PyMT and 4T1 orthotopic images in Fig. 3B). On lines 291 and 292 it says that myCAFS were identified by aSMA and PDGFRa staining and this is again referred to on lines 377 and 378. The populations also appear distinct in the human samples in Figure 7. Please clarify - are these supposed to be co-localized? If so, the colors may need to be adjusted to better demonstrate this. If they are distinct populations, please explain the importance of each and which are actually myCAFs.

We apologize for the confusion caused by the original image. MyCAFs are defined as αSMA^{+}PDGFRα^{+} double-positive cells in mIHC**.** The apparent separation observed in some panels likely reflects technical limitations of mIHC, which involves sequential staining and stripping cycles that can result in minor tissue shifts between rounds. To overcome this technical limitation, we reprocessed the images by segmenting the tissue into smaller regions and realigning the staining cycles using hematoxylin nuclear staining as a reference for accurate registration (**New Fig 3E**). This approach improves co-localization accuracy. In addition, we have included CAF marker staining in human tissues for quantification of Osx^{+} h-CAF subsets (**New Fig. 7A**), showing that Osx staining is highest within aSMA^{+}PDGFRα^{+} h-myCAFs.

7. Please provide data or references demonstrating that MMP13i is specific for MMP13. MMPs are highly redundant, and it is likely that the inhibitor used may target multiple MMPs, or that other MMPs are up-regulated in response to MMP13 inhibition. Do other MMPs change with MMP13 inhibition?

We apologize for not including enough information about the inhibitor. CL-82198 has been characterized as a selective MMP13 inhibitor based on biochemical studies demonstrating preferential inhibition of MMP13 with minimal activity against related MMP family members (PMID:15734645).

Additionally, we now performed genetic silencing of MMP13 in Osx^{+} MMFs using siRNA (**New Fig 5N-O**). MMP13 knockdown significantly reduced the tumor-promoting effects observed in MMFOsx+ co-injection experiments compared to control-transfected cells. These complementary pharmacologic and genetic approaches support a specific functional role for MMP13 in mediating the pro-tumorigenic effects of Osx^{+} CAFs.

8. In the human data, where do the Osx+myCAF equivalent cells cluster on the UMAP projection? The enrichment for the human equivalent of the Osx+myCAF signature is significant as shown in Fig. 6G, but is it a distinct cluster? Please include these cells on a UMAP plot. It says these overlap some with the h-myCAF3 cluster, but the rest of the genes are clustered with h-myCAF1. This is an important point in translating the findings from the mouse to human. It seems like the myCAF populations differ some between human and mouse. Please expand on this. Related to this, on lines 284 and 285 it says the findings demonstrate strong overlap between h-myCAF1 and mouse Osx+myCAFs, but the data do not suggest this (rather they appear to potentially overlap with both h-myCAF1 and h-myCAF3 clusters).

We thank the reviewer for highlighting the importance of accurately mapping the murine Osx^+ myCAF (now defined as OsteoLin-myCAF) population to its human counterpart.
In the revised analysis, we re-annotated the human scRNA-seq dataset to include vCAF/pericyte populations and updated the stromal classification (**New Fig. 6A)**. The human CAF compartment is now organized into vCAF/pericyte (c0), myCAF (c1), iCAF (c2), apCAF (c3/c4), and dCAF (c5) clusters. Projection of the murine OsteoLin-myCAF gene signature onto the human dataset showed that cells with high osteolineage signature enrichment predominantly map to the h-myCAF cluster, with only limited enrichment in the h-vCAF cluster (**New Fig. 6 E-F**). These results indicate that an osteolineage-associated transcriptional program is conserved within the human myCAF population.

Minor
9. The discussion appears to be one large paragraph. It would help to break it up on lines 363 and 383 into new paragraphs.

Thank you for the comment. The discussion has been rewritten and reorganized to improve clarity and flow and to incorporate the newly added data.

10. Higher magnification images of Figure 3 and all staining would be helpful.

Figure 3 has been modified as requested.

11. In Figure 4, why was the MET-1 cell line used instead of 4T1s? Was a similar effect seen in 4T1 cells?

We apologize for not explaining more clearly why we tested the MET-1 cancer line. This cell line was used because we overexpressed Osx in a CAF line derived from Met1 tumors.

12. On line 448, it refers to experiments with either 1:1 or 1:2 stroma-tumor ratios being injected, but only 1:1 was mentioned in the results.

We apologize for the confusion; both ratios were tested. We used a 1:1 stroma-tumor ratio for **Fig. 1K-L** and **Fig. 4D-E**, and a 1:2 stroma-tumor ratio for **Fig. 3C** when comparing $OsxTdT^+$ and $OsxTdT^{neg}$ populations.

13. For all graphs, the percentage on the y axis should indicate % of what was measured (e.g., % parent, % live, %CD45+, etc).

We included the suggested change.

14. Please provide more information about why the treatment timeline was selected, with 10 consecutive days of treatment, followed by 8 days washout before sacrifice.

The use of the MMP13 inhibitor and the administration timeline were based on a previously published study (PMCID: PMC10165729). The mice were sacrificed after observing consistent differences in tumor growth between treated and untreated groups.

15. The correlative data linking the myCAFs with therapeutic response are interesting but should be clarified to indicate that this is specifically for patients receiving chemotherapy with anti-PD-1, which is only a subset of TNBC patients (those with PD-L1+ tumors).

We apologize for this omission. All biopsies analyzed were from patients who received chemotherapy plus anti-PD-1 therapy, according to the Keynote522 regimen (PMID: 32101663). We have clarified in the revised manuscript that the correlative findings apply specifically to this treatment context.

Reviewer #4 (Remarks to the Author): with expertise in breast cancer, bone biology

This manuscript presents compelling evidence for the existence of a novel subset of cancer-associated myofibroblasts in the breast cancer tumor microenvironment, termed Osx$^+$myCAFs, which originate from Osterix-expressing (Osx$^+$) osteolineage precursors in the bone marrow. The authors used lineage tracing, flow cytometry, single-cell RNA sequencing, and spontaneous/orthotopic murine models of breast cancer to demonstrate that Osx$^+$myCAFs retain an osteoblastic gene signature and contribute to tumor growth via MMP13 secretion. By analyzing the dataset and samples from breast cancer patients, the author showed that the presence of Osx$^+$myCAFs in breast cancer tissue correlates with poor therapeutic response and reduced 10-year survival. The study proposes that the expression of Osx in myCAF is not only a phenotypic marker but also has a pro-tumorigenic role. While the characterization of Osx$^+$myCAFs is thorough and detailed in the manuscript, the investigation of pro-tumorigenic functions of Osx$^+$myCAFs is less convincing. Also, the translational potential of using Osx$^+$myCAFs for diagnostic or therapeutic targeting seems to be limited.

We thank the reviewer for appreciating the novelty of our findings. Additional experiments related to the function of Osx+myCAFs have been included in the revised manuscript.

Major points
1. Low percentage of Osx+myCAFs in tumor
In the orthotopic breast cancer mouse model using PyMT cell line, the author found that Osx+myCAFs comprise 0.8-1.2% of the total primary tumor mass (line 103). Then, the author co-injected (Fig. 1I) or co-cultured (Fig. 4E) breast cancer cells with Osx+myCAFs at 1:1 ratio and showed that Osx+myCAFs stimulated tumor growth both in vivo and in vitro. Given the low percentage of Osx+myCAFs in tumor, the pro-tumorigenic effects of Osx+myCAFs seen at 1:1 ratio may raise questions regarding their actual contribution to tumor progression.

We thank the reviewer for raising this important point. While Osx$^+$ myCAFs represent approximately 0.8–1.2% of the total tumor mass *in vivo*, co-injection models commonly employ higher CAF: tumor cell ratios (1:1, 3:1, or 5:1), to ensure measurable biological effects, given the relatively low abundance of CAFs and their limited proliferative capacity compared to tumor cells (PMID: 29507342; 35654839; 28966935). Importantly, even when we used a lower 1:2 CAF-to-tumor ratio, only OsxTdT$^+$ but not OsxTdT^{neg} CAFs enhanced tumor growth, supporting the specific and potent tumor-promoting activity of osteolineage-derived cells.

2. TdT+OsteoLin from BM failed to increase tumor growth
The author demonstrated that TdT+OsteoLin cells isolated from orthotopic PyMT-BO1 stimulated tumor growth while TdT+OsteoLin cells isolated from bone marrow failed (line 134). The difference between these two sets of cells was not investigated in the manuscript. Did TdT+OsteoLin cells from bone marrow have myCAF signature? Did TdT+OsteoLin cells in BM fail to stimulate tumor growth due to the lack of expression of MMP13?

We agree that this is an important question. A detailed analysis of TdT$^+$OsteoLin cells in BM is out of the scope of this manuscript and is addressed in the co-submitted manuscript by Jennifer Zaher et al. They show that BM TdT$^+$OsteoLin cells acquire a fibroblastic phenotype and express MMP13 only after infiltrating tumors in the bone.

3. Timing of the presence of Osx+myCAF
What is the timing of detecting Osx+myCAF in the tumor or in the circulating blood? Does Osx+myCAF contribute to the early or late stages of tumor progression?

We have performed a time-course analysis and found circulating OsxTdT$^+$ cells increasing in the blood at day 7 post-orthotopic tumor inoculation. These results have been included in the **New Fig 1E**.

4. Pro-tumorigenic role of Osx+myCAF
The author concluded from in vitro and in vivo experiments that Osx+myCAF contributes to tumor growth. The analysis of TNBC patients' samples showed that Osx+myCAF may impart therapy resistance, as higher portions of Osx+myCAF were detected in non-responders. The experimental data and clinical data do not appear to support each other. Stimulating tumor growth and inducing therapy resistance are two different functions. Co-injection (cancer cells+Osx+myCAF) mouse model with or without standard-of-care treatment will need to be conducted to investigate the role of Osx+myCAF in therapy resistance.

We apologize for the confusion and modified the discussion to avoid implying that Osx^+ cells are causing therapy resistance and to clarify that the patient data are correlative.

5. The author only used one MMP13 inhibitor (CL-82198) to show that MMP13 is required for Osx+myCAF pro-tumorigenic effects. More evidence will be needed, such as knockdown or knockout of MMP13 in Osx+myCAF, to confirm the finding.

We thank the reviewer for this constructive suggestion and have now included new experiments using siMMP13 in $MMFOsx^{+,}$ confirming that MMP13 knockdown reduces tumor growth (**New Fig. 5N-O**).

Minor points
1. The author used matrix-producing CAF (line 76) and myCAF to refer Osx+myCAF. Although there may be an overlap, it would be good to keep the term consistent in the manuscript.

We made the suggested change**.**

2. For the analyzed 15 TNBC patients receiving standard-of-care treatments, what are these treatments? Including a table listing the treatments in the supplementary data will be helpful. It may be interesting to see if Osx+myCAF causes therapy resistance to a specific treatment.

We thank the reviewer for this comment. All TNBC patients received chemotherapy plus anti-PD-1 according to the KEYNOTE-522 protocol (PMID: 32101663). Because all patients received the same treatment and our analyses were conducted prior to any intervention, we did not provide an additional table listing individual regimens.
